# The Diverse World of Foldamers: Endless Possibilities of Self-Assembly †

**DOI:** 10.3390/molecules25143276

**Published:** 2020-07-18

**Authors:** Samuele Rinaldi

**Affiliations:** Department of Life and Environmental Sciences, Polytechnic University of Marche, Via Brecce Bianche, 60131 Ancona, Italy; s.rinaldi@staff.univpm.it; Tel./Fax: +39-071-2204233

**Keywords:** self-assembly, foldamers, structural investigation, secondary structure, higher-order structures, morphology, helices, vesicles, monolayers, fibers

## Abstract

Different classes of foldamers, which are synthetic oligomers that adopt well-defined conformations in solution, have been the subject of extensive studies devoted to the elucidation of the forces driving their secondary structures and their potential as bioactive molecules. Regardless of the backbone type (peptidic or abiotic), the most important features of foldamers are the high stability, easy predictability and tunability of their folding, as well as the possibility to endow them with enhanced biological functions, with respect to their natural counterparts, by the correct choice of monomers. Foldamers have also recently started playing a starring role in the self-assembly of higher-order structures. In this review, selected articles will be analyzed to show the striking number of self-assemblies obtained for foldamers with different backbones, which will be analyzed in order of increasing complexity. Starting from the simplest self-associations in solution (e.g., dimers of β-strands or helices, bundles, interpenetrating double and multiple helices), the formation of monolayers, vesicles, fibers, and eventually nanostructured solid tridimensional morphologies will be subsequently described. The experimental techniques used in the structural investigation, and in the determination of the driving forces and mechanisms underlying the self-assemblies, will be systematically reported. Where applicable, examples of biomimetic self-assembled foldamers and their interactions with biological components will be described.

## 1. Introduction

### 1.1. Definition and Overview

The research on synthetic oligomers and polymers able to mimic their natural counterparts by adopting well-defined conformations in solution (“foldamers”) [1] started in the late 1990s.

**Figure 1:**
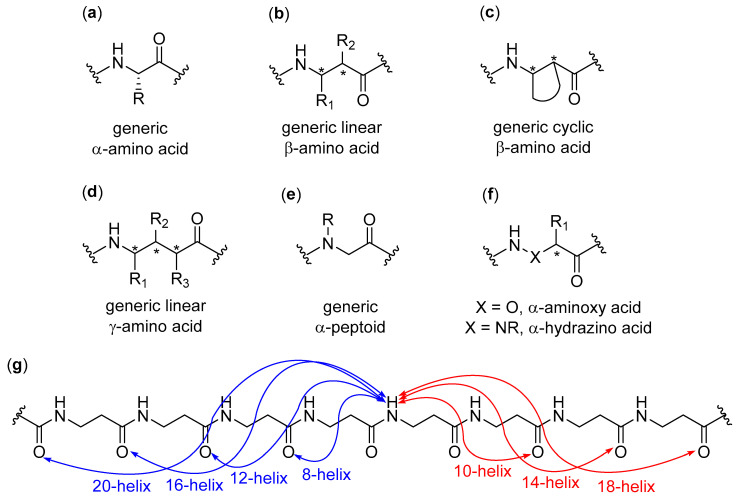
Generic structures of (**a**) proteinogenic α-amino acids, (**b**) linear β-amino acids, (**c**) cyclic β-amino acids, (**d**) linear γ-amino acids, (**e**) α-peptoids, and (**f**) α-aminoxy and α-hydrazino acids; (**g**) The Gellman’s helices nomenclature, reported for a generic β-peptide, in which the numerical indices refer to the number of atoms included in the indicated hydrogen bonded pseudocycles. This nomenclature is also used for higher peptidic homologues. The nomenclature for β-sheets is the same used for α-peptides and it is not reported here (for reviews dealing with the secondary structures of these foldamers, see Refs. [1,2,3,4,5,6,7,8,9]).

**Figure 2:**
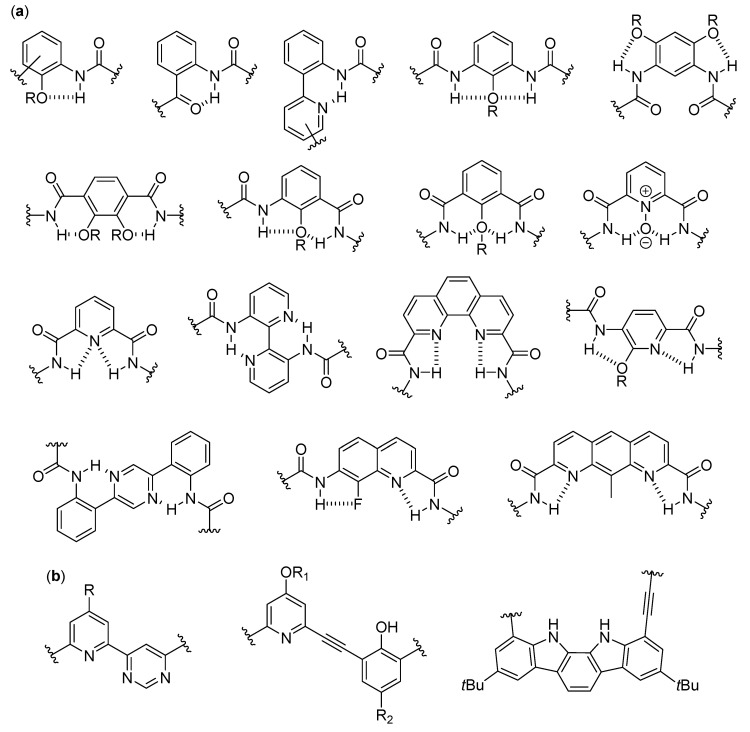
(**a**) Representative monomers for the construction of aromatic amide foldamers and their hydrogen bonding patterns; (**b**) Representative structural motifs for the construction of aromatic foldamers not based on conventional hydrogen bonding (for reviews dealing with the secondary structures of these foldamers, see Refs. [2,6,10,11,12,18,19]).

The fundamental studies in the first two decades led to the discovery of the several secondary structures typical of homo-oligomers made of β, γ and even higher homologues of α-amino acids, as well as α-peptoids [1,2,3,4,5,6,7,8,9], as shown in Figure 1, and various aromatic monomers [2,6,10,11,12], as shown in Figure 2. Within these structural determinations, single-crystal X-ray data obviously played a starring role, but the entire arsenal of mono- and bidimensional nuclear magnetic resonance (NMR) spectroscopies was determined in order to deduce the correct folding in solution and the conformational stability in different solvents, especially when coupled with molecular dynamics simulations. Other important contributions came from circular dichroism (CD) spectroscopy and, even if to a much lesser extent, from infrared (IR) spectroscopy. In the case of β-peptide and aromatic foldamers, the huge amount of information available allowed us to identify and classify all the innate tendencies of the different building blocks, which are deeply connected to the precise monomer structure and substitution pattern. Therefore, now the rational design of the desired secondary structure is possible based on the choice of monomer(s).

As far as helices formed by β-peptide foldamers and their analogues are concerned, it has been shown that an accurate use of the suitable monomers can lead to several different foldings, characterized by their peculiar hydrogen bonding patterns, as shown in Figure 1g. This, in turn, generates helical arrangements that differ by their handedness, diameter, pitch, number of residues per turn, macrodipole (the overall dipole generated by the sum of individual dipoles from the amide groups along the helix axis) and exact placement/directionality of side chains [1,2,3,4,5,6,7,8,9]. As an illustrative example of this ease of control over the secondary structure, when α and β carbons of β-peptide foldamers are tethered in 4, 5 and 6-membered aliphatic cycles with *trans* stereochemistry, the favored secondary structures are the 12- (4 and 5-membered cycles) and 14-helices (6-membered cycles). The introduction of heteroatoms, unsaturations, or bridged bicycles might change the assumed folding, and completely different conformational behaviors were observed for *cis*-substituted β-amino acids [8]. In addition, remarkable variations in β-peptide foldamers folding could easily be obtained by exploiting apparently very small structural modifications, as demonstrated for the simple methylation at C^α^ of a *trans* 5-membered cyclic β-amino acid monomer, that shifted the natural preference toward the rare 8-helix [13,14]. The formal substitution of C^β^ with oxygen or nitrogen atoms in β-peptide analogues (i.e., α-aminoxy or α-hydrazino acids), as shown in Figure 1f, also had a deep impact on their secondary structures [15]. The incorporation of structurally diverse monomers in foldamers with heterogeneous backbones led to a further impressive widening of the attainable conformations [16,17].

The folding of aromatic foldamers could also be rigorously controlled and finely tuned, including the helix handedness, by using the suitable monomer(s). In the case of aromatic foldamers, the immense multiplicity of helices and other arrangements obtained so far has been generated by accurately choosing building blocks with different dimensions, substitution patterns, and directionality of the functionalities linking them to other monomers. The different abilities to give π–π stacking and form the proper intra- and inter-monomer hydrogen bonds were very important, as shown in Figure 2a, even if in many cases conventional H-bonding was absent and the assumed conformation was mainly driven by other forces (e.g., conjugation, unconventional hydrogen bonds, dipole–dipole interactions, etc.), as shown in Figure 2b [10,18,19].

When the knowledge of secondary structures formed by unnatural oligomers was completely coming of age, the natural evolution toward studies regarding higher-order structures and the self-assembly of foldamers also started growing very fast.

### 1.2. Organization of the Review

Due to the exceptional amount of literature available, in this review, we will only report selected examples of all the possible self-assembly modes for foldamers belonging to different classes, which are categorized by their backbones. For every class, the self-assembled structures will be divided according to a numerical/dimensional criterion: from the associations of discrete numbers of foldamer molecules in solution (e.g., dimers, double helices, bundles), to the formation of spherical or mono- and bidimensional aggregates of a large number of molecules (e.g., vesicles, fibers and monolayers), to solid tridimensional structures. The rationale at the basis of the particular self-assembly formed will be discussed, and the experimental techniques used to ascertain both the overall morphology and the arrangement of the single foldamer molecules within the higher-order assembled structure will be reported.

When many articles on strictly related foldamers are present, they will be treated in chronological order, following the reasoning used by the authors and emphasizing the relationships between the structural modifications on the foldamers and the features of the generated self-assembled structure. For two among the most investigated—and reviewed—classes of self-assembling foldamers, namely β-peptides [20,21,22,23,24], as shown in Figure 1b,g, and aromatic oligomers [25,26,27,28,29,30], as shown in Figure 2, we will also put a particular emphasis on the recent advances, as well as on every other aspect not sufficiently covered by the previous reviews, and we will describe the self-assemblies devoted to interactions with biological components. In addition, a separate section will be focused on usually underrated foldamers that do not belong to the two previous macro-categories, either made of less extensively investigated monomers (i.e., γ-amino acids, ureas, etc.) or having heterogeneous backbones (i.e., made of different types of monomer, such as mixtures of α and β-peptides), but that can still show amazing self-assembly properties.

### 1.3. Aim, Scope and Exclusions

The aim of the present review is to offer a panoramic view for non-specialized readers of self-assembling foldamers and the experimental techniques used to determine their mode of aggregation, together with some examples of their potential biological activity. Thus, it is not intended to give a comprehensive and critical coverage of this huge field, nor to only consider recent developments. Selected articles have been reported without taking into account a given time period, but rather putting a special emphasis on more instructive studies where the authors were able to choose the experimental techniques in order to deeply correlate the secondary structure and the experimental variables with the higher-order structure observed. To this end, series of articles that led to a greater amount of knowledge about the effects of modifying the molecular structure and the environmental variables on self-assembly have been privileged.

**Figure 3:**
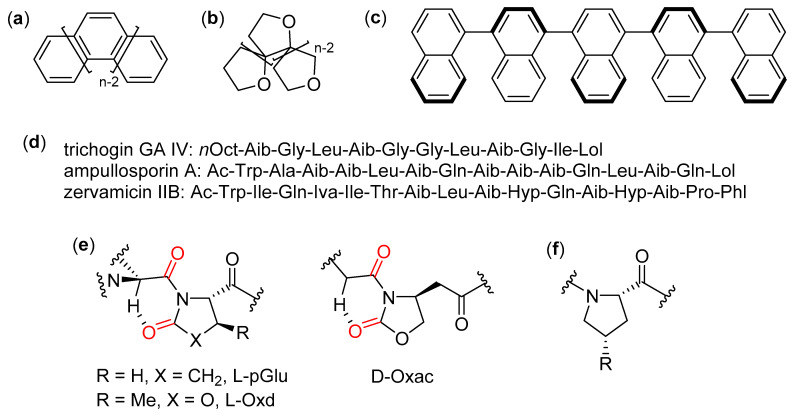
General structures of (**a**) [*n*] helicenes (Ref. [31]), (**b**) polyoxapolyspiroalkanones (Ref. [32]) and (**c**) oligonaphthalenes (Ref. [33]); (**d**) The naturally-occurring peptaibols trichogin GA IV, ampullosporin A, and zervamicin IIB (*n*Oct: *n*-octanoyl, Lol: leucinol, Aib: α-aminoisobutyric acid, Iva: isovaline, Hyp: *trans*-4-hydroxyproline, Phl: phenylalaninol, PheCN: *p*CN(αMe)-phenylalanine, Ac: acetyl; Ref. [37]); (**e**) Preferred *anti* disposition of backbone and side chain carbonyls (in red) and unconventional C=O···H-C hydrogen bonds in L-pGlu, L-Oxd, and D-Oxac homooligomers (Ref. [47]); (**f**) General structure of a modified proline units included in synthetic oligoprolines and collagen model peptides (R can be many different groups; see Refs. [48,49]).

The more restrictive Moore’s definition has been considered here, in order to greatly reduce the otherwise intractable amount of work on oligomers that are usually referred to in the literature as foldamers [2]. According to this definition, a foldamer is “any oligomer that folds into a conformationally ordered state in solution, the structures of which are stabilized by a collection of noncovalent interactions between nonadjacent monomer units”. Following this definition, oligomers, where the folding process is constitutionally impossible (e.g., helicenes [31], polyoxapolyspiroalkanones [32], and oligonaphthalenes [33]), as shown in Figure 3a–c, have not been considered here. Additionally, oligomers built using exclusively non-standard α-amino acids, or a mixture of proteinogenic and non-proteinogenic α-residues, have been almost completely excluded from this review, albeit their interesting studies sometimes included the use of peculiar experimental techniques. For instance, this exclusion applies to α-peptides containing mainly α-aminoisobutyric acid (Aib), for which only a few illustrative examples have been reported here, [34] and chiral α-dialkyl α-amino acids (e.g., synthetic derivatives of naturally-occurring peptaibiotics and peptaibols [35,36,37]), whose self-assembly has been thoroughly studied [38,39,40], especially in the presence of phospholipid membranes [41], as shown in Figure 3d, and to low-molecular-weight gelators (LMWGs) based on short α-peptide derivatives [42,43,44] or pseudopeptides [45,46], which do not fulfill the foldamer definition adopted here. In addition, oligomers made only of different pseudoproline units (L-pyroglutamic acid, L-*p*Glu, *trans*-4-carboxybenzyl-5-methyloxazolidin- 2-one, L- or D-Oxd, and (4*R*)-(2-oxo-1,3-oxazolidin-4-yl)-acetic acid, D-Oxac), as shown in Figure 3e, which are sometimes included in the field of foldamers, have been excluded here [47]. In fact, as also noted in the Moore’s review [2], their secondary structures are simply due to the preference of their amide bonds for the *trans* conformation, which in turn is caused by the repulsion between backbone and side chain carbonyls, as well as the presence of unconventional C=O···H-C hydrogen bonds only between adjacent residues. Similar reasons led to the exclusion of oligoproline derivatives [48] and collagen-model peptides, as shown in Figure 3f [49].

It is very important to mention peptoids, as shown in Figure 1e, which have been reported here only as representative examples. They perfectly fall into the Moore’s definition, because both their helices, having about three residues per turn, and their extended conformations are stabilized by many intrastrand non-covalent bonds [50,51,52,53,54,55] (e.g., van der Waals, hydrogen bonding and π–π interactions between side chains of non-adjacent residues), and they can also form loops [56] and turns [57]. Their different self-assemblies have been extensively and rigorously treated in a huge number of works, the main contribution being due to Zuckermann’s group. However, up to the present, this impressive amount of literature has been periodically and continuously reviewed in all aspects, even as far as biophysical techniques are concerned, so that a further treatment in this context would have been out of the scope of this review. Thus, beyond the examples reported in Section 4, the interested reader can find an extremely complete and up to date coverage of peptoid self-assembly in many recent reviews and book chapters dealing (partially or entirely) with this topic [58,59,60,61,62,63,64,65,66,67,68,69,70,71], including two reviews published during the writing of the present manuscript [72,73].

As a last general observation, self-assembly that occurs only when other species are added to the solution (e.g., metal- [74,75], halogen- [76,77,78], and ion-pair-bonding-directed [79] self-assembly of aromatic foldamers in double helices or other duplexes, as well as fiber formation triggered by metals [80] or fullerenes [81]; metal-directed self-assembly of peptoid foldamers [82]) and the self-assembly of nanostructures mediated by foldamers (e.g., nanoparticle formation mediated by peptoid foldamers [83]), will not be treated within this review.

## 2. β-Peptide Foldamers

β-peptides are the most well-known foldamers based on a peptidic backbone. Formally, they can be seen as the smallest structural variation with respect to α-peptides, due to the presence of an additional rotatable bond. However, the rational exploitation of their much greater number of possible substitution schemes, with different absolute and relative stereochemistries, and conformational restrictions (e.g., incorporations of α and β carbons in cycles) still leads to an enhanced ability to form a large number of predictable and sometimes inherently very stable secondary structures [1,5,7,8].

### 2.1. Discrete Self-Assembly in Solution: Up to Bundles

#### 2.1.1. Nucleobase-Guided Pairing in Aqueous Solution

**Figure 4:**
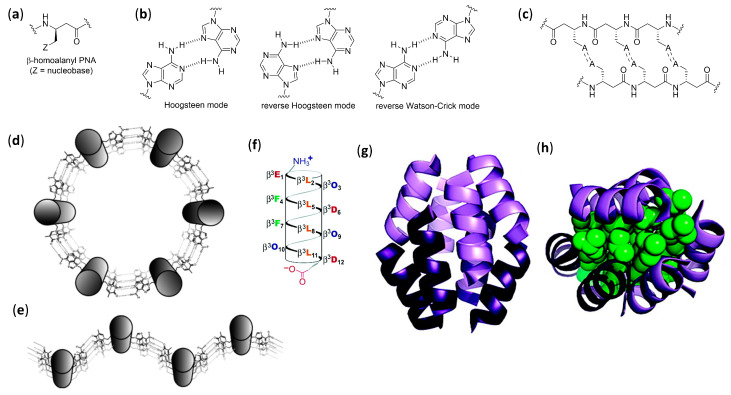
(**a**) A generic β-homoalanyl PNA (Refs. [84,85]); (**b**) Possible A-A pairing modes (Refs. [84,85]); (**c**) Beta-sheet-like antiparallel double strand structure of a generic adeninyl β-homoalanine foldamer (Refs. [84,85]); (**d**) Cyclic and (**e**) band-like modes of aggregation of two-side nucleobase-functionalized β-peptide 14-helices (helices reported as cylinders) (adapted with permission from Ref. [91]); (**f**) Cylinder representation of foldamer Zwit-1F (β^3^-amino acids indicated using the single-letter codes of the corresponding α-amino acids. β^3^O = β^3^-homoornithine) (adapted with permission from Ref. [96]); (**g**) Ribbon representation of octameric bundle of foldamer Zwit-1F, together with (**h**) its tightly packed hydrophobic core (β^3^-homoleucine side chains are shown in space-filling representation) (adapted with permission from Ref. [96]).

Mainly due to the efforts of Diederichsen’s group, β-peptide foldamers have been demonstrated to self-assemble in duplexes, or even higher-order structures in aqueous solution by exploiting the recognition abilities of nucleobases. When β-homoalanyl peptide nucleic acids (β-homoalanyl PNAs), uniformly functionalized—with adenine, for example, depicted by Z = A in Figure 4a—were employed, the low propensity of the non-preorganized acyclic monomers to the formation of the 14-helix in water was easily overcome by the stabilization offered by the extended π-stacking of nucleobases and by the possibility to form the largest possible number of pairings in linear strands. In these cases, for very short oligomers (tetramer) or when the Watson–Crick pairing sites were the only ones available (e.g., as in 7-carbaadenine-β-PNA derivatives), the expected β-sheet-like double strands were observed, due to self-pairing with the reverse Watson–Crick mode, as shown in Figure 4b,c. The stability of the longer oligomers was extraordinary, as evidenced by measuring the UV hyperchromicity (i.e., the increase in absorbance due to unpairing) and the CD-derived melting points. On the other hand, for longer adenine-functionalized oligomers, the possibility of a completely different hydrogen bonding pattern due to the alternative Hoogsteen pairing sites in the nucleobase led to unsafely identified higher-order self-assemblies [84,85].

When the nucleobases were inserted, all three residues in β-peptides containing *trans*-2-aminocyclohexanecarboxylic acid (*trans*-ACHC)—a cyclic β-amino acid, as shown in Figure 1c,—that strongly favor the 14-helix folding [86,87] and the formation of linear strands, were avoided and the expected Watson–Crick pairing was observed between the nucleobase-functionalized sides of two 14-helices. Exploiting, again, UV hyperchromicity and CD spectra, the formation of either highly stable duplexes (mainly for oligomers functionalized with self-complementary sequences (e.g., TATA)) or heteroduplexes (for equimolar mixtures of two oligomers functionalized with complementary sequences) in aqueous media was demonstrated [88,89,90]. When β-peptide 14-helices were functionalized on two sides of the helix with the suitable nucleobase sequences, higher aggregations could be detected by UV and CD, even though the exact arrangements were not determined, as shown in Figure 4d,e [91].

#### 2.1.2. Bundles in Aqueous Solution

The first step toward unnatural helix-bundle quaternary structure in aqueous solution was due to the Gellman’s group, which examined the behavior of two different decameric β-peptides with high propensity to form 14-helices, due to six residues of *trans*-ACHC, and also containing three positively charged residues of β^3^-homolysine [92]. The sedimentation equilibrium analytical ultracentrifugation (SE-AU) and the concentration-dependent broadening of ^1^H NMR signals allowed us to detect the possible self-assembly in aqueous buffered solutions. While the nonamphiphilic (scrambled) foldamer showed no aggregation, the amphiphilic 14-helix of β^3^-hTyr-(ACHC-ACHC-β^3^-hLys)_3_NH_2_ formed soluble tetrameric-hexameric bundles, which very likely had the cationic sides oriented toward the aqueous solution, and a solvent-excluded hydrophobic core made of nonpolar helical faces pointing to each other. In a further theoretical work, the electrostatic interactions of terminal groups and the helix macrodipole of the Gellman’s amphiphilic decamer were shown to be more important than Lennard–Jones interactions for the head-to-tail assembly in water, contrary to similar cases for α-peptides self-assembling in a side-by-side fashion [93]. Interestingly, the self-assembly in bundles was found to be critical for the N-terminal acylated version of the same decamer, heptanoyl-β^3^-hTyr-(ACHC-ACHC-β^3^-hLys)_3_NH_2_, which was able to catalyze the retroaldol reaction of the β-hydroxyketone 4-phenyl-4-hydroxy-2-oxobutyrate to give benzaldehyde and pyruvate in an aqueous environment [94].

Schepartz et al. also demonstrated that dodecapeptides built using only acyclic β^3^-homoamino acids could form water-soluble bundles of 14-helices when the hydrophobic core was constituted by the helix sides containing β^3^-homoleucine, and another side in every helix was functionalized alternately with β^3^-homoornitine and β^3^-homoaspartic acid, thus forming a net of intra- and interhelical salt-bridges. According to sedimentation equilibrium studies (analytical ultracentrifugation), and composition- and temperature-dependent CD spectra, a salt-bridged hetero-hexamer or octamer containing a 1:1 ratio of the two helices was deduced at pH 7.1 [95]. Using the same CD spectroscopies, and also X-ray crystallography, for a strictly related zwitterionic β-dodecapeptide (Zwit-1F), as shown in Figure 4f, the authors could verify the formation of a complex octameric bundle with a highly symmetrical disposition of parallel and antiparallel helical pairs. Both in the crystal and in aqueous solution at pH 7.1, the pairs were disposed in a hand-like (or palm-to-palm) manner, leaving all the lipophilic side chains tightly packed in the hydrophobic core, as shown in Figure 4g,h [96]. A further improvement in bundle stability was obtained through very limited changes within the twelve β-amino acids, which generated additional intermolecular hydrogen bonds, as witnessed by differential scanning calorimetry (DSC), SE-AU, and CD data [97]. The same authors demonstrated that the octameric bundle could tolerate the substitution of a suitable β^3^-homoleucine with its hexafluoro derivative, packing all the CF_3_ groups in a fluorous subcore [98].

Rationally designed Schepartz’s β-dodecapeptide octameric bundles were also demonstrated to be able to mimic the action of proteins. Specifically constructed versions of the thermodynamically stable β^3^-peptidic octameric bundles were shown to possess the capability to bind and hydrolyze the ester function of a water-soluble 8-acetoxypyrene derivative [99]. Single-crystal X-ray analysis showed that the rationale for catalytic activity was the presence of many potential active sites (20 per bundle), whose catalytic triads were composed of two β^3^-homoarginines and an α-histidine tactically placed as the N- and/or C-terminal residue. In addition, the chiral bundles were able to catalyze the hydrolysis of (*R*)-2-phenylpropionate pyrene ester with a moderate level of chiral discrimination. Further, the authors used SE-AU, CD and X-ray crystallography to verify that changing the phenolic moiety of β^3^-homotyrosine (β^3^-hTyr) residues on the surface of suitable octameric bundles with phenyl boronic acid (PBA), by using 4-borono-β^3^-homophenylalanine instead of β^3^-hTyr [100]. Only in one case did this not affect the palm-to-palm quaternary structure already reported [96,97]. The PBA-derivatized octameric bundle was able to bind different polyols in aqueous buffer solution with higher affinity than a constitutively monomeric 14-helix PBA-modified analogue. When a neutral β^3^-hTyr of this borono-bundle was changed for a charged β^3^-homoornitine (β^3^-hOrn), the modified octameric bundle bound four sorbitol molecules with an affinity increase in the range of two orders of magnitude with respect to the β^3^-hTyr parent foldamer, due to the more positive electrostatic potential in binding sites [101].

#### 2.1.3. Dimers and Bundles in Methanol

The axial helix–helix interactions were evidenced to be essential for the self-dimerization-driven formation of the very peculiar large-diameter mixed 18/20-helix of an heptamer made of *cis*-2-aminocyclopentanecarboxylic acid (*cis*-ACPC) in methanol [102]. Bundles with a variable aggregation number and characterized by side-by-side interchain interactions, instead of head-to-tail, could be observed by Fülöp et al. for short *trans*-ACHC oligomers in methanol by means of CD and DOSY (diffusion-ordered spectroscopy) NMR experiments, the main driving force being, in this case, the hydrophobic interactions among the apolar side chains [103].

#### 2.1.4. Ion Channels in Phospholipid Bilayers

A last example of a discrete number of self-assembling β-peptides that can mimic the action of the α-peptide natural counterparts was described for cyclic β^3^-tetrapeptides [104]. Analogous to the previously reported formation of peptide nanotubes, based on hydrogen bonded stacked arrangements of *cyclo*-D,L-α-peptides [105], Ghadiri et al. synthesized *cyclo*[(-β^3^-hTrp)_4_-] and *cyclo*[(-β^3^-hTrp-β^3^-hLeu)_2_-] as proper foldamer units to self-assemble within phospholipid bilayers. The artificial transmembrane ion channels were formed in large unilamellar vesicles by the association of both *cyclo*-β^3^-tetrapeptides, as determined by FT-IR (Fourier transform infrared spectroscopy), and the measure of either carboxyfluorescein fluorescence or single channel conductance showed the high transport activity for H^+^ or K^+^ ions, respectively. The assembly–disassembly dynamic equilibrium was assessed, together with the concomitant possibility for opening, closing and collapse events, as well as variation in the number of foldamers involved in the channel structure.

### 2.2. Extended Self-Assembly: Vesicles and Fibers

#### 2.2.1. Vesicles in Protic Solvents

**Figure 5:**
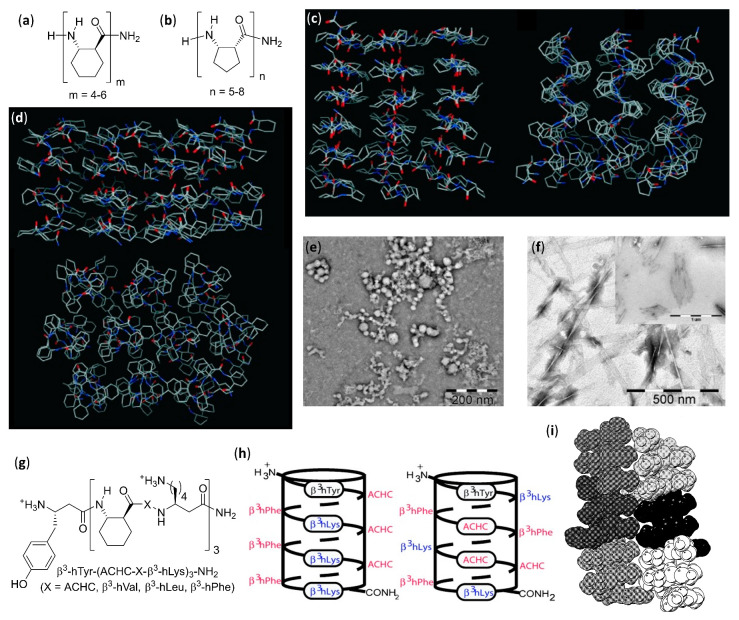
(**a**) H-(*trans*-ACHC)_m_-NH_2_ foldamers (Refs. [8,106]); (**b**) H-(*cis*-ACPC)_n_-NH_2_ foldamers (Refs. [8,106]); (**c**) Side (left) and top (right) views of a fibril fragment of H-(*cis*-ACPC)_7_-NH_2_ after 3 ns MD simulation (hydrogens omitted for clarity); (**d**) Top (upper) and side (lower) views of helix–bundle membrane fragment of H-(*trans*-ACHC)_6_-NH_2_ after 3 ns MD simulation (hydrogens omitted for clarity); (**e**) TEM image of H-(*trans*-ACHC)_4_-NH_2_ vesicles after 1 day (1 mM in MeOH); (**f**) TEM image of H-(*cis*-ACPC)_7_-NH_2_ fibrils after 1 week (1 mM in MeOH) (inset: fibrils and sheets measured in water) (panels c–f adapted with permission from Ref. [106]); (**g**) β^3^-hTyr-(ACHC-X-β^3^-hLys)_3_-NH_2_ 14-helix foldamers (X = ACHC, β^3^-hVal, β^3^-hLeu, or β^3^-hPhe, Refs. [111,112,113]); (**h**) Cylinder representations of 14-helices generated by the globally amphiphilic β^3^-hTyr-(ACHC-β^3^-hPhe-β^3^-hLys)_3_-NH_2_ (left) and its non-amphiphilic isomer β^3^-hTyr-β^3^-hLys-β^3^-hPhe-ACHC-β^3^-hPhe-β^3^-hLys-ACHC-ACHC-β^3^-hPhe-β^3^-hLys-NH_2_ (adapted with permission from Ref. [111]); (**i**) Space-filling view of six molecules of Boc-ACHC_6_-OBn in the crystal structure, highlighting the “cyclohexyl zipper” (all six molecules have the same orientation with C-termini at the bottom) (reprinted with permission from Ref. [118]).

The controlled self-assembly of β-peptide foldamers, inducing either the formation of helix bundles or pleated sheets, was obtained by Fülöp’s group by accurate choice of structure and configuration of β-peptide backbone, and ultimately led to the formation of vesicles or fibrils, respectively [106]. In both cases, transmission electron microscopy (TEM), dynamic light scattering (DLS), and diffusion-ordered spectroscopy (DOSY) NMR experiments were used to follow the hierarchical superstructure formation from the initial stages to the final morphologies, also with the aid of molecular dynamics simulations. The self-assembly of the vertically amphiphilic 14-helices of N-terminal unprotected *trans*-ACHC oligomers, as shown in Figure 5a, with carboxamide C-terminal functions in methanol or water led to the solvent-driven formation of multilamellar vesicles, as shown in Figure 5e.

In every unilamellar wall, the thickness was equal to the length of two helices, assembled either in a head-to-head or head-to-tail fashion and, in these bilayers, the 14-helices were tightly packed by the hydrophobic interactions among the lipophilic cyclohexyl side chains, as shown in Figure 5d [106]. This hydrophobically-driven self-assembly was also noted for the related hexamer made of *cis*-ACHC units with alternating absolute configurations that, in spite of its conformational polymorphism between the major 10/12-helix and a minor differently folded secondary structure, furnished vesicles in water [107]. Moreover, the comparison of hydrophilic/hydrophobic surface ratios among the vesicle-forming heterochiral *cis*-ACHC hexamer and other oligomers under study evidenced that only the foldamer with the highest ratio (i.e., the *cis*-ACHC hexamer itself) led to self-assembly. The same group further described oligomers of *cis*-1-amino-1,2,3,4-tetrahydronaphthalene-2-carboxylic acid (*cis*-ATENAC) with alternating absolute configurations, having, again, a free amine at the N-terminus and a carboxamide at the C-terminus [108]. The extra stabilization, due to CH–π interactions between the backbone hydrogens on alpha carbons and the aromatic rings of side chains, led to the formation of the peculiar 10/12-helix in methanol. The protonation of N-termini generated vertically amphiphilic secondary structures with highly hydrophobic helix surfaces, analogously to the previously studied 14-helices of *trans*-ACHC [106]. In methanol, it could be possible to observe vesicles whose sizes were correlated to the hydrophobic surface area, and then to the number of monomeric units bearing the lipophilic aromatic rings.

#### 2.2.2. Fibers in Methanol

Using methanolic solutions of homochiral *cis*-ACPC oligomers in the N-terminal unprotected form, as shown in Figure 5b, which had already been known to form nonpolar β-strand-like secondary structures [109], the same group obtained fibrils showing random helical twists, as shown in Figure 5f [106]. The fibril lengths were in the micrometer range, and the heights of 2–3 nm—corresponding to the lengths of single chains of *cis*-ACPC oligomers—confirmed the supposed side-by-side aggregation, as shown in Figure 5c. Size and morphology were also demonstrated to be solvent- and time-dependent, and the fibril stability was attributed to the high ζ potential, which in turn was generated by the (partly) protonated N-terminal amine functions on the surface.

#### 2.2.3. Fibers in Aqueous Solution

The dissolution in water of amphiphilic β-peptide foldamers β^3^-hTyr-(ACHC-ACHC-β^3^-hLys)_n_-NH_2_ with a high content of the 14-helix-inducing *trans*-ACHC β-amino acid and a different number of repeating triads, led to lyotropic liquid crystals (LLC), made of hydrogelating nanofibers with lengths in the micrometer range, were generated. This was shown by the observation of birefringence with an optical microscope (polarized optical microscopy, POM) and confirmed by the splitting of the D_2_O signal in variable temperature ^2^H NMR experiments [110]. The self-assembly was favored for longer oligomers, while a non-globally amphiphilic version of the best fiber-forming oligomer (n = 3) could not show any aggregation, even at the highest concentrations tested.

A further series of experiments conducted by Gellman et al., using isomeric foldamers that were either globally or non-globally amphiphilic and also incorporated reasoned mutations in side chains, allowed us to draw interesting conclusions [111,112,113]. Even if global amphiphilicity was previously suggested to favor liquid crystallinity [110], it was further demonstrated that it was not a conditio sine qua non. In fact, the globally amphiphilic foldamer β^3^-hTyr-(ACHC-β^3^-hPhe-β^3^-hLys)_3_-NH_2_, as shown in Figure 5g (X = β^3^-hPhe) only formed globular aggregates, whereas its non-amphiphilic isomer, β^3^-hTyr-β^3^-hLys-β^3^-hPhe-ACHC-β^3^-hPhe-β^3^-hLys-ACHC-ACHC-β^3^-hPhe-β^3^-hLys-NH_2_, which had a scrambled distribution of β^3^-homolysine (β^3^-hLys) residues, as shown in Figure 5h, formed a LC phase in aqueous solution [111]. By means of cryogenic transmission electron microscopy (cryo-TEM) and an improved fit to the small angle X-ray scattering (SAXS) data, it was further demonstrated that the high-aspect-ratio LC phase-forming fibers of the non-globally amphiphilic isomer had a hollow core [114]. A high hydrophobicity was also shown not to be sufficient to guarantee LLC formation in an aqueous environment within this family of foldamers, but the presence of aromatic chains was crucial, as verified by changing the phenyl rings of β^3^-homophenylalanine (β^3^-hPhe) residues in the non-amphiphilic isomer with more hydrophobic cyclohexyl rings, which prevented LC formation. Eventually, the reduction in electrostatic repulsion between adjacent foldamer molecules, obtained by substituting β^3^-hLys in position 6 of the non-amphiphilic isomer with a negatively charged (β^3^-hGlu) or neutral residue (β^3^-hGln), enhanced liquid crystal phase formation by decreasing the overall net charge [111].

In a subsequent study, various other *trans*-ACHC-rich amphiphilic 14-helix decamers with a differently derivatized N-terminal β^3^-homotyrosine (β^3^-hTyr) and ACHC-X-β^3^-hLys repeating triads (X = *trans*-ACHC, β^3^-hVal, β^3^-hLeu, or β^3^-hPhe) were evaluated for their ability to act as mesogens, and thus to generate LC phases containing high-aspect-ratio nanofibers in water [113]. To obtain a complete description of the morphologies generated and the processes involved, many different experimental tools (i.e., SAXS, cryo-TEM, POM, CD and ^2^H NMR) were used. As the main driving force for the self-assembly of this class of foldamers in nanofibers, the authors proposed the interaction among the hydrophobic surfaces of interdigitating cyclohexyl side chains in adjacent β-peptide molecules. This so-called “cyclohexyl zipper”, related to the “leucine zipper” commonly found in α-peptides that hydrophobically self-assemble in coiled-coils [115,116], was crystallographically evidenced for the foldamer Boc-ACHC_6_-OBn, as shown in Figure 5i [117,118]. As an additional observation, the global amphiphilicity and the “cyclohexyl zipper” interactions of both the β-peptide decamer β^3^-hTyr-(ACHC-ACHC-β^3^-hLys)_3_-NH_2_ and its N-terminal-thiolated derivative were ascertained to be essential for their ordered self-assembly in monolayers on gold [119].

**Figure 6:**
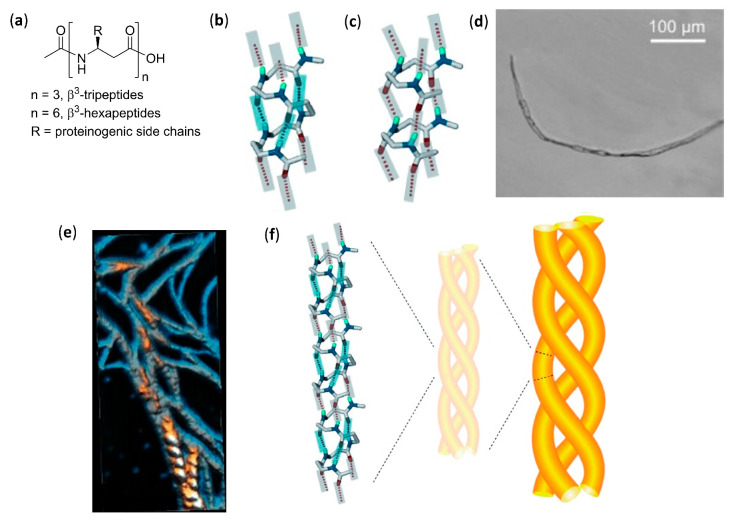
(**a**) Representative structures of β^3^-tripeptides (n = 3) and β^3^-hexapeptides (n = 6) (Refs. [120,121,122,128]); Intermolecular (grey) and intramolecular (cyan) hydrogen bonds in the crystal structures of (**b**) a β^3^-hexapeptide, and (**c**) a β^3^-tripeptide (side chains and unnecessary hydrogens omitted for clarity) (adapted with permission from Ref. [120]); (**d**) Microscopic fiber formed by the β^3^-tripeptide Ac-β^3^-hTrp-β^3^-hSer-β^3^-hIle-OH (adapted with permission from Ref. [120]); (**e**) 3D rendering of an AFM image of fibrils in a microscopic fiber of β^3^-tripeptide Ac-β^3^-hTrp-β^3^-hSer-β^3^-hIle-OH, highlighting the periodicity in fibrils (adapted with permission from Ref. [120]); (**f**) Supposed “self-twining” process at the basis of the higher-order hierarchical self-assembly from nanorods to individual fibrils, and then to fibers (adapted with permission from Ref. [120]).

An extensive effort in the hierarchical self-assembly of β-peptides, directed to the formation of nanofibers that could also form macroscopic fibers, was done by Aguilar’s and Mechler’s groups [120,121,122,123,124,125,126,127,128,129,130]. In a seminal work, they rapidly obtained both microscopic and macroscopic fibers by simply dissolving various N-terminal acetylated β^3^-tri- and β^3^-hexapeptides in methanol or water, as shown in Figure 6a. While the microscopic fibers were analyzed by scanning electron (SEM) and atomic force (AFM) microscopies, as shown in Figure 6d,e, the macroscopic fibers had lengths ranging from few millimeters to three centimeters and were visible to the naked eye [120]. The most important variables to trigger the hierarchical self-assembly of nanorods, intertwining fibrils and eventually macroscopic fibers, were identified. The authors used a number of monomers equal to a multiple of three, which is the number of residues per turn of 14-helices. This allowed the theoretical possibility to either form secondary structures stabilized by three intramolecular hydrogen bonds (for β^3^-hexapeptides), as shown in Figure 6b, or still arrange into the same folding, even when the intramolecular hydrogen bonding pattern of 14-helix could not be formed (for short β^3^-tripeptides), as shown in Figure 6c. Single-crystal X-ray data confirmed this assumption and also highlighted that side chains of different peptide units were perfectly aligned along the nanorods, as shown in Figure 6f. Remarkably, no fiber formation was evidenced when the corresponding N-terminal free amines were dissolved in water or methanol [120]. The hierarchical self-assembly was demonstrated to follow a multistep mechanism: the smallest structures generated from nanorods were helical nanofibrils formed by three to five intertwined nanorods, as determined by the diameter and surface periodicity, and then the further “self-twining” process of these fibrils led to larger fibers of increasing size, again evidencing a surface periodicity, as shown in Figure 6d–f. However, the preferred intermolecular interactions among single nanorods in a nanofibril, and also among single nanofibrils in a fiber, were not determined.

In a following series of experiments, the same authors studied, by AFM and far-IR spectroscopy, the self-assembly in water of isomeric N-acetylated-β^3^-tripeptides with different sequences of β^3^-hLeu, β^3^-hIle, and β^3^-hAla [121]. They found that, in spite of the similarity among the different nanofibrils, the lateral topology of nanorods had a deep impact on the superstructures formed. In particular, the strength of lateral interactions among side chains resulted to be crucial for the interfibril self-assembly and then for the final morphology observed, whereas the C-terminal carboxylic acid did not contribute to the interfibril interactions because of solvation [121].

The solvent effects were better studied by AFM and optical microscopy investigation of the different superstructure morphologies of tripeptide Ac-β^3^[LIA] (Ac-β^3^-hLeu-β^3^-hIle-β^3^-hAla-OH) [122]. Acetone and chloroform favored the hydrogen bonding interactions among non-solvent-exposed C-terminal carboxyl residues, which in turn favored their interfibril interactions and generated straight fibers. In alcohols, the solvation of C-terminal carboxyl residues made the delicate balance of solvophobic and van der Waals interactions more important, leading to various dendritic self-assemblies. In water, the solvophobic effects predominated, thus favoring the internal segregation of lipophilic side chains in a cylindrical twisted arrangement and obtaining hierarchically twisted structures reminiscent of a rope [122].

The individual fibrils obtained from the dissolution of Ac-β^3^[LIA] tripeptide (i.e., coiled-coil triple helices) and its sequence isomers in water, alcohols, and dimethylsulfoxide were first observed by electron and atomic force microscopies. Then, precise foldameric arrangements in fibrils were elegantly deduced from X-ray fiber diffraction data, followed by molecular dynamics simulations [128]. Importantly, it was further demonstrated that, albeit the rational design based on β^3^-peptides containing 3×n (n = integer number) β^3^-amino acids was easy to implement, β^3^-tetrapeptides could also be used. Obviously, in this case, the 14-helical arrangement of each nanorod-forming β^3^-tetrapeptide completed 1 1/3 turns of the helix [126].

**Figure 7:**
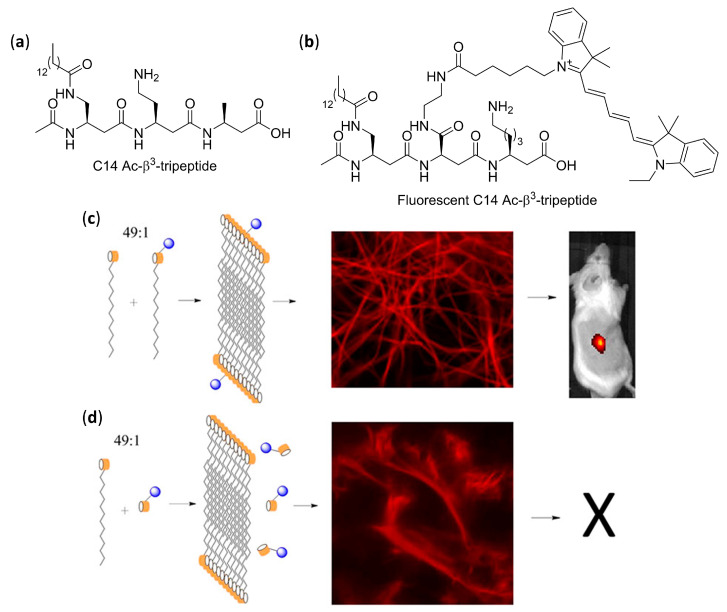
(**a**) The lipidated C14 Ac-β^3^-tripeptide (Refs. [123,125,130]) and (**b**) its fluorescent analogue (Ref. [129]); (**c**) From left to right: schematic representations of fibers formation by 49:1 mixtures of lipidated C14 Ac-β^3^-tripeptide and its fluorescent analogue; confocal image of the corresponding hydrogel showing successful incorporation of the fluorophore into the fiber matrix; in vivo animal imaging 14 days post-injection (ochre cylinders: Ac-β^3^-tripeptide skeleton; zigzag chains: C14 acyl chain; blue spheres: Quasar^®^ 670 emitting dye) (reprinted with permission from Ref. [129]); (**d**) The fluorophore was not incorporated into the fiber matrix when a 49:1 mixture with the non-lipidated version of fluorescent Ac-β^3^-tripeptide was used (reprinted with permission from Ref. [129]).

AFM and TEM analyses allowed us to observed the uniformly sized fibrils made of just two intertwined nanorods, having a head-to-tail hydrogen bonding pattern of pairing foldamers, obtained by dissolving in aqueous environment β^3^-tripeptides functionalized with cell-adhesion signals (IKVAV, RGD, or both α-peptide sequences) on the second residues [124]. They were demonstrated to be functional bio scaffolds, able to exhibit cell-specific modulation and altered cellular responses. Bioactivity was also obtained with fibers self-assembled from a α/β hybrid made of two β^3^-tripeptides β-hSer-β-hVal-β-hAla grafted by the RGD α-peptide [127]. In the related search for β^3^-peptides capable of forming nanofibers networks that could entrap water and form hydrogels, Aguilar’s group synthesized a stable and reversible hydrogel by dissolving an Ac-β^3^-tripeptide bearing a C14 acyl chain on the first residue (Ac-β-hAla(C_14_)-β-hLys-β-hAla-OH), as shown in Figure 7a [123].

According to fluorescence microscopy, SN4741 neuronal progenitor cells deposited on top of the hydrogel showed high cell viability and spreading, but only when serum was added to the culture medium, due to the fact that no cell signaling was incorporated in the β-peptidic scaffold. A long-lasting injectable hydrogel obtained from a 90:10 mixture of C14 Ac-β^3^-tripeptide and an RGD-modified β^3^-tripeptide (Ac-β-hAla(C_14_)-β-hAla(RGD)-β-hLys-OH) in phosphate buffered saline (PBS) was then implanted into transgenic mice [130], showing by fluorescence microscopy the ability to both divert neural stem cells (NSCs) from the subventricular zone to the cortex and differentiate NCSs into neurons and astrocytes. Interestingly, the experiments also suggested a possible integration of neurons.

Moreover, when a 49:1 mixture in PBS of lipidated C14 Ac-β^3^-tripeptide and a lipidated analogue bearing the Quasar^®^ 670 emitting dye was used, as shown in Figure 7a,b, confocal fluorescence microscopy and stimulated emission depletion microscopy (STED), a technique of super-resolution imaging, confirmed the homogeneous incorporation of fluorophore tripeptide into fibers. The resulting fibrous hydrogel could be subcutaneously injected in conscious mice, and high-intensity persistent fluorescence was still measured by in vivo imaging after 14 days, as shown in Figure 7c [129]. On the contrary, the same experiment failed when the non-lipidated version of the fluorescent β^3^-tripeptide was used together with the C14 Ac-β^3^-tripeptide, as shown in Figure 7d.

### 2.3. Self-Assembly into Solid Tridimensional Structures in Aqueous Environment

The greatest efforts in the field of β-peptide with well-defined and amazing solid tridimensional (3D) structures, different from the simpler morphologies described in the previous subsection, have been due to Lee’s group, who coined the term “foldecture” (from foldamer and architecture) [131,132,133,134,135,136,137]. Foldectures are actually self-assembled crystalline solids formed in nonequilibrium conditions, and the molecular packing can be either quite similar or very different from that obtained in single crystals grown in equilibrium conditions. In all of these recent works, they used the predictable, stable and rigid 12-helix structure formed in the crystal and in solution by homooligomers of *trans*-2-aminocyclopentanecarboxylic acid (*trans*-ACPC) [138], in both its *S*,*S* and *R*,*R* absolute stereochemistries, and analyzed the 3D structures mainly using SEM. The authors were able to identify the variable governing the aqueous self-assembly and obtain different morphologies by apparently small changes in either the foldamer primary structure or the type and concentration of surfactant.

**Figure 8:**
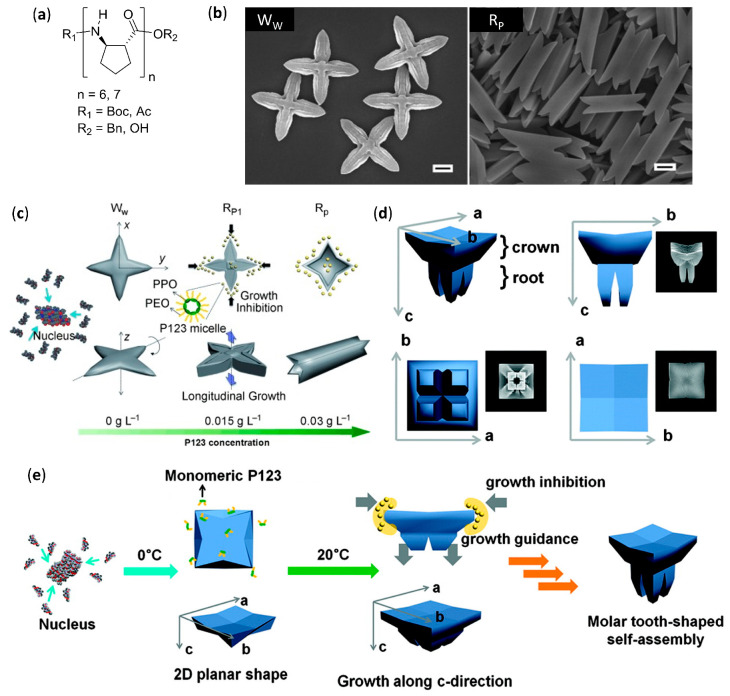
(**a**) General structure of *trans*-ACPC oligomers investigated (both *S*,*S* and *R*,*R* absolute stereochemistries were employed) (Refs. [131,132,133,134,135,136,137,138]); (**b**) SEM images of structures self-assembled from Boc-ACPC_7_-OBn in distilled water (W_W_) and in a 0.03 g L^−1^ aqueous solution of P123 (R_P_) (scale bars: 1 µm, adapted with permission from Ref. [131]); (**c**) Rationale for the surfactant-based changes in the self-assembled structures of Boc-ACPC_7_-OBn in distilled water and in an aqueous solution of surfactant P123: after the initial fast nucleation, the growth in pure water is faster along the x and y axes, due to the presence of the most hydrophobic foldamer moieties, thus favoring tip elongation and creating the windmill-shaped (W_W_) morphologies. In the presence of high concentration of P123 micelles, which solvate mainly the most hydrophobic surfaces, the contrary occurs, obtaining the R_P_ morphology (adapted with permission from Ref. [131]); (**d**) 3D schematic representations and corresponding SEM images, as viewed along different axes, of the molar tooth-shaped (M_P_) self-assembled structures of Boc-ACPC_6_-OBn in a concentrated aqueous solution (8 g L^−1^) of P123 (reprinted with permission from Ref. [132]); (**e**) Formation of the molar tooth-shaped structures of Boc-ACPC)_6_-OBn in a concentrated aqueous solution of P123: a four-fold dendritic flat structure forms at 0 °C, when P123 is below its critical micellization temperature, but, upon increasing temperature to 20 °C, the differential solvation, due to P123 micelles (yellow), favors a faster growth along the *c* direction and the formation of the root (reprinted with permission from Ref. [132]).

The addition of the highly hydrophobic heptamer of *trans*-ACPC Boc-ACPC_7_-OBn, as shown in Figure 8a (n = 7) dissolved in tetrahydrofuran (THF) to distilled water led to the immediate precipitation of a solid with size in the micrometer range and a windmill-shaped morphology, as shown by W_W_ in Figure 8b [131]. On the other hand, when the foldamer was added to an aqueous solution of the nonionic surfactant—(ethylene glycol)_20_-(propylene glycol)_70_-(ethylene glycol)_20_ (Pluronic P123, from 0.03 g L^−1^ to 8 g L^−1^)—square rods were isolated, whose tapered faces were evidently reminiscent of the 90° disposition of the sails in the W_W_ shape, as shown by R_P_ in Figure 8b.

**Figure 9:**
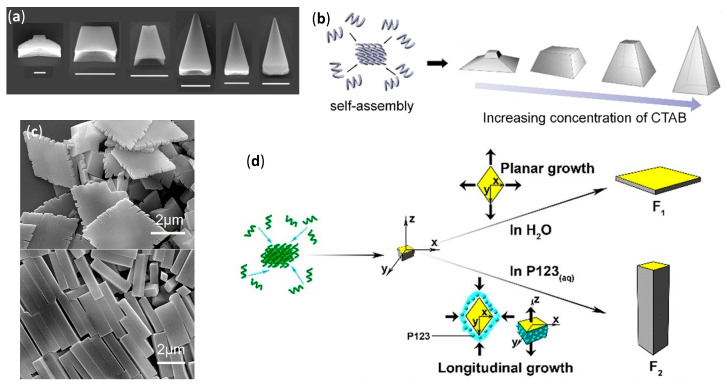
(**a**) SEM images of morphologies obtained from Boc-ACPC_6_-OBn in (from left to right) 0.05, 0.2, 0.3, 2, 5, and 10 g L^−1^ aqueous CTAB solutions (scale bars: 1 µm, adapted with permission from Ref. [137]); (**b**) Relationship between 3D shape of a foldecture and increasing concentration of CTAB (reprinted with permission from Ref. [137]); (**c**) SEM images of the self-assembled structures of Boc-ACPC_6_-OH from distilled water (F_1_, upper) and aqueous P123 solution 8 g L^−1^ (F_2_, lower) (adapted with permission from Ref. [135]); (**d**) Rationale for the surfactant-based changes in the self-assembled structures of Boc-ACPC_6_-OH in distilled water and in an aqueous P123 solution. In water, the initially formed rhombic structure grows faster on the most hydrophobic facets (in gray, x-y plane) by successive addition of hydrophobically packed foldamer molecules, thus generating rhombic plates (F_1_), while in the presence of P123 micelles, the preferential solvation of the hydrophobic facets favors the growth along the z axis (hydrophilic facets, in yellow), forming the rhombic rods (F_2_) (reprinted with permission from Ref. [135]).

A stepwise change from the two morphologies was experimentally correlated to P123 concentration, as shown in Figure 8c, and especially to the critical micellar concentration (0.015 g L^−1^), above which the elongation became highly dominant. Basically, in pure water the hydrophobic and head-to-tail hydrogen bonding interactions were at the basis of windmill formation and subsequent growth. However, in the presence of P123, its micelles differentially solvated the windmill at an early stage, thus greatly decreasing the growth in two directions (x and y) in favor of the longitudinal growth, as depicted by z in Figure 8c. In a further study, the addition of an oligomer shortened by one unit, the hexamer Boc-ACPC_6_-OBn, as shown in Figure 8a (n = 6), to a 8 g L^−1^ aqueous solution of P123 led to the very peculiar molar tooth-shaped (M_P_) morphology, as shown in Figure 8d [132]. The resulting self-assembled structure was again the result of a differential solvation by means of P123 micelles, as ascertained by changing the experimental temperatures above or below the critical micellar temperature, as shown in Figure 8e. The preferential adsorption of a different (cationic) surfactant, namely cetyltrimethylammonium bromide (CTAB), onto the different facets of the growing self-assembled structure of the same β-hexapeptide, Boc-ACPC_6_-OBn, was further studied [137]. Powder X-ray diffraction (PXRD) data of the different morphologies obtained, as shown in Figure 9a, highlighted that all these Boc-ACPC_6_-OBn-based foldectures, as well as those obtained using P123 as surfactant, basically had the same molecular packing, characterized by extremely similar intra and intermolecular hydrogen bonds and hydrophobic interactions among the foldameric units. Even in the case of CTAB, it was the preferential adsorption of the surfactant molecules on two crystal faces that drove the formation of different final shapes, and the increase in CTAB concentration deviated the 3D structure from a square plate to a square pyramid, as shown in Figure 9a,b.

When the hexamer with a C-terminal acid, Boc-ACPC_6_-OH, was subjected to self-assembly in water and aqueous P123 solution (8 g L^−1^), rhombic plates (F_1_) and rhombic rods (F_2_) were, respectively, obtained, whose facets had identical internal angles, as shown in Figure 9c [135]. The molecular PXRD-derived molecular packings of both shapes were almost identical, being driven by the same head-to-tail intermolecular hydrogen bonds and lateral hydrophobic interactions. Then, rods were again formed by virtue of a differential surfactant adsorption onto nascent shapes, as corroborated by molecular dynamics simulations. The interaction of surfactant with the lateral facets of nascent rhombic plates, exposing the apolar side chains, were favored and led to the consequent preferential longitudinal growth, as shown in Figure 9d. The authors further demonstrated, by high-resolution optical microscopy, that the rhombic rods of Boc-ACPC_6_-OH had sufficient total diamagnetic anisotropy to instantaneously align in presence of an external magnetic field, and this collective motion could also be applied to a macroscopic hydrogel container [136].

Interestingly, SEM and TEM images evidenced that, when the C-terminal benzylated hexamer Boc-ACPC_6_-OBn previously used was dissolved in a 8:2 methanol/water mixture, the slow evaporation of solvents from a silicon surface left chiral hollow parallelepipeds with a stair-like layered interior divided by a diagonal wall [133]. The change in solvent system and methodology with respect to the previous experiments [132] did not simply drive a different self-assembly by a distinct preferential solvation after the same initial nucleation, but actually generated a more intimate change in crystallinity itself, as ascertained by PXRD, thermogravimetric analysis, and differential scanning calorimetry. The shape was then attributed to the similarity of evaporation-induced self-assembly of Boc-ACPC_6_-OBn with the diffusion-limited crystallization, in which the growth rate at the edge is faster with respect to that at the inner side [139]. Moreover, the presence of bulky N- and C-terminal groups was a necessary and sufficient condition to obtain 3D structures from ACPC hexamers [133]. The diffusion-limited self-assembly was also at the basis of the formation of rectangular microtubes, observed by SEM and TEM, after the evaporation of a methanol/water solution of the short β-tetrapeptide Boc-ACPC_4_-OBn on a silicon surface [134]. Albeit NMR and CD experiments confirmed that, in solution, the tetramer clearly equilibrated between the 12-helix and another secondary structure and detailed PXRD analysis indicated that, in the self-assembled microtubes only, the 12-helix was present and it was stabilized by both lateral hydrophobic interactions and intermolecular hydrogen bonding of NH and C=O groups that were not implied in intramolecular H-bonds.

### 2.4. Challenges in Design and Structural Characterization of Self-Assembled β-Peptide Foldamers

Being mainly based on the basic knowledge of secondary structure and type/exact placement of side chains, as well as other important variables, such as macrodipole and facial/vertical amphiphilicity, the design of self-assembling β-peptide foldamers is somewhat less demanding in comparison to other less studied foldamers. Nevertheless, sometimes the complex interplay of variables related to interactions with both the solvent and other foldamer molecules (e.g., effects of hydrophobicity, amphiphilicity, and particular side chains in the formation of liquid crystals in aqueous environment) still has to be fully understood.

A major challenge in the field of designing self-assembling β-peptide foldamers is certainly to generate hierarchical structures which have remained so far elusive within this category, but have already been synthesized with other foldamers (e.g., nanosheets, nano- and microspheres, and nanotubes reported in Section 4 for peptoids). Another great challenge is the design of self-assemble foldamers that are biomimetic, bioactive, biomaterials, or can be used in material science, albeit the first studies that can pave the road for future improvements and new applications have recently appeared and have been reported here.

As far as structural characterization is concerned, many different challenges remain, depending on the type of self-assembled structure. In manuscripts dealing with discrete numbers of aggregated foldamers in solution, in many cases the supramolecular structures could not be precisely determined, probably due to the lack of an extensive NMR investigation exploiting the impressive capabilities of modern ultra-high field instrumentation, coupled to molecular dynamics simulations with up to date and reliable force fields. The most notable exceptions are bundles, whose atomically precise description was based on the comparison of solution data with single-crystal X-ray data.

Apart from the obvious morphological and dimensional analyses, in the few studies available on vesicles, the correct use of TEM and molecular dynamics simulations gave a good description of molecular arrangement within the wall, but the application of newer techniques (e.g., the sorting/averaging procedure with low-dose cryogenic electron microscopy used for peptoids, detailed in Section 4.2.4) could lead to an improved understanding.

For tridimensional solids, which usually have a substantial amount of crystallinity, and also for fibers whose studies were not completely directed to biological evaluation, both the precise packing of single foldamer molecules and the different degrees of higher-order assembly could be often (for solids) or sometimes (for fibers) derived by using the X-ray techniques that were more appropriate to determine them (e.g., PXRD, SAXS). However, this kind of investigation should become the rule rather than the exception for these supramolecular structures, and further developments would be highly beneficial.

## 3. Aromatic Foldamers

Aromatic foldamers are constitutively different from the β-peptides reported in the previous section, and also from the heteropeptidic sequences in Section 4, regardless of the presence or absence of amide bonds in their backbones. These “abiotic” foldamers can show very peculiar secondary and higher-order self-assembled structures, which have no counterpart even among naturally occurring folded macromolecules. In addition, different from the case of β-peptides (Section 2.3) and various nonaromatic mixed backbones (e.g., α/β-peptides, in Section 4.3), for aromatic foldamers there are no examples of studies on 3D morphologies, despite their ease of crystallization in comparison to peptide-based oligomers.

### 3.1. Discrete Self-Assembly in Solution: The Realm of Double Helices and Other Multiple Helices

#### 3.1.1. Aggregates Different from Interpenetrating Multiple Helices in Nonpolar Solvents

Apart from the impressive amount of interpenetrating double and multiple helices formed by aromatic oligoamides (AOAs), there are only a very few examples of other kinds of aromatic backbones self-assembled by the interaction of a discrete number of foldameric units.

**Figure 10:**
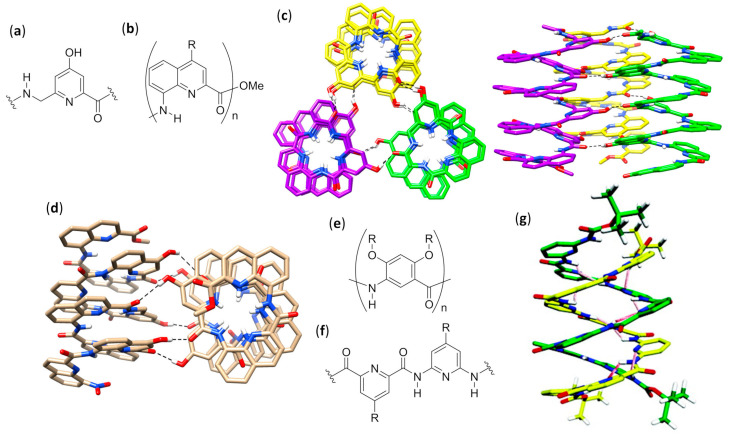
(**a**) Structure of 6-aminomethyl-4-hydroxypicolinic acid (Ref. [140]); (**b**) General structure of 8-amino-2-quinolinecarboxylic acid oligomers (Refs. [140,154]); (**c**) Top (left) and side (right) views of the *C*_3_-symmetric bundle of Ac-YQXQQYQXQQ-OMe in the crystal (helices reported in different colors, hydrogen bonds reported as dotted lines; alkoxy residues, unnecessary hydrogens, and solvent molecules omitted for clarity); (**d**) View along a helical axis of the tilted dimer of O_2_N-QXQQYQXQQ-OMe in the crystal (hydrogen bonds reported as dotted lines; alkoxy residues and unnecessary hydrogens omitted for clarity); (**e**) General structure of *meta*-linked benzene oligomers (Refs. [19,141]); (**f**) General structure of pyridinecarboxamides (PDCA) (Refs. [142,143,144,145,146,147,148,149]); (**g**) Calculated structure of 7-PDCA double helix (hydrogen bonds in pink, strands reported in different colors; side chains and unnecessary hydrogens omitted for clarity) (reprinted with permission from Ref. [146]).

Exploiting AOAs with rationally placed lipophilic and hydrogen bonding side chains, it was possible to endow single-helical aromatic foldamers with the desired possibility to form bundles in nonpolar solvents (CDCl_3_ and CD_2_Cl_2_), as thoroughly verified by various NMR techniques [140]. The aromatic oligoamides, Ac-YQXQQYQXQQ-OMe and O_2_N-QXQQYQXQQ-OMe, in which the former differed from the latter simply by an added N-terminal unit with a different derivatization, were built using 6-aminomethyl-4-hydroxypicolinic acid Y, as shown in Figure 10a, and the two 8-amino-2-quinoline carboxylic acid derivatives, X (R = OH) and Q (R = Oi-Bu), as shown in Figure 10b. Within both sequences, the hydroxyl-bearing residues (Y and X) only exposed their functionalities on one side of the helix and were thus able to form interchain hydrogen bonds with the exposed oxygen atoms of backbone amide groups. Due to the additional Y residue in the longer sequence, Ac-YQXQQYQXQQ-OMe, the disposition of Y and X residues led to two pairs of hydroxyl groups put into two distinct columnar arrangements along one helical face. This in turn allowed for the formation in both crystal and solution of an all-parallel *C*_3_-symmetric trimeric bundle, in which every foldamer molecule formed a total of eight hydrogen bonds with the neighboring helices, as shown in Figure 10c. Conversely, the lack of a double columnar regular disposition of hydrogen bond donors in the shortened sequence, O_2_N-QXQQYQXQQ-OMe, led in solution to a predominant sextuple H-bonded dimeric bundle, in which one helix axis was tilted by about 120° clockwise (or anticlockwise) for *P* (or *M*) helices with respect to a theoretical parallel head-to-head dimer, as shown in Figure 10d. However, in solution both longer and shorter sequences gave rise, albeit to a very different extent, to the equilibration between dimers and trimers, depending on solvent and temperature [140].

Additionally, foldamers built using *meta*-linked residues, reported in Figure 10e (n = 1, 2, 4, 6, and 8, R = -(CH_2_)_2_-O-i-Bu), gave self-association in chloroform, even though the aggregates were not well characterized [141]. All the oligomers up to six residues adopted crescent planar shapes, while the octamer was the only one able to complete one helical turn. Upfield of diagnostic chemical shifts and progressive signal broadening, revealed by NMR experiments, pointed out that the stacked self-aggregation was weaker for shorter oligomers and highly enhanced for longer ones. Interestingly, in this case, an opposite behavior with respect to the self-association of purely aromatic hydrocarbons, which is π-stacking- and hydrophobic effect-driven, was evidenced by both NMR and fluorescence spectra. In fact, for these AOAs, the self-assembly was enhanced in low polarity solvents (e.g., chloroform), whereas the addition of sufficient amounts of methanol, acetonitrile, dimethylformamide or dimethylsulfoxide led to dissociation. In fact, even if the single strands were not engaged in intermolecular hydrogen bonding, polar solvents still negatively affected the self-assembly of these molecules, whose stacked arrangement occurred by dipole–dipole interactions due to the presence of quite large dipole moments (i.e., amide groups and benzene rings polarized by their substituents with opposite electron-donating/withdrawing abilities).

#### 3.1.2. Double, Triple and Quadruple Helices in Chloroform

Aromatic oligoamides play a starring role in multiple helices-forming aromatic foldamers, and Huc’s group was able to exploit the predictable conformations of the single building blocks, as well as the distance and relative orientation of their amine and acid groups, to obtain a variety of interpenetrating double, triple, and quadruple helices with the desired length and diameter.

AOAs derived from alternating 2,6-diaminopyridine and 2,6-pyridinedicarboxylic acid, also called pyridinecarboxamides (PDCA), depicted by R = H in Figure 10f, and with Boc protecting groups at both termini, folded in diluted chloroform solutions into single-stranded helical structures strongly stabilized by π–π stacking interactions [142,143]. However, variation of chemical shift for many resonances, increasing signal broadening, and other NMR-derived results led us to deduce the formation of double-helices in concentrated solutions, which was also confirmed by fast atom bombardment (FAB) mass spectrometry [144,145]. The mechanism of the double helix formation of oligopyridinecarboxamides was computationally investigated for a PDCA containing seven pyridine units (7-PDCA), ascertaining that the rate-determining step was the initial introduction of the tail of a molecule into the first pitch of the other. Thus, through a series of discrete steps, involving 14 different local minima and the continuous formation/disruption process of intermolecular π-stacking and hydrogen bonds, the two molecules slipped and intertwined reciprocally, as shown in Figure 10g [146]. The double helix stability for related oligomers (R = Oi-Bu), as shown in Figure 10f, with two Boc capping groups, was further demonstrated to be strongly dependent on the length (*K*_dim_ = 3.1 × 10^4^ M^−1^ for the 7 mer and 7 × 10^5^ M^−1^ for the 9mer in CDCl_3_, while for the 11 mer and the 13 mer it was too high to be measured) [147]. On the contrary, association and dissociation rates decreased from minutes (7 mer) to days (11 mer and 13 mer). Interestingly, compared to analogs bearing flat Cbz N-terminal functions, Boc end groups led to both a slowing down of association/dissociation kinetics and an unexpected substantial increase in dimerization constants, probably due to the spring-like extension caused by Boc bulkiness, which in turn facilitated the proposed slippage mechanism with a screw-like motion. It is worth mentioning that folding allowed us to regioselectively perform N-oxidation of oligo-PDCA, as monitored by NMR, since only the two terminal 2,6-diaminopyridine groups were oxidized, whereas dimerization involved the remaining residues and the oxidized duplexes were more stable than dimers formed by their parent compounds [148]. In addition, cross-hybridization was reported to prevail over homodimeric double helices when terminally oxidized PDCA oligomers and their precursors were mixed in 1:1 ratio [149]. The rationale for both these unexpected findings was based on a subtle balance of electrostatic interactions.

**Figure 11:**
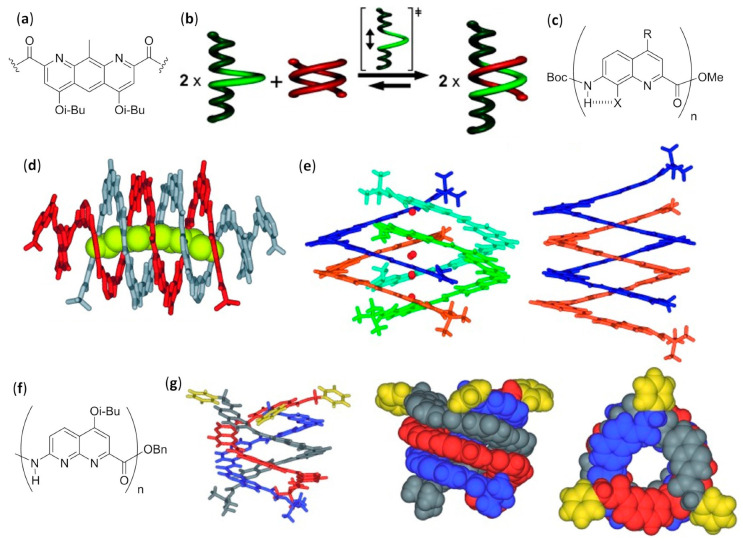
(**a**) Structure of 1,8-diazaanthracene-2,7-dicarboxylic acid unit (Refs. [19,150,151,152]); (**b**) Formation of a heterodimer by disassembly of a short double helix with a wide diameter (red) and cross-hybridization with a longer single helix with variable diameter (green) (adapted with permission from Ref. [151]); (**c**) General structure of 7-amino-8-fluoro- or 8-chloro-2-quinolinecarboxylic acid strands (Refs. [154,158,159,160]); (**d**) Crystal structure of the variable diameter single/double helical capsule containing 1,10-decanediol (strands and guest reported in different colors) (reprinted with permission from Ref. [153]); (**e**) Crystal structures of (left) quadruple helix of tetrameric 7-amino-8-fluoro-2-quinolinecarboxylic acid (X = F, n = 4 in panel c), with some water molecules reported as spheres, and (right) double helix of the octamer (X = F, n = 8 in panel c) with alkoxy residues and solvent molecules omitted for clarity (different strands reported in different colors; adapted with permission from Ref. [154]); (**f**) Structure of 2-amino-5-isobutoxy-1,8-naphthyridine-7-carboxylic acid units (Refs. [30,156,157]); (**g**) Different views and representations of crystal structure of parallel triple helix of Boc-(2-amino-5-isobutoxy-1,8-naphthyridine-7-carboxylic acid)_4_-OBn, with color-coded strands and Ph terminal groups in yellow (reprinted with permission from Ref. [156]).

Subsequently, changing the central 2,6-pyridinedicarboxylic acid of the previously studied heptamer 7-PDCA [144,145] with the 1,8-diazaanthracene-2,7-dicarboxylic acid in Figure 11a, led to a substantial increase in the diameter of the helix, as well as to a strong stabilization of the double helical hybrid (*K*_dim_ >10^7^ M^−1^ in chloroform, methanol and toluene) [150]. In fact, the enhanced π-stacking, due to the larger contact surface, and the reduced torsion angles at aryl amide bonds, diminished the enthalpic cost associated with the spring-like extension of both intercalating strands (the helical pitch passed from 3.5 to 7 Å). A very high NMR-derived self-association constant (*K*_dim_ >10^7^ M^−1^ in CDCl_3_) could also be evaluated for a short pentamer made of 1,8-diazaanthracene units. However, an interesting behavior was evidenced when this pentamer was kept in the presence of a longer analog, corresponding to the same pentamer derivatized at both sides with two tetrameric sequences of 8-amino-2-quinoline (R = Oi-Bu, n = 4), as shown in Figure 10b. The two terminal sequences prevented the self-association of the longer oligomer, due to the very small diameter of its helix at the peripheral zones, caused in turn by the peculiar directionality of amino and acid groups in 8-amino-2-quinolines. As inferred from different NMR experiments, the former double-helices of the short 1,8-diazaanthracene pentamer disassociated and the heterodimeric double-helices formed with a cross-hybridization constant *K*_a_ >10^5^ M^−1^, as shown in Figure 11b [151]. Furthermore, with the aim of obtaining a closed shell single-helical capsule with a reduced diameter at each extremity and a hollow interior able to encapsulate small guests, the same authors attached two 8-amino-2-quinoline units at each side of the previously reported heptamer made of six PDCA units, as shown in Figure 10f, with a central diazaanthracene-2,7-dicarboxylic acid residue, as shown in Figure 11a [150]. However, the sequences of just two terminal 8-amino-2-quinoline units were too short to inhibit the self-association, and the two helices interpenetrated each other more than expected, therefore completely filling the hollow interior [152]. In a following study, this approach was improved, efficiently preventing the interpenetration of the small diameter final segments, made of three 8-amino-2-quinoline units, placed at only one side of a duplex-forming tetradecameric foldamer. The remaining residues were three consecutive PDCA units, as shown in Figure 10f, followed by eight 7-amino-8-fluoro-2-quinolinecarboxylic acids, as shown in Figure 11c. These latter residues had the same substituents directionality as in pyridinecarboxamide derivatives, but quinoline carboxamides had a larger separation between acid and amine groups and gave rise to a larger internal diameter. In the self-assembled duplex, the wide double-helical segment was able to encapsulate, both in the crystal and in CDCl_3_ solution, linear guests that could simultaneously form two hydrogen bonds with the 2,6-pyridinedicarboxamides at both the extremities, as shown in Figure 11d [153].

By performing single-crystal X-ray analysis and extensive NMR investigation, the 7-amino-8-fluoroquinolinecarboxamide tetramer (X = F, R = O-i-Bu, n = 4), as shown in Figure 11c, was determined to form a quadruple helix either in the crystal or in concentrated CDCl_3_ solution at low temperature, in which two head-to-tail duplexes joined together with no substantial perturbation of their innate folding propensity, as shown in Figure 11e [154]. This occurred because the tetramer only spanned one helix turn, so that the two duplexes could compenetrate one another and give rise to stabilizing π-stacking without any great additional destabilization. Indeed, the related octamer simply dimerized to a stable antiparallel double helix (*K*_dim_ = 8.5 × 10^5^ M^−1^ in CDCl_3_ at 298 K). The possibility for the tetramer to form the quadruple helix with no additional energy cost for geometric distortions was evident by the structural similarity between each of the duplexes in the quadruplex and the double helix formed by the octamer— this can be seen by comparing the duplex formed by the orange and blue strands within the tetramer quadruple helix with the octamer double helix in Figure 11e. A further study evaluated the importance of N-H···F hydrogen bonds and repulsions between fluorine atoms and carbonyl oxygens of consecutive quinoline units, by using the chloro-substituted analogs, 7-amino-8-chloroquinolinecarboxamide foldamers (X = Cl, R = O-i-Bu, n = 4 or 8), as shown in Figure 11c [155]. While for the chloro-tetramer a rapid equilibrium in CDCl_3_ solution with labile and not-well-defined aggregated structures was evidenced, the chloro-octamer hybridized into a double helix that was almost perfectly superimposable to that of its fluorine counterpart. The crystal structure evidenced only two variations with respect to 8-fluoroquinoline units. The first was the expected slightly longer N-H···Cl hydrogen bond distance (2.5 Å) with respect to N-H···F (2.2 Å), and it was due to the difference in van der Waals radii and not to an increase in the strand curvature. The second difference was the lack of solvent molecules within the inner hollow, which in that case was almost completely occupied by the larger chlorine atoms. The helical duplex of chlorine-substituted quinoline octamer was also 10–100 times more stable than that of the corresponding fluoro-foldamer. Exploiting NMR studies, high-resolution electrospray ionization mass spectrometry (ESI-MS) and also simple thin-layer chromatography (TLC), the possibility for slow cross-hybridization between the chloro- and fluoro-substituted double helices was demonstrated, as well as the C-terminal (*S*)-phenylethylamine-biased preferential handedness induction in both homo- and heteroduplexes [155].

Despite their structural resemblance with the previously reported 8-chloro and 8-fluoroquinoline oligomers, as shown in Figure 11c, 2-amino-5-isobutoxy-1,8-naphthyridine-7-carboxylic acid foldamers, as shown in Figure 11f, were prepared in order to evaluate the effects of the loss of stabilizing H-bonds (N-H···F) and quantitatively different amide/aryl conjugations [156]. The naphthyridine rings in the oligomers easily accommodated remarkable twisting angles, thus being able to allow great strand extensions. This possibility led to the formation of both parallel and antiparallel triple helices with a very large pitch (~10.5 Å) in the solid state, as shown in Figure 11g, and in different solvents the trimerization constant measured by NMR was *K*_trim_ > 10^8^ M^−2^ in CDCl_3_ and CD_3_CN. As expected, the cavities within either the parallel or antiparallel triple helices were larger than those of both the related 8-chloro and 8-fluoroquinoline oligoamides. When a naphthyridine tetramer was linked to an end-capping 8-amino-2-quinolinecarboxylic acid trimer through a 2,6-diaminopyridine unit, duplexes with an antiparallel orientation and a large double-helical central section flanked by two single helices were obtained [157], exploiting the same strategy previously used for 7-amino-8-fluoro-2-quinolinecarboxylic acid strands [153]. The rational use of different tactically placed aromatic amino acids endowed the foldamer with the correct propensity to dimerize with curvature and recognition ability, and gave rise to capsules capable to effectively bind a very polar guest—citric acid—not only in the crystal but also in polar and protic competitive solvents, as shown by NMR spectra.

Foldamer-based rotaxanes, the so-called foldaxanes, have also been obtained from double helices of aromatic foldamers. The antiparallel double helix of heterononamer Piv-PDCA_3_-(7-amino-8-fluoro-2-quinolinecarboxylic acid)_6_-Boc was used in chloroform as a host for a rod-like bichromophoric guest, whose Ru(bpy)_3_^2+^ and pyrene chromophores were separated by a linear spacer with a central 1,8-octyldiamine dicarbamate [158]. Reversible electronic energy transfer (REET) was allowed when the chromophoric units could approach each other in the flexible free guest, originating an increased luminescence lifetime with respect to the separated chromophores. On the other hand, the slow unwinding/rewinding of the double helix around the guest rigidified the rod-like guest itself and firmly distanced the chromophores, leading to the recovery of their individual photophysical behaviors. This slow process was necessary because the size of both chromophores was too large to permit the direct threading/unthreading fast equilibrium. Therefore, the real-time foldaxane formation could be followed by monitoring changes in luminescence lifetime, and the results were in good agreement with those obtained by ^1^H NMR kinetic experiments.

**Figure 12:**
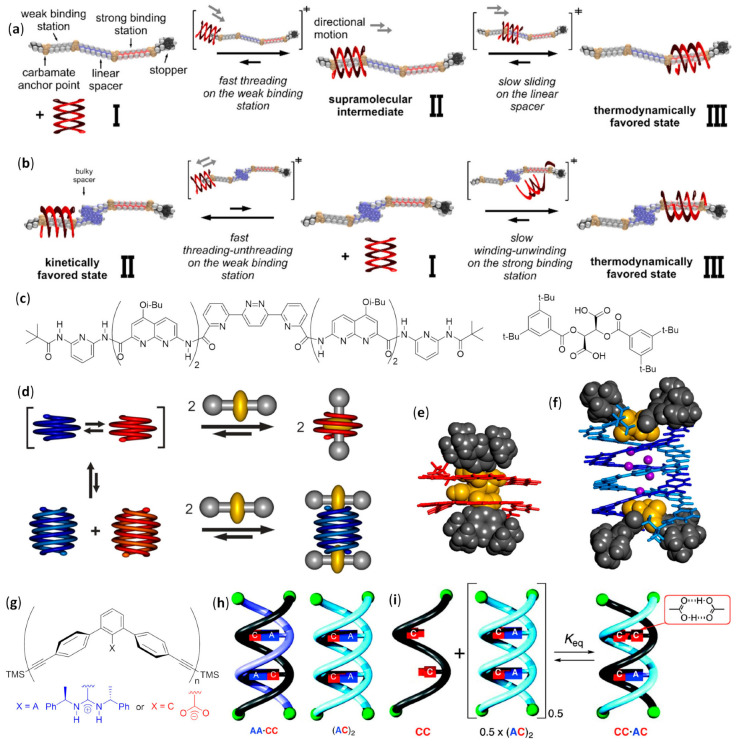
Different pathways followed by the double helix (host) when the double station rod (guest) has (**a**) a non-bulky spacer (sliding pathway) and (**b**) a bulky spacer (unwinding/rewinding pathway, bottom), respectively (strands are stylized and color-coded, while the guest is in space-filling model; adapted with permission from Ref. [159]); (**c**) Structure of heptameric foldamer and tartrate derivatives implied in equilibria reported in panel d (Ref. [161]); (**d**) Overall equilibrium between *M* (red) single-helix foldaxane with (*L*)-tartrate and *P* (blue) double helix complexed with two (*L*)-tartrate molecules (reprinted with permission from Ref. [161]); Computed structures of (**e**) 1:1 (*M*)-helix⸧(*L*)-tartrate foldaxane and (**f**) 2:2 host–guest complex [(*P*)-double helix]_2_⸧[(*L*)-tartrate]_2_ (adapted with permission from Ref. [161]); (**g**) General structure of *m*-terphenyl foldamers (Refs. [162,163,164]); (**h**) Examples of self-complementary (AC)_2_ and complementary duplexes (AA·CC) (A = *m*-terphenyl diamidine, C = *m*-terphenyl acid; reprinted with permission from Ref. [163]); (**i**) Equilibrium between (AC)_2_ and CC·AC (A = *m*-terphenyl diamidine, C = *m*-terphenyl acid; adapted with permission from Ref. [163]).

A related heteroundecamer host with two additional quinoline units, Piv-PDCA_3_-(7-amino-8-fluoro-2-quinolinecarboxylic acid)_8_-Boc, was also used with rationally designed rod-like guests with a very bulky stopper at only one extremity [159]. These experiments followed the first experiences with foldaxanes made of the same double-helical foldamer threading on mono-station (dicarbamates) or double-station (two dicarbamate anchoring stations) rod-like guests, for which no end-capping was used. These prototypical double-helical foldaxanes had already showed no particular selectivity in terms of guest length for mono-station guests, and also in terms of distances between hydrogen bonding carbamate functions in double-station guests [160]. However, in this further study, the carbamate groups properly placed along the backbones of singly end-capped rods gave rise to multiple-station foldaxanes, being able to form either weak (α-station) or strong (ω-station) hydrogen bonds with both H-bond accepting and donating groups of the host exposed toward the cylindrical cavity, as shown in Figure 12a [159]. These different H-bonding strengths in turn depended on the fact that the distances between the two carbamate functions within each station could exactly (ω-station) or imperfectly (α-station) align with the hydrogen bonding partners in the first and last turns of the double helix. In addition, the spacer groups separating the differently binding stations could be either non-bulky alkyl/oligoethylene glycol linear chains or bulky aromatic fragments, which then completely inhibited sliding. The host–guest behavior of foldaxanes, involving spacers with different bulkiness, was kinetically and thermodynamically evaluated carrying out time-dependent ^1^H NMR experiments in CDCl_3_, whereas the most stable species could also be isolated as single-crystals and submitted to X-ray analysis. The antiparallel double helices were shown to rapidly thread on the weakly binding α-station and then, for guests with linear spacers, they reached the thermodynamically favored ω-station by sliding within a few hours. On the contrary, when bulky spacers did not allow direct sliding from the α-station to the ω-station, the fast threading/unthreading equilibria on the weakly binding α-station was followed by the very slow unwinding/rewinding process directly on the strongly binding ω-station, which necessitated many days to reach the equilibrium, as shown in Figure 12b.

A peculiar complex was found when dumbbell-shaped derivatives of either the *L* or *D* enantiomer of tartaric acid, with bulky groups at the alcohol functions, were used as guests for an aromatic foldamer designed to form a single-helical host with a large internal cavity. In order to avoid double helix formation, two pivaloyl amide terminal functions were placed on an heptameric heterofoldamer composed of a central pyridine–pyridazine–pyridine segment flanked at both sides by two naphthyridine units and a terminal 2,6-diaminopyridine, as shown in Figure 12c [161]. The usual NMR experiments at different times pointed out that the desired foldaxanes rapidly formed (less than 5 min for a 0.1 mM CDCl_3_ foldamer solution at 298 K with six equivalents of tartrate). Helix handedness was driven by the chirality of the guest (i.e., the 1:1 foldaxanes (*M*)-helix⸧(*L*)-tartrate or (*P*)-helix⸧(*D*)-tartrate selectively formed, depending on the tartrate chirality, as shown in Figure 12d, due to selective hydrogen bonding between the two acidic functionalities of tartrate derivatives and the cavity-exposed H-bond accepting and donating groups of the single helix, as confirmed by X-ray single-crystal diffraction, as shown in Figure 12e. However, the same structural determinations illustrated that the long-lived mono-helical foldaxanes interconverted very slowly (more than 10 days) into 2:2 host–guest complexes in which two molecules of (*L*)- or (*D*)-tartrate were bound to both extremities of a (*P*)- or (*M*)-double helix, respectively, as shown in Figure 12f.

The first example of single-handed double helices was obtained, both in the crystal and in CDCl_3_ solution, when a chiral *m*-terphenyl diamidine (X = A, n = 2) based on (*R*)-phenylethylamine formed a right-handed duplex stabilized by two salt bridges with the corresponding achiral *m*-terphenyl diacid (X = C, n = 2), as shown in Figure 12g, even if CD and quantitative ^1^H NMR experiments in DMSO exhibited about one third of dissociated species in 0.1 mM solutions [162]. Double helices were further obtained by selective pairing of single strands of two to four *m*-terphenyl units, functionalized with either complementary or self-complementary sequences—examples of dimers are shown in Figure 12h—demonstrating the sequence- and length-specificity of double helices formation. However, two apparently mismatched strands were also able to form stable heteroduplexes exploiting doubly hydrogen bonded acid units, C·C, instead of conventional amidine-carboxylic acid A·C salt bridges, depicted by K_eq_ ~1 in Figure 12i. The duplexes formed in complex mixtures were very stable and could be separated by HPLC [163]. In addition, the recognition between *m*-terphenyl amidines and acids was further demonstrated to be possible even using linkers different from the linear diethynyl units [164].

#### 3.1.3. Double Helices in Water

The self-association of single helical strands to give double helices could also be performed in an aqueous environment, as ascertained by NMR and ESI-MS. In these cases, the authors used water-soluble foldamers made of four or six 7-amino-8-fluoro-2-quinolinecarboxylic acid monomers (X = F, R = O(CH_2_)_3_NH_3_^+^CF_3_CO_2_^−^; n = 4 or 6), as shown in Figure 11c, followed by three 8-amino-2-quinolinecarboxylic acid residues (R = O(CH_2_)_3_NH_3_^+^CF_3_CO_2_^−^; n = 3), as shown in Figure 10b, with both termini unprotected [165]. The C-terminal 8-amino-2-quinolinecarboxylic acid residues were used as end-capping strands to prevent self-association from that side, so that only antiparallel hybridization was possible. Even if duplexes formation was found to be negligible in DMSO-*d_6_*, the dimerization constant (*K*_dim_) increased sharply with the water content in the mixture. Only double helices could be found above 30% water, presumably due to hydrophobic effects and the *K*_dim_ values being too high to be measured by NMR and much larger than those for the non-water-soluble analogs previously studied [154].

### 3.2. Extended Self-Assembly: Monolayers, Vesicles, and Fibers

#### 3.2.1. Self-Assembled Monolayers

Single-helical quinolinecarboxamide foldamers spanning a wide interval of lengths (5 to 33 units; that is from 1.2 to 5.7 nm in the folded conformations) were used to form SAMs on gold, exploiting the tritylated thiol group in its unprotected form at the N-terminus, or on silicon, by linking the N-terminal alkyne group to the azide-derivatized SiO_2_ surface [166]. In both cases, an almost orthogonal disposition of helices with respect to the surface was evidenced by conductive-probe AFM (C-AFM), a technique that also allowed us to measure the current intensity as a function of the applied bias. Additional analyses by ellipsometry, contact angle measurements, cyclic voltammetry, and polarization modulation-IR reflection absorption spectroscopy (PMIRRAS) led to the determination of the charge transport mechanism. The vertical charge transport measured on SAMs on a gold surface was efficient and showed a very reduced increase in resistance with the oligomer length, 0.06 Å^−1^, in agreement with the hopping mechanism. On the contrary, the horizontal charge transport measured for SAMs on silica highlighted the absence of lateral conduction. The insulating behavior was ascribed to the very low lateral charge mobility, due to the lack of π-stacking interactions among adjacent helices, which in turn was caused by the steric bulkiness of isobutoxy side chains of quinoline units.

A pyridinecarboxamide (PDCA) pentamer, derivatized with a thiol anchoring point at the central residue and two tetrathiafulvalene units as electroactive probes, was synthesized with the aim of recognizing anions inside the cavity by using cyclic voltammetry [167]. Experimental variables were chosen in order to minimize double helices formation and maximize the coverage of the gold electrode by helices, which lay parallel to the surface. Both the selectivity and reversibility of anion binding (Cl^−^ or HSO_4_^−^) to electropositive amide protons within the internal cavity could be demonstrated. A strictly related foldamer, in which the thiol anchoring point was substituted with an isobutyl chain, was either chemically or electrochemically oxidized with thianthrenium tetrafluoroborate, thus generating radical cations at both tetrathiafulvalene redox centers [168]. As a result, spectroelectrochemical analyses of variations in UV–vis absorption spectra evidenced that the weak dimerization tendency for the neutral species in 1:1 CH_2_Cl_2_/CH_3_CN was increased by a factor of ~100 for diradical dications, even though the exact arrangement was not determined.

Self-assembled monolayers of foldamers based on four triazole units alternated with *meta*-derivatized benzene rings, also bearing water-soluble chains, were linked to the gold surface by thiols and used for the reagentless anion sensing in water [169]. These short foldamers were built utilizing either hydrogen- (hydrogen bonding) or iodine-derivatized (halogen bonding) triazole rings exposed toward the cavity, and their recognition ability was investigated by a non-redox technique—electrochemical impedance spectroscopy (EIS). Especially in their halogen-bonding version, they were able to selectively bind, with low limits of detection, different biologically and environmentally relevant guests (thiocyanate, iodide, and perrhenate) in water.

**Figure 13:**
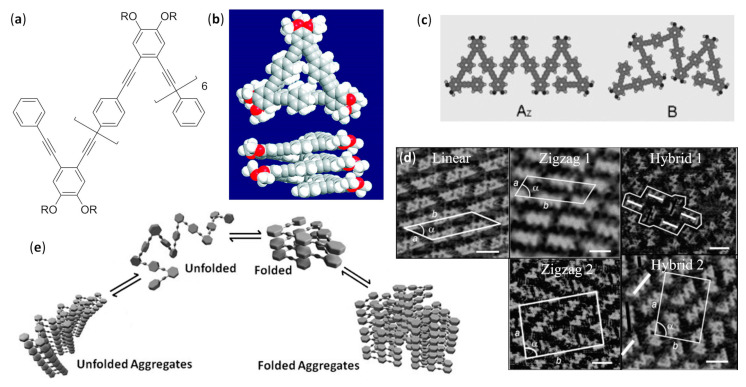
(**a**) Chemical structure of *o*-phenyleneethynylene-*alt*-*p*-phenyleneethynylene (OPE) foldamers (Refs. [170,171,172,173]); (**b**) Secondary structure of a pentadecameric OPE foldamer with R = *n*-dodecyl (dodecyl side chains truncated as methyl groups) (adapted with permission from Ref. [170]); (**c**) A_Z_ and B foldings of OPE with R = *n*-dodecyl (side chains omitted for clarity) and (**d**) STM images of their self-association in different conditions of solvent and concentration (zigzag 1 in TCB, zigzag 2 in 1-phenyloctane and tetradecane, hybrid 1 in heptanoic acid, hybrid 2 in 1-phenyloctane and tetradecane, linear in heptanoic acid, 1-phenyloctane, and tetradecane; scale bars: 4 nm) (adapted with permission from Ref. [171]); (**e**) Representation of equilibria involved in single molecules and aggregates of OPE foldamers (see text for explanations) (adapted with permission from Ref. [173]).

A pentadecamer made of alternating *ortho*- and *para*-substituted benzene rings rigidly linked together by ethynylene units [oligo(*o*-phenyleneethynylene-*alt*-*p*-phenyleneethynylene), OPE] was demonstrated to be a random coil in chloroform. Due to the solvophobic effect on aromatic rings, which then preferred to stack by π–π interactions, it assumed a helical folding in apolar aliphatic solvents, as shown by UV–vis absorption and fluorescence emission spectra, while the solvophilicity of long aliphatic peripheral chains guaranteed solubility (R = *n*-C_12_H_25_), as shown in Figure 13a,b [170]. Within the foldamer, *para*-phenylene units were necessary, because an oligomer only composed of *ortho*-phenylene residues should otherwise assume exaggerated dihedral angles in order to allow the correct π–π stacking distance among aromatic units in consecutive helical turns. The same OPE foldamer showed a number of possible secondary structures when it was extensively analyzed by high-resolution STM (scanning tunneling microscopy) at the liquid/HOPG (highly oriented pyrolytic graphite) interface [171]. In fact, many different foldings were detected in solution, depending on temperature, concentration and, above all, the solvent (heptanoic acid, 1-phenyloctane, 1,2,4-trichlorobenzene or tetradecane). All the secondary conformations featured the preferred *transoid* and *cisoid* backbone conformations, but in ordered SAMs, at interface only, the A_Z_ and B in-plane arrangements were found, as shown in Figure 13c, due to the fact that helical structures could only have limited stabilizing interactions with the substrate (HOPG). In addition, the preferred self-association mode dramatically changed with the experimental variables, due to a complex interplay of interactions of either the conjugated or aliphatic portions with the substrate, as well as contacts among flexible side chains and different preferential solvations (i.e., trichlorobenzene is a good solvent for both the aromatic and aliphatic moieties, while tetradecane poorly solvates aromatic rings). The overall effects could lead to various arrangements: two SAMs exclusively composed of molecules in the A_Z_ conformation, the zigzag 1 (in TCB, 9.4 × 10^−6^ M) and zigzag 2 monolayers (in 1-phenyloctane, 8.0 × 10^−6^ M), or in alternative two hybrid arrangements, hybrid 1 (in heptanoic acid, 1.2 × 10^−6^ M) and hybrid 2 (in 1-phenyloctane, 1.0 × 10^−6^ M), in which A_Z_ and B conformations are in a 2:1 and 1:1 ratio, respectively, and eventually the purely B-type linear assembly (in heptanoic acid, 3.0 × 10^−7^ M), as shown in Figure 13d. Furthermore, it was demonstrated that the three SAMs containing the B conformation (hybrid 1, hybrid 2, and linear) were composed of chiral domains in which only B_R_ or B_L_-type conformations could be found, due to the fact that the B arrangement had no symmetry plane and was then prochiral [172]. Even the two different couples of adjacent A_Z_-type molecules observed in the zigzag 2 monolayers were chiral, but the alternating disposition of the chiral dimers made the higher-order self-assembled superstructure a racemic mixture of the two enantiomeric pairs, and then achiral.

A completely different behavior was found for other OPE pentadecameric foldamers bearing linear or branched side chains that could be either short aliphatic groups or more polar oligo(ethyleneglycol) fragments, depicted by R in Figure 13a [173]. These foldamers, in striking contrast with the previously investigated species having dodecyl groups that always gave SAMs [170,171,172], furnished aggregates whose structures varied with the solvent. As expected, helices, such as those in Figure 13b, were strongly promoted by solvents able to preferentially solvate side chains with respect to backbone aromatic rings. Thus, CD and UV–vis absorption spectra underlined that, in chloroform random coils were always formed, due to good solvation of backbone and both types of side chains, while *n*-hexane and methanol favored helical foldings of OPE foldamers with aliphatic and oligo(ethyleneglycol) side chains, respectively. The judicious use of the same experimental techniques also allowed us to draw interesting conclusions about the aggregation behavior. In fact, when the OPE oligomer with short branched aliphatic groups was dissolved in cyclohexane, the slight decrease in the solvation of side chains with respect to *n*-hexane led to a destabilization of helices and shifted the equilibrium toward the gradual formation of unfolded aggregates, which probably also had a stacked superstructure of planar molecules, as shown in Figure 13e. This phenomenon did not occur for the OPE foldamer with linear aliphatic side chains, highlighting the very subtle balance involved. On the other hand, when more than 70% water was added to the methanolic solution of OPE oligomer with oligo(ethyleneglycol) side chains, the equilibrium slowly shifted toward the formation of helically folded aggregates, albeit unfolded aggregates were still found to be the kinetic products, as shown in Figure 13e.

#### 3.2.2. Vesicles: Effects of Varying the Solvent Composition

**Figure 14:**
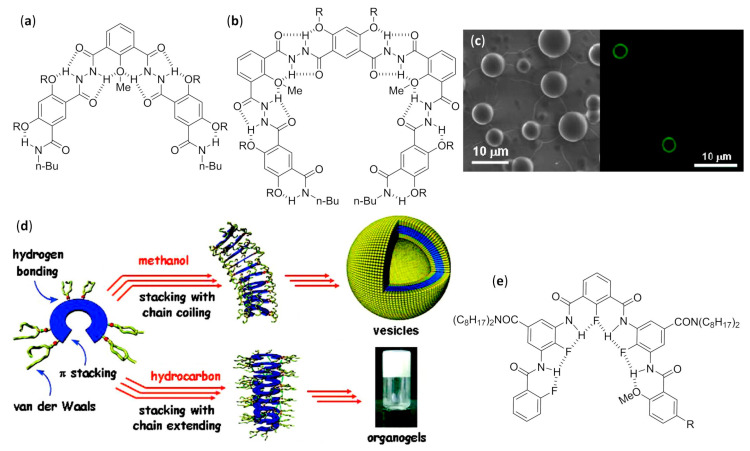
(**a**,**b**) General structures of aromatic hydrazide foldamers studied (Refs. [174,175]); (**c**) Representative SEM and fluorescence images of micelles formed by aromatic hydrazide foldamers in methanol (reprinted with permission from Ref. [174]); (**d**) Tentative model for the formation of vesicles (in methanol) and organogels (in hydrocarbon solvents) (adapted with permission from Ref. [174]); (**e**) Structure of a vesicles-forming arylamide foldamer with a continuous pattern of bifurcated intramolecular hydrogen bonds (Refs. [176,177]).

The first self-assembly of nonamphiphilic foldamers devoid of hydrophilic fragments to give vesicles was obtained by Li’s group dissolving the short hydrazide-based oligomers in methanol, as shown in Figure 14a,b, in which the R groups featured Gly or Ala residues, both derivatized as C-terminal *N*,*N*-octyl tertiary amides [174]. The complex intramolecular hydrogen bonding pattern with bifurcated H-bonds resisted even in the presence of competitive solvents, as it was ascertained by NMR experiments. The vesicles, as shown in Figure 14c, always displayed a very reduced size distribution, even if the average diameter apparently changed with the technique used to determine it (AFM, fluorescence microscopy or dynamic light scattering, DLS), and their monolayered and hollow nature was likely due to the cooperative effect of van der Waals interactions, hydrogen bonding, and solvophobic effects. In particular, when methanol was used, van der Waals interactions and solvophobic effects caused the coiling of lipophilic chains, which, in turn, favored the curvature of the stacked self-assembling structure in order to expose to the solvent the minimum possible apolar surface area, and ultimately led to the observed spherical morphology, as shown in Figure 14d. In contrast, when *n*-hexane, cyclohexane, *n*-heptane, *n*-octane, or *n*-decane were used as the solvent, the lipophilic chains of terminal tertiary amides could extend—thus allowing both the linear stacking and the intertwining process with side chains of other rod-shaped growing superstructures—and entangled fibers with organogelator effect were obtained.

In a further study, other derivatives of the planar hydrazide foldamer in Figure 14a, having R groups devoid of the initial amino acid residue but still conserving either secondary or tertiary amides bearing different linear aliphatic chains, were analyzed by TEM, SEM and AFM [175]. In methanol, the presence of *N*,*N*-dialkyl amides was shown to be essential for the solubility, but the loss of the first hydrogen bonding amide unit within the R fragments, originally belonging to a Gly residue in the previously studied hydrazide foldamers [174], caused the formation of membrane-like structures instead of vesicles. This demonstrated the importance of the H-bonding NH in the Gly residue, which contributed to both the strength and directionality of the stacking among foldameric backbones. However, using methanol/water mixtures as the solvent, the soluble derivatives with shorter alkyl chains in tertiary amides (i.e., *N*,*N*-diethyl and *N*,*N*-dibutyl) evidenced a gradual change in the generated morphologies, shifting toward gelating fibers as the water content increased. On the other hand, coherently with the previously studied *N*,*N*-dioctyl derivative [174], foldamers whose R groups had *N*,*N*-dihexyl and *N*,*N*-didodecyl terminal amides were also able to easily gelate linear, and also sometimes cyclic/aromatic hydrocarbons. This was due to an increase in gelating capacity for foldamers bearing longer alkyl chains that in turn could better interact with solvent molecules.

Other hollow vesicles, again with a monolayered wall, were obtained in chloroform/methanol mixtures from a short arylamide foldamer (R = H), as shown in Figure 14e, having a continuous pattern of bifurcated intramolecular hydrogen bonds involving amide NH and fluorine or oxygen atoms. TEM, AFM, and SEM analyses confirmed that its dipodal *C*_2_-symmetric version, visible in the R group in Figure 14e, was also the same sequence but with an inverted order and furnished empty vesicles [177]. Differently from the previously studied arylhydrazides, in this case there was no need for intermolecular hydrogen bonding, because of the increased π-stacking ability, as evidenced by NMR and the single-crystal X-ray diffraction of samples obtained by evaporation. The same reason led to a much more pronounced tendency of the dipodal derivative to form vesicles in both less and more polar mixtures (from methanol:chloroform 90:10 to 30:70), with respect to the simpler parent compound, depicted by R = H in Figure 14e, that furnished vesicles only in a very restricted range (from methanol:chloroform 55:45 to 45:55).

#### 3.2.3. Vesicles in Water

**Figure 15:**
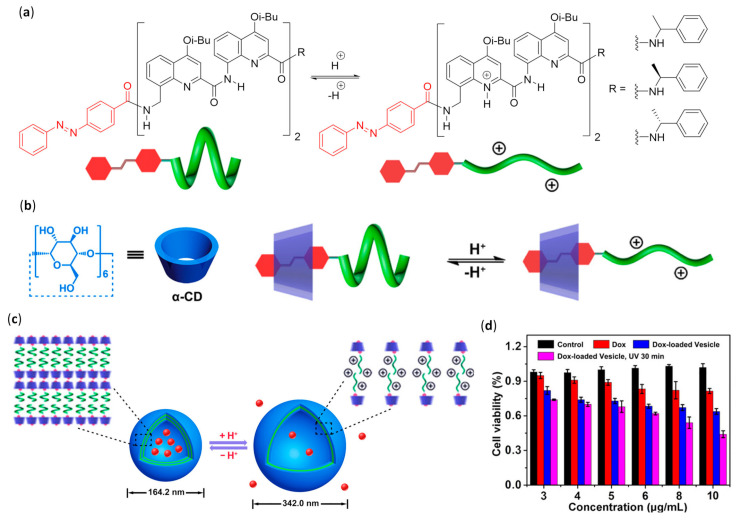
(**a**) Acidification-driven unfolding of a foldamer based on 8-amino and 8-aminometyl-2-quinoline carboxylic acid (Ref. [180]) alternating units (green), having an N-terminal azobenzene group (red) (adapted with permission from Ref. [178]); (**b**) Folding/unfolding equilibrium of host–guest complexes with α-cyclodextrin (cyan) (adapted with permission from Ref. [178]); (**c**) Morphological changes of vesicles triggered by pH variations (adapted with permission from Ref. [178]); (**d**) MCF-7 cancer cells viability upon incubation with free vesicles (black), doxorubicin (red), doxorubicin-loaded vesicles with (fuchsia) and without (blue) UV light irradiation, respectively, as a function of DOX concentration (adapted with permission from Ref. [178]).

A helical foldamer based on 8-amino and 8-aminometyl-2-quinoline carboxylic acid units, derivatized with phenylethylamine at the C-terminus, was chosen to form host–guest complexes by recognition between the azobenzene group at its N-terminus and α-cyclodextrin (α-CD), as shown in Figure 15a,b, as demonstrated by NMR spectra in D_2_O/DMSO-*d*_6_ mixtures. Then, the self-assembly in neutral aqueous solution to give multilayered vesicles formed by supra-amphiphilic helices, as shown in Figure 15c, was studied by TEM and cryo-TEM microscopies, as well as CD and DLS measurements [178]. However, the acidification-driven unfolding, due to the selective protonation of pyridine nitrogen atoms in 8-aminometyl-2-quinoline units, triggered a slow (~2 h) increase in size, which almost doubled, and the pronounced reduction in the wall thickness (from 25.6 to 3.9 nm), due to the simple bilayered structure present at pH = 4. The pH-responsive process was shown to be completely reversible, as shown in Figure 15c, for many cycles, and a related similar behavior was evidenced when the vesicles were alternately irradiated with UV and visible light. The UV-driven *trans*-to-*cis* photoisomerization of the azobenzene moiety caused the decomplexation of α-CD and the formation of irregular globular aggregates, which in turn again gave a reversible formation of vesicles induced by the inverted *cis*-to-*trans* photoisomerization upon irradiation with visible light. Interestingly, the increase in permeability obtained by the thinner membrane after acidification and the disassembly caused by UV-irradiation led, respectively, to a moderate and substantial release from carboxyfluorescein-loaded vesicles. Eventually, the delivery of encapsulated doxorubicin, which was visualized by confocal laser scanning microscopy (CLSM), allowed the efficient and highly concentration-dependent killing of MCF-7 cancer cells after UV-induced release, as shown in Figure 15d. In a further study, the vesicles formed by the *M*-helices of a quinoline foldamer with a different chiral group as the C-terminus, demonstrated extremely similar morphologies, behaviors, and capabilities. In that case, the *R* enantiomer of entrapped racemic drug propranolol could preferentially permeate through the intact multilayered membrane, albeit with a low selectivity (enantiomeric excess ~24%) [179].

#### 3.2.4. Fibers: Effect of Varying the Water Content

**Figure 16:**
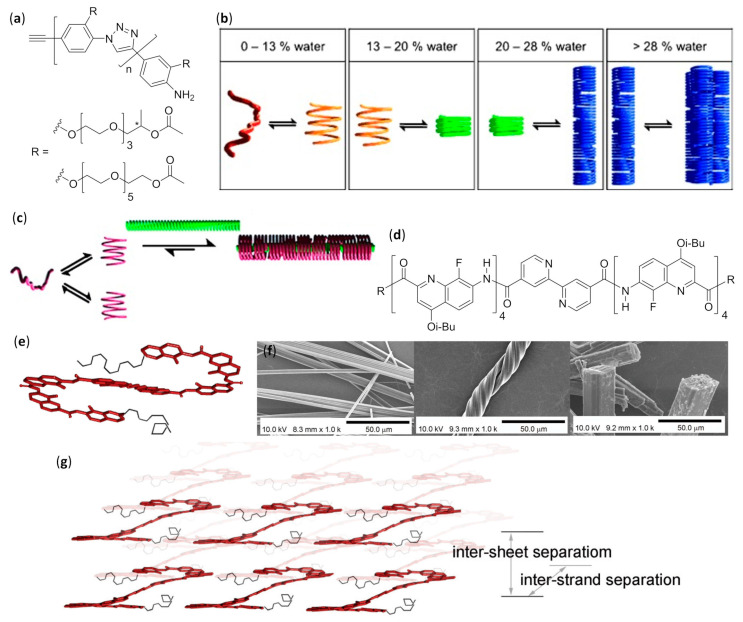
(**a**) Chemical structures of poly(*p*-AT)s (Ref. [181]); (**b**) Equilibria involved in the aqueous hierarchical self-assembly of poly(*p*-AT)s as the water content increases (random coil in red, loose spring in yellow, helix in green, nanotube and bundle in blue) (reprinted with permission from Ref. [181]); (**c**) Diastereoselective formation of host–guest nanotubes by interaction of achiral poly(*p*-AT) (purple) with the rigid α-helix of poly(γ-benzyl-L-glutamate) (green) (reprinted with permission from Ref. [181]); (**d**) Chemical structure of 8-fluoroquinolinecarboxamide and bipyridyl-based oligomers (Refs [154,182]) and (**e**) their S-shaped folding (backbone in red sticks; terminal alkyl chains in black wireframe) (adapted with permission from Ref. [182]); (**f**) SEM images of structures formed by foldamers with R = NHC_6_H_13_ (hollow tubes, left), NHC_12_H_25_ (twisted fibers made of hollow fibrils, middle), and NHC_18_H_37_ (rods, right) (adapted with permission from Ref. [182]); (**g**) Common packing mode of different S-shaped foldamers giving the morphologies in panel (**f**) (adapted with permission from Ref. [182]).

Polymeric foldamers of *para*-aryl-triazole, *p*-AT, in which 1,4-disubstituted triazoles and phenyl rings bearing oligo(ethyleneglycol) side chains adopted a preferred *cisoid* conformation, as shown in Figure 16a, were synthesized to form helices with a reduced curvature and a large internal diameter (30.6 Å) [181]. As deduced by CD spectra and the deconvolution of UV–vis absorption spectra, when the water content in aqueous dimethylformamide (DMF) increased, the enhanced hydrophobic effect at first led to the random coil-“loose spring” equilibrium, and then these latter structures were observed to equilibrate with helices above 13% water, as shown in Figure 16b. The helical structures were also shown to have a favored handedness when chiral groups were inserted into side chains. Further increases in water percentage caused the self-assembly of nanotubes from the stacking helices, and eventually long fiber-like bundles were formed, as shown in Figure 16b, and visualized by TEM and cryo-TEM microscopies. The hierarchical self-assembly was further favored using hydrophobic poly(γ-benzyl-L-glutamate) α-helical templates of different lengths, and loose springs of poly(*p*-AT)s, made of achiral units, were ascertained to diastereoselectively fold around the guest and match its length, as shown in Figure 16c.

#### 3.2.5. Fibers in Organic Solvents

S-shaped foldamers were built by adding two tetrameric 8-fluoro-quinolinecarboxamide helical strands to a central bipyridyl segment in the *trans* conformation, as shown in Figure 16d,e, and their shape and H-bonding pattern were determined by NMR experiments [182]. Their dissolution into 1:1 chloroform/methanol mixtures allowed us to observe by SEM microscopy different morphologies, depending on the length of terminal alkyl chains, as shown in Figure 16f, upon standing for two days, but X-ray diffraction and TEM analyses after 2 h highlighted common fibril-like precursors with the same packing mode, as shown in Figure 16g. Even if the generation of different final objects from these fibrils was only qualitatively explained, the formation of such common precursors was due to the assembly of preliminary sheets of π-stacked S-shaped foldamers, in which intermolecular H-bonding and van der Waals forces played an important role, and the aliphatic chains were oriented toward the aromatic portions. This first self-assembly was followed by a sequential aggregation in multiple sheets and then in fibril-like precursors.

**Figure 17:**
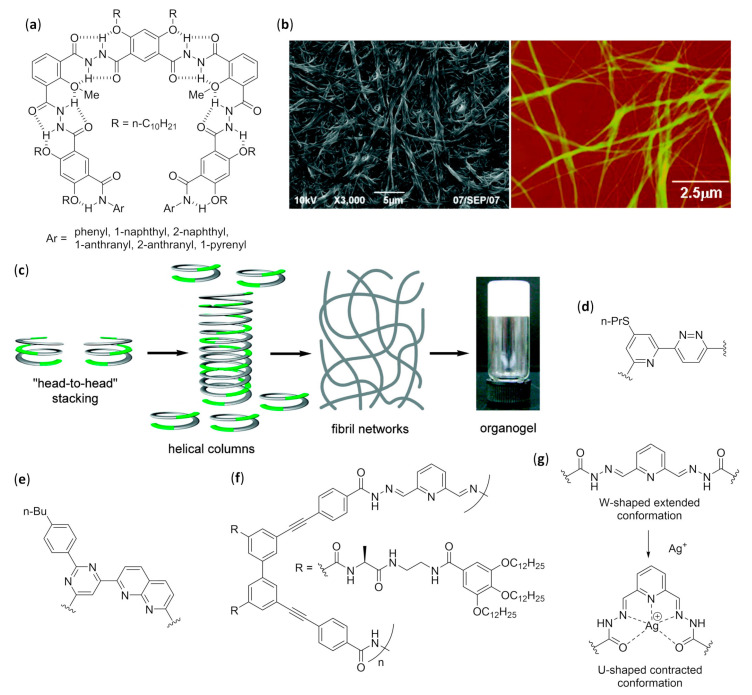
(**a**) General structures of aromatic hydrazide foldamers studied (Refs. [174,183]); (**b**) Representative SEM and AFM images of dried gels obtained from 0.1 mM solutions of a selected hydrazide foldamer in (left) *n*-octanol and (right) toluene (adapted with permission from Ref. [183]); (**c**) Representation of the gelation process induced by hydrazide foldamers (hydrazide strand in gray, appended arene units in green, decyl side chains omitted for clarity): the head-to-head stacking of foldamers gives rise to long columnar alignments, which in turn form bundles and then the gelating fibrils by interdigitation of alkyl side chains (adapted with permission from Ref. [183]); (**d**) Pyridine–pyridazine alternating units in the *transoid* conformation (Refs. [185,186,187]); (**e**) Naphthyridine–pyrimidine alternating units in the *transoid* conformation (Refs. [188,189]); (**f**) General structure of conformationally switchable foldamers and (**g**) their Ag^+^ coordination-driven conformational switch (Ref. [190,191]).

Li’s group analyzed, by UV–vis, fluorescence, induced circular dichroism (ICD), X-ray diffraction, and electron microscopies (TEM and SEM), the tendency to self-assemble nonhydrophilic and nonamphiphilic heptameric hydrazide foldamers, only differing from the pentamers reported in the previous subsection, as shown in Figure 14b [174], by two additional aromatic units appended at both termini, and the presence of *n*-decyl side chains instead of amino acid *N*,*N*-dialkylamides, as shown in Figure 17a [183]. The phenyl, α- or β-naphthyl, 1- or 2-anthranyl, and 1-pyrenyl terminal derivatizations always led to organogelation with reduced amounts in very different organic solvents, from nonpolar alicyclic hydrocarbons to aromatics, as shown in Figure 17b, esters, alcohols, and 1,4-dioxane, and in a few cases they behaved as “supergelators” (<0.1% wt). In addition, the complexation of added octylated glucose in the same solvents further enhanced the gelation abilities and gave chiral supramolecular helicity, even when substoichiometric amounts of glucose derivative were used, due to the “sergeant and soldiers” effect [184]. In fact, the initially generated chiral helix hosted a glucose molecule and could lead to chirality amplification by selective induction of the same helix handedness in sequentially assembling components lacking the chiral guest. However, with the obvious exception of the first 1:1 host–guest interaction between helical aromatic oligohydrazides and derivatized chiral sugar molecules, this effect did not formally change the other equilibria involved in the overall mechanism. As reported for the uncomplexed helices in Figure 17c, in the initial stage, single molecules of hydrazide foldamers assumed the helical conformation, with the appended arene units (indicated in green) disposed in order to maximize intramolecular π-stacking. Then, the helices were stacked in a head-to-head fashion, which also maximized intermolecular π–π interactions, to form columnar alignments. At this point, the van der Waals-driven interdigitation of decyl chains led to the formation of bundles and then fibrils, which ultimately gelated the solvent.

Lehn and coworkers also used helical aromatic foldamers to hierarchically self-assemble protofibrils, fibrils, and then fibers [185,186]. The helical arrangement of a 13-mer was generated by the alternating 2,6-pyridine and 3,6-pyridazine units, as shown in Figure 17d, that, in spite of the lack of the typical hydrogen bonding patterns, preferred the reported *transoid* conformation for all the single bonds linking adjacent heterocycles. This was corroborated by molecular dynamics simulations, that pointed out the tendency to fold into a helix with about 10 residues per turn and the π–π stacking interaction between the two terminal pyridines (distance ~3.4 Å), as well as by density functional theory (DFT) calculations. In fact, apart from the expected stabilizing π–π and van der Waals interactions between the terminal heterocycles in the helical arrangement, there were weak nonclassical N···H-C hydrogen bonds. An additional stabilization came from the antiparallel orientation of dipoles on nitrogen, when the neighboring heterocycles assumed the *transoid* conformation, which also avoided destabilizing steric interactions between *ortho* hydrogens. Variable-concentration ^1^H NMR experiments and vapor pressure osmometry showed that, in chloroform the preferred self-association is the stacked dimerization, even if AFM analyses confirmed that monolayers were formed from chloroform solutions at the air–water interface (Langmuir–Blodgett films) and on mica. However, freeze-fracture electron microscopy evidenced the higher-order hierarchical self-assembly in fibers obtained in diluted dichloromethane and pyridine solutions.

Subsequently, the same foldamer was shown to produce mirror symmetry breaking and chiral amplification in the fibers observed by TEM and AFM, the initial chiral bias being likely due to the residual chirality in the starting material. Variation of CD spectra in dichloromethane, pyridine, and 1,2-dichloroethane with time, temperature, and chiral additives led to the conclusion that ultrasound-aided dissolution favored the partial breaking up of mother fibers with a certain handedness, and the further formation of daughter fibers that maintained the same chirality, thus amplifying chirality itself during the slow growth process (from 5 to 48 days to reach the plateau) [187].

Following the same ideas at the basis of the helical conformation of pyridine–pyridazine oligomers, naphthyridine–pyrimidine alternating units, as shown in Figure 17e, could also be used to favor a *transoid* arrangement of all the interheterocyclic bonds, and then obtain another lock-washer-shaped foldamer, as confirmed by NMR spectroscopy in CDCl_3_ and PXRD [189]. The naphthyridine–pyrimidine oligomers had an increased internal diameter (~3.8 Å) with respect to the previously reported pyridine–pyridazine tridecamer (~2.6 Å) [185], and then they were able to accommodate cations larger than protons (e.g., K^+^ and ammonium cations). NMR experiments and TEM images revealed that the foldamers with *n*-butyl side chains electrostatically bound different cations, due to the large dipoles of naphthyridine units, and then self-assembled in fibers in various solvent mixtures. In addition, the rational choice of chiral oligoammonium guests enhanced the self-assembly of fibers and drove the formation of chiral structures, as also confirmed via CD spectra.

NMR experiments followed by CD and UV–vis absorption spectra allowed us to deduce that two different kinds of helices could be obtained by allosteric regulation of polymeric foldamers in Figure 17f (average molecular weight ~1.7 × 10^4^) [190]. Those foldamers could assume either the innate folding, due to the extended (W-shaped) conformation of their pyridine–bacylhydrazone linker, or an alternative helical structure, when pentadentate Ag^+^ coordination caused the formation of the contracted (U-shaped) conformation, as shown in Figure 17g [191]. In the absence of silver ions, intra- and intermolecular hydrogen bonding stabilized the supramolecular polymer in a 9:1 chloroform/dimethylsulfoxide mixture. AFM analyses highlighted that the first nanofibers formed, which could be either tubular or rod-like superstructures, underwent a higher-order self-assembly to give parallel bundles. In the same solvent mixture, titration with AgBF_4_ clearly showed the highly cooperative sequential binding of silver cations and led to the formation of the corresponding fibers and bundles. Surprisingly, these morphologies were similar to those generated in the absence of added cations, despite the striking difference between the ellipsoid helix secondary structure (without Ag^+^) and the much more cylindrical helix obtained with Ag^+^.

### 3.3. Challenges in Design and Structural Characterization of Self-Assembled Aromatic Foldamers

As for the case of β-peptide foldamers—and for the same reasons—the design of self-assembling aromatic foldamers has already reached many outstanding results for all the assembled structures reported here. However, it must also be emphasized that, in this case, there are no examples of foldectures created in nonequilibrium conditions, as well as other supramolecular structures, such as nanosheets and nanotubes, and that self-assemblies of discrete numbers of aromatic foldamers in aqueous solution are heavily underrepresented. In addition, for aromatic foldamers, studies devoted to biological applications, or materials, are still at an embryonal stage, compared, for example, to β-peptides and peptoids.

The unresolved challenges from the point of view of an in-depth structural characterization are quite different among the various self-assembled structures. In the case of interpenetrating double (and multiple) helices in solution, there are no particular difficulties, because the comparison between diffraction data on crystals and NMR data in solution is usually sufficient to thoroughly elucidate structure, stability and kinetics of formation. Similar observations apply to self-assembled monolayers, for which both the secondary structure of single molecules and the intermolecular arrangement within the monolayers can be easily ascertained. Additionally, their electronic behavior can be studied by exploiting common techniques.

For vesicles, only rarely has the measurement of wall thickness been coupled to a good in silico approach, so that a molecularly precise and reliable description of vesicle membranes is still missing in many cases, and only qualitative drawings are reported.

The structural investigation with the atomic precision of fibers from aromatic foldamers is a challenge only partially addressed because it has usually taken advantage of different techniques furnishing indirect information (e.g., NMR, CD, DLS), but in only a limited number of cases has the much more reliable fit to PXRD data been attempted so far.

## 4. Foldamers Based on Other Building Blocks

The secondary structures of foldamers based on higher homologues of α-amino acids (e.g., γ- and δ-amino acids), and the rationale at the basis of their formation and stability, have not been studied at the same level of detail than those of β-peptides [1,2,3,4,5,6,7,8,9] and aromatic foldamers [2,6,10,11,12,18,19]. The same usually applies to the knowledge and comprehension of the folding propensities of foldamers based on peptide analogs [15] and oligomers with heterogeneous backbones, either in the sense of peptidic skeletons featuring different types of aliphatic amino acids [16] or mixed aliphatic–aromatic structures [17]. In addition, intramolecular arrangements of many particular classes of foldamers have often been studied by a single research group. As a logical consequence, even the capability to control their intermolecular interactions—that is their self-assembly—is somewhat limited in comparison to the examples reported in the previous sections. The most relevant exceptions are peptoids and α-aminoisobutyric acid-rich oligomers, for which only representative examples are reported here, as explained in the Introduction, and mixed α/β-peptides [16].

### 4.1. Discrete Self-Assembly in Solution: Up to Bundles

#### 4.1.1. Duplexes, β-Sheets, and Double Helices

##### Aib Oligomers in Chloroform

Head-to-tail self-association in CDCl_3_, due to intermolecular hydrogen bonds between the N-terminal NH and C-terminal CO functionalities, was revealed by variable-concentration NMR experiments on relatively short oligomers of α-aminoisobutyric acid (Aib) [192]. The two interacting 3_10_-helices were stabilized by the presence of Aib with respect to proteinogenic α-amino acids [193]. The type of N- and C-terminal groups, and their related H-bonding propensities, were found to influence self-association (e.g., N-terminal acetylation gave larger dimerization constants than carboxybenzyl and azido groups), with a generally higher impact for the N-terminus. In addition, longer oligomers aggregated more strongly than shorter analogs with the same termini, probably due to the generation of larger macrodipoles. These in turn came from the greater number of intramolecularly H-bonded amide groups present in the more stable 3_10_-helices formed by longer peptides.

##### Mixed Aliphatic/Aromatic Foldamers in Aqueous Solution

Different kinds of dimers in solution have been obtained so far. Stable hetero-duplexes could be formed by aqueous self-assembly when oligomers of electron-rich DAN (1,5-dialkoxy-naphthalene) units, linked by freely rotatable linkers also including negatively charged α-amino acids, were dissolved together with oligomers of electron-deficient NDI (1,4,5,8-naphthalene-tetracarboxylic diimide) units [194,195]. The high stability of longer duplexes was qualitatively evaluated by polyacrylamide gel electrophoresis (PAGE) experiments, and more quantitatively by NMR and isothermal titration calorimetry (ITC). The expected hydrophobic effect, but also the electrostatic complementarity between the face-centered π-stacked DAN-NDI units, were the driving forces for the enthalpically-driven aqueous self-assembly, whose strength increased in a roughly proportional way with the chain lengths.

##### Mixed Aliphatic/Aromatic Foldamers in Chloroform

**Figure 18:**
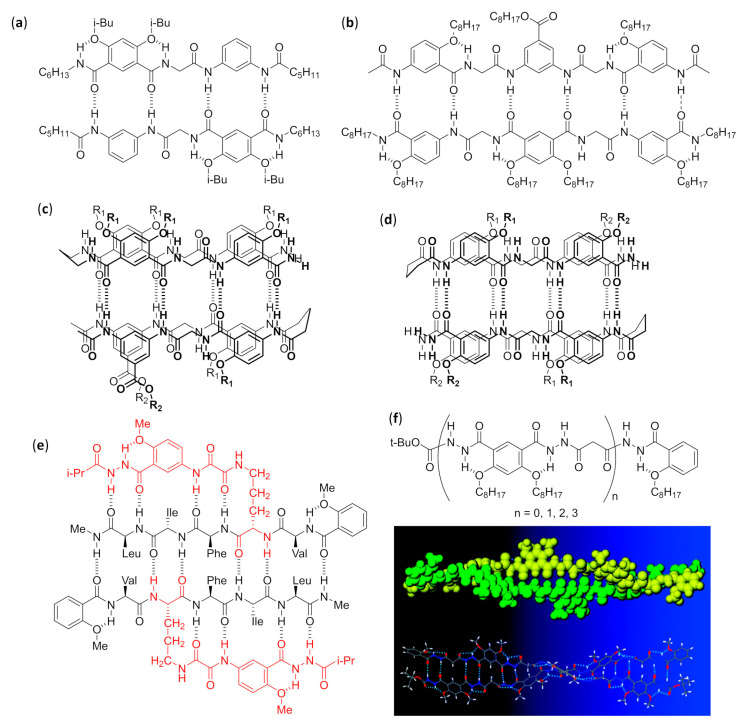
(**a**) Quadruply and (**b**) sixtuply intermolecularly hydrogen bonded dimers (Refs. [196,197,198,199,200]); (**c**) Heteroduplex and (**d**) homoduplex of foldamers whose linear strands are not complementary (Ref. [201]); (**e**) An example of self-recognizing β-sheets based on the Orn(*i*-PrCO-Hao) sequence, highlighted in red (Refs. [202,203,204,205]); (**f**) Flexible aromatic hydrazide-malonic acid alternating foldamers, together with the molecular mechanics-computed structure (n = 3, antiparallel strands are color-coded in the space-filling structure, while those in both intra- and interstrand H-bonds are shown as dotted lines in the stick representation) (adapted with permission from Ref. [206]).

Gong’s group used glycine as a linker between aromatic residues derived from 3-aminobenzoic acid, 1,3-benzenedicarboxylic acid (isophthalic acid), and 1,3-diaminobenzene (1,3-phenylenediamine), obtaining oligoamides with various sequences of hydrogen bonding sites that could give rise to either complementary or self-complementary pairings. The resulting molecular strands formed duplexes in anhydrous CDCl_3_ via intermolecular hydrogen bonding interactions between the backbone amide O and H atoms, and the NMR-derived dimerization constants—from ~10^4^ M^−1^, in Figure 18a, to ~10^9^ M^−1^, in Figure 18b—raised with the number of intermolecular hydrogen bonds (from four to six) [196,197,198]. The exceptional stability, further confirmed by isothermal titration microcalorimetry and thin-layer chromatography (TLC), was explained by positive cooperativity among the numerous hydrogen bonding and van der Waals interactions, as well as the preorganization of individual strands by intramolecular hydrogen bonds. In addition, when the hydrogen bonding units were connected with lengthened flexible linkers, the association constant decreased, while using rigid aromatic linkers led to pairings with comparable stability, because the bent conformation was counterbalanced by the π-stacking between the linkers themselves [199]. A quadruply H-bonded duplex was further employed as an inducer for the formation of an antiparallel β-sheet between two tripeptides that were bonded at the C- and N-termini, respectively. It is worth mentioning that, in the absence of the duplex, no interaction between the peptidic chains was observed [200].

In a following study, an extensive NMR analysis demonstrated that covalently linking—in an apparently wrong way—two quadruply H-bonding strands with a flexible three-methylene unit might still lead to extremely stable heterodimers, as shown in Figure 18c, or homodimers, as shown in Figure 18d, in CDCl_3_ solution [201]. In fact, such oligomers contained sequences of eight hydrogen bonding donor/acceptor functionalities that could neither pair nor self-pair as linear strands. However, antiparallel duplexes were formed between two folded strands, in which the two halves of each strand were intramolecularly π-stacked and formed a correct pattern of intermolecular hydrogen bonds, as shown in Figure 18c,d. This likely occurred by an aspecific initial assembly, followed by a stepwise rearrangement that rapidly led to the formation of all the correct sequence-specific intermolecular H-bonds within the two intramolecularly π-stacked strands. Thus, in this case, the assembly promoted the folding, and not the contrary.

Similar strategies, exploiting rationally designed sequences of aromatic and proteinogenic α-amino acids in order to mimic the hydrogen bonding functionality of a peptide β-strand, have also been reported by Nowick’s group, leading to mixed foldamers that were dimerized by β-sheet formation in CDCl_3_ [202,203,204,205]. The short sequence Orn(*i*-PrCO-Hao), highlighted in red in Figure 18e, [203], refers to an ornithine in which the δ nitrogen atom is substituted with a *i*-PrCO-Hao fragment composed of an isobutyryl hydrazido derivative of 5-amino-2-methoxybenzoic acid, whose amino group is, in turn, derivatized with oxalic acid.

When incorporated into a suitable peptide sequence, Orn(*i*-PrCO-Hao) led to the intramolecular formation of a stable ten-membered hydrogen bonded pseudocycle with the amino group of the following residue, reminiscent of a β-turn in a β-hairpin. Apart from the usual concentration-dependent chemical shift variation and comparison with ^1^H NMR spectra in competitive solvents, transverse-ROESY (Tr-ROESY) experiments, and molecular dynamics simulations were also used to precisely deduce the structures of β-sheet dimers formed in solution. Both homodimerization, as shown in Figure 18e, and preferential heterodimerization could be evidenced in form of the expected β-sheet-like structures, according to the choice of α-amino acids, thus confirming the possibility for rigorous self- and hetero-molecular recognition [204,205]. Double helical duplexes could be found, using IR and either 1D or 2D NMR data coupled to molecular mechanics minimizations for the self-pairing in apolar solvents of longer oligomers of flexible hydrazide derivatives, reported in Figure 18f, in which the easily rotatable bonds came from malonic acid units [206].

**Figure 19:**
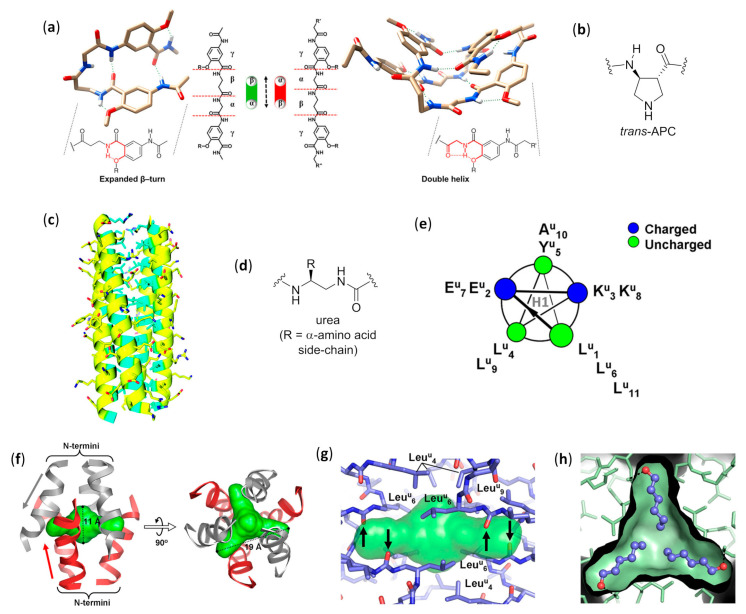
(**a**) Different behavior of γβαγ and γαβγ foldamers (adapted with permission from Ref. [207]); (**b**) Structure of *trans*-APC (*trans*-β-aminopyrrolidinecarboxylic acid) (Refs. [2,4,5,8]); (**c**) Tetrameric bundle of a foldamer bearing only hydrophobic β^3^-amino acids at *a* and *d* positions (yellow: α-residues; cyan: β-residues) (adapted with permission from Ref. [213]); (**d**) Structure of generic urea monomers with proteinaceous side chains (Refs. [8,217,218,219,220]); (**e**) Helical-wheel representation of urea Leu^u^-Glu^u^-Lys^u^-Leu^u^-Tyr^u^-Leu^u^-Glu^u^-Lys^u^-Leu^u^-Ala^u^-Leu^u^ (L^u^ = Leu^u^, E^u^ = Glu^u^, K^u^ = Lys^u^, Y^u^ = Tyr^u^, A^u^ = Ala^u^) (reprinted with permission from Ref. [220]); (**f**) Hexameric bundle and hydrophobic cavity (green) of urea in panel e (3-fold symmetry, the gray and red arrows indicate the N- to C-terminus direction for the three molecules belonging to the upper and lower portions, respectively) (reprinted with permission from Ref. [220]); (**g**) Detail of the hydrophobic cavity (green) within the crystal structure in panel (**f**) (black arrows indicate the carbonyl groups of Leu^u^_6_ residues, which act as hydrogen bonding acceptors) (adapted with permission from Ref. [220]); (**h**) Detail of the hydrophobic cavity of the same oligourea in the crystal structure with three 1-hexanol molecules bound to carbonyl groups of Leu^u^_6_ residues within the hydrophobic cavity (hydrogen bonds are shown as black dotted lines) (adapted with permission from Ref. [220]).

Short tetrapeptides with two aromatic γ-amino acid residues at both termini and either the βα or the αβ central sequence (α = Gly and β = β-Ala, 3-aminopropionic acid) were synthesized to form self-pairing β-strands that gave rise to linear H-bonded duplexes [207]. Structural determinations were carried out by means of a complete set of NMR data in CDCl_3_—DMSO-*d*_6_ titrations, concentration- and temperature-dependent experiments, 2D NOESY—and mass spectrometry with both electrospray ionization (ESI-MS) and traveling wave ion mobility (TWIM-MS) techniques. Peptides including the two sequences displayed different self-assemblies, as shown in Figure 19a, which were corroborated by density functional theory (DFT) analysis, taking into consideration long-range dispersion correction. Whereas achiral γβαγ tetrapeptides formed expanded β-turns, the extended helices adopted by their γαβγ sequence isomers dimerized into double helices, stabilized by intermolecular hydrogen bonds and π-stackings. On the other hand, the introduction of a chiral amino acid (i.e., L-alanine, L-Ala, or L-β-homoalanine, L-β-hAla, in the place of Gly or β-Ala, respectively) was shown to improve the double helix stability, even in polar solvents, and furnish a dominant helical sense with a concomitant reduction in the helix inversion process.

Double helices were also obtained from short homooligomers of achiral γ-amino acids, either α,β-unsaturated [208] or aromatic [209] in their nature. In the case of α,β-unsaturated γ-peptides, the self-assembled β-double helices, evidenced by X-ray diffraction, were simply stabilized by intermolecular hydrogen bonds, and their structures were also stable in chloroform, as ascertained by extensive NMR and fluorescence analyses [208]. On the other hand, the same experimental techniques demonstrated that the self-association of short aromatic γ-peptides containing *m*-aminobenzoic acid and *N*,*N*’-dicyclohexylurea was highly solvent-dependent. In particular, starting from the same single helical conformation, sheet-like structures were formed in methanol by equilibration with the extended structure, while in chloroform two molecules intertwined because of two intermolecular H-bonds [209].

##### Mixed α/γ Aliphatic Foldamers in Chloroform

Well-constructed cyclic peptides made of alternating α-amino acids (α-AA) and *cis*-3-aminocyclohexanecarboxylic acid (*cis*-γ-ACHC) in a 1:1 ratio (α,γ-CPs) behaved as with all-α analogues, the cyclic *D*,*L*-α-peptides (α-CPs), and usually formed insoluble peptide nanotubes, by extended hydrogen bonding-driven self-association (SPNs, self-assembling peptide nanotubes) [210]. However, SPNs formation was avoided for cyclic sequences of tetrads composed of 3 α-AA and one *cis*-γ-ACHC with properly chosen absolute configurations (e.g., the cyclic heterooctamer *cyclo*-[(*L*-γ-ACHC-*D*-α-AA-*L*-α-AA-*D*-α-AA)_2_-]), in which hydrogen bond donors (NH groups) lying on one CP face were eliminated by methylation. In those cases, X-ray crystallography, NMR experiments, FT-IR data of diagnostic amide bands, and mass spectroscopy confirmed that the self-association was limited to β-band dimers with a β-sheet-like hydrogen bonding pattern and the expected all-equatorial arrangement of substituents [210].

#### 4.1.2. Bundles in Aqueous Solution

##### Mixed α/β Foldamers

Beyond the already reported bundles formed by β-peptides, the Gellman’s group also studied the self-assembly of foldamers with various heterogeneous α/β backbones [211,212,213,214,215]. The rationally designed sequences of α- and β^3^-amino acids had repeating heptads *abcdefg* as in the α-helix of an engineered variant of the yeast transcription factor, GCN4-pLI, which was known to exploit the leucine zipper mechanism to form tetrameric bundles in aqueous solution [216], and their association above given concentrations was measured by analytical ultracentrifugation (AU) [211]. Within the heptads, the polar or charged β^3^ residues covered one helix side, being stacked in positions *b* and *f*, while the hydrophobic α-amino acids occupied the adjacent *a* and *d* positions. This led to the expected segregation of lipophilic side chains in the core of trimeric or tetrameric coiled-coil bundles, depending on the choice of lipophilic α residues in *a* and *d* positions (i.e., Val in *a* and Leu in *d* gave trimers, while Leu in *a* and Ile in *d* gave tetramers), whereas the periphery was characterized by stabilizing interactions among polar side chains. According to AU data, tetrameric bundles could also be obtained in aqueous solution by exploiting the opposite approach—that is, using β^3^-amino acids bearing only hydrophobic side chains in the *a* and *d* positions, as shown in Figure 19c [213]. Interestingly, in the crystal state, the antiparallel rectangular arrangement of the four foldamers highlighted a peculiar hydrophobic packing of β^3^-hLeu and β^3^-hIle, quite different from the known “knobs-into-holes” (KIH) of the same side chains in α-amino acids of GCN4-pLI, and with the possible additional stabilization of up to 20 C^α^-H···O=C unconventional hydrogen bonds per tetramer among β^3^ residues.

In the attempt to better mimic the side chains distribution of the α-helical peptide GCN4-pLI, other mixed α/β backbones were also examined, namely ααβαααβ, αααβ, ααβ, ααβαβαβ, and αβ, in which β-residues could be either linear or cyclic, apolar or positively charged. [215]. Surprisingly, they found that all the α/β-backbones were suitable to fold and then self-associate in bundles, but the ααβαααβ, αααβ, and ααβ repeat patterns gave the best mimicry of the GCN4-pLI quaternary structure in crystals. In addition, the sequential replacement of α residues with acyclic β^3^-amino acids, characterized by higher torsional freedom, caused an entropically-driven progressive loss in helical content and self-association ability, which could be deduced by the cooperative thermal unfolding detected by AU and variable-temperature CD experiments. The thermal stability could be restored using either cyclic *trans*-ACPC (*trans*-2-aminocyclopentanecarboxylic acid), as shown in Figure 8a, or its positively charged congener *trans*-APC (*trans*-β-aminopyrrolidinecarboxylic acid), as shown in Figure 19b, instead of apolar or charged β^3^-amino acids, respectively.

The folding and assembling propensities of ααβ-foldamers were further investigated in more detail, evidencing a general increase in helix and tetrameric bundle stabilities caused by the successive β^3^→cyclic replacements [214]. However, somewhat variable effects on the propensity to form bundles in solution were observed, exploiting again sedimentation equilibrium AU and a high-temperature-induced decrease in CD ellipticity. In fact, even if the change β^3^→cyclic usually gave more stable tetrameric bundles, X-ray data showed that in a few cases the inserted constrained cyclic β-amino acid generated steric repulsions in the assembled state, thus cancelling the benefits of an enhanced helical propensity and leading to either a decreased bundle stability or a different stoichiometry (e.g., trimeric or pentameric bundles).

In a slightly different study, the possibility for bundles formation among α-peptides and alternating 1:1 α/β-peptides containing either linear β^3^, β^2^ or cyclic five-membered β-amino acids was evaluated [212]. The 14/15-helices obtained from alternating 1:1 α/β-peptides had heptad repeats with side chain disposition mimicking those of α-helices, and the judicious placement of polar/charged and apolar side chains allowed us to obtain 2:2 heterotetrameric bundles with all-α peptides. The stability in PBS was due to the formation of a purely hydrophobic core and an exterior surface with polar and charged groups forming electrostatic interactions.

##### Urea Foldamers

The first non-peptidic bundles in solution were achieved by Guichard’s group, that exploited the stable helices with 2.5 residues per turn assumed by oligoureas with proteinaceous side chains, as shown in Figure 19d, characterized by the 12- and 14-membered H-bonded pseudocycles formed by bifurcated hydrogen bonds between C=O(*i*) and N’H(*i*-2) and NH(*i*-3) [217,218,219,220]. The undecamer Leu^u^-Glu^u^-Lys^u^-Leu^u^-Tyr^u^-Leu^u^-Glu^u^-Lys^u^-Leu^u^-Ala^u^-Leu^u^ contained charged residues in positions 2, 3, 7, and 8 (i.e., in two contiguous positions of the pentad repeats), as shown in Figure 19e, and formed a hexameric bundle in aqueous solution of sufficient concentration, as verified by both ESI-MS data and an increase in molar residual ellipticity in variable-concentration CD experiments. X-ray single-crystal diffraction data showed that the hexameric bundle was composed of two trimeric arrangements with opposite N→C directions, as shown in Figure 19f, and NOESY spectra confirmed that this arrangement was also present in solution [217,220]. The hydrophobic Leu^u^ portions were buried within a three-fold symmetry hydrophobic cavity and made an extensive knobs-into-holes (KIH) packing, while the hydrophilic (Glu^u^, Lys^u^), and also the slightly lipophilic (Ala^u^ and Tyr^u^) residues, were exposed to the aqueous environment. Within the three separate tunnels of the hydrophobic cavity in each bundle, it was possible to encapsulate three molecules of different alcohols (i.e., isopropyl alcohol, 1-butanol, 1-pentanol, 1-hexanol, 2-ethoxyethanol, 2-propoxyethanol, and 1,4-butanediol), whose hydroxyls were H-bonded to urea carbonyls of Leu^u^_6_ residues, as shown in Figure 19g,h. It is noteworthy that NMR titration with different alcohol stoichiometries showed that encapsulation also occurred in aqueous solution. In addition, a higher occupancy of the cavity volume gave rise to a noticeable increase in bundle stability, as measured by the thermal melting profiles obtained through variable-temperature CD spectra [220].

The complete NMR, ESI-MS, CD and X-ray studies carried out on urea foldamers bearing point mutations further demonstrated that the bundle of this short oligourea did not tolerate the substitution of any Leu^u^ participating with KIH interactions with nonhydrophobic residues (e.g., Asn^u^). However, systematic substitution with valine residues (Val^u^) could still lead to hexameric bundles, even though the resulting thermal stability in solution depended, in a subtle—and sometimes unpredictable—way, on the exact positioning, especially for the two terminal residues (Leu^u^_1_ and Leu^u^_11_) [218]. Eventually, the introduction of two iodine atoms at the *orto* positions, with respect to hydroxyl groups of every Tyr^u^_5_ residue exposed to the aqueous environment, allowed us to solve the crystal structure by single-wavelength anomalous diffraction (SAD) methods, and also showed that the oligourea hexameric helix bundle was able to accommodate relatively large chemical modifications on its outer surface [219].

### 4.2. Extended Self-Assembly: Monolayers, Vesicles, and Fibers

#### 4.2.1. Self-Assembled Monolayers

##### Mixed α/β Foldamers at the Air–Water Interface

**Figure 20:**
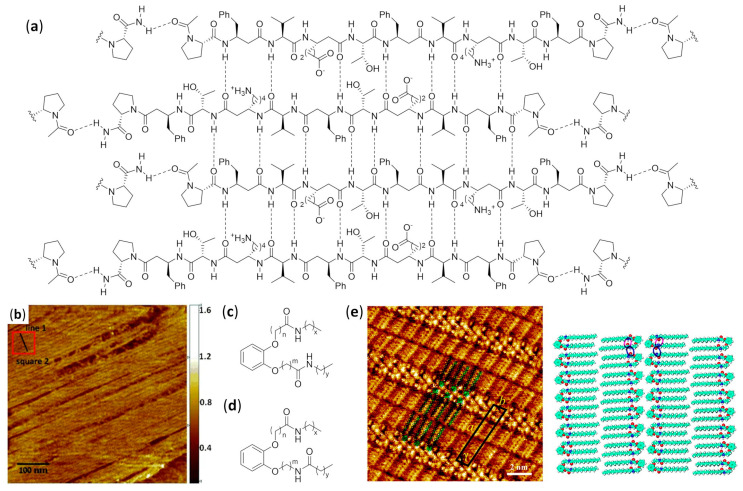
(**a**) Two-dimensional antiparallel arrangement of the amphiphilic oligomer βEβK within the monolayers at the air–water interface (hydrophilic side chains project into the aqueous phase below the plane, while hydrophobic side chains project into the air above the plane) (Refs. [221,222,224]); (**b**) AFM image of βEβK film transferred at 2.6 mN m^−1^ onto mica (reprinted with permission from Ref. [224]; (**c**,**d**): general structures of catechol foldamers (Refs. [225,226,227,228]); (**e**) STM image of a physisorbed catechol derivative (n = 1, m = 3, x = 11, y = 12 in panel (**d**)) at the 1-phenyloctane/HOPG interface (unit cell in black), and molecular model of its 2D pattern (intramolecular and intermolecular hydrogen bonds indicated by red and blue circles, respectively) (reprinted with permission from Ref. [227]).

Two undecameric α/β-peptides with a 1:1 alternating pattern were designed to form stable pleated sheets at the air–water interface by interstrand hydrogen bonding in an antiparallel alignment [221]. The two α/β-peptides only contained branched α-amino acids, that were shown to favor the formation of extended conformations [222] and differentiated from each other by a simple inversion in the positioning of β^3^-amino acids bearing charged side chains. Indeed, Ac-Pro-β^3^-hPhe-Val-β^3^-hLys-Thr-β^3^-hPhe-Val-β^3^-hGlu-Thr-β^3^-hPhe-Pro-NH_2_, indicated as βKβE, had β^3^-hLys at position 4 and β^3^-hGlu at position 8, while βEβK, Ac-Pro-β^3^-hPhe-Val-β^3^-hGlu-Thr-β^3^-hPhe-Val-β^3^-hLys-Thr-β^3^-hPhe-Pro-NH_2_, had β^3^-hGlu at position 4 and β^3^-hLys at position 8). Within the intended self-assembled structure, all the hydrophobic side chains were pointing toward the air, while the hydrophilic ones were projected into the water, as shown in Figure 20a. The analysis of grazing incidence X-ray diffraction (GIXD) data sets evidenced great differences in the extensions of formed monolayers, which were indicative of the greater stability of linear strands of foldamer βEβK. This was due to more favorable interactions of charged side chains of β^3^-hGlu and β^3^-hLys units in βEβK with the backbone macrodipole [221], with respect to those in the positional isomer βKβE and in any analogous α-peptidic monolayer [223]. By using atomic force microscopy, monolayers of the βEβK foldamer were subsequently shown to be composed of nanometer-width fibers, ordered in differently oriented domains in a globally disordered material, when transferred at low surface pressure, while micrometer-length uniformly aligned fibers were obtained transferring the foldamer at higher surface pressure, as shown in Figure 20b [224]. In addition, when two β^3^-hLys residues (foldamer βK_2_) or two β^3^-hGlu residues (foldamer βE_2_) were inserted at both positions 4 and 8, different propensities to form pleated sheets were evidenced by surface pressure-area isotherms. Whereas the βK_2_ foldamer only self-assembled in aqueous 0.1 M KCl, which reduced the otherwise destabilizing repulsions between the protonated β^3^-hLys side chains, βE_2_ formed stable monolayers, even in deionized water. This was due to an increase of about 1.5 units in p*K*_a_ of β^3^-hGlu side chains, which, in turn, became able to form stabilizing intermolecular H-bonds between carboxylic acid moieties. However, as deduced by means of attenuated total reflection FT-IR spectroscopy (ATR FT-IR), the rational design bestowed foldamers with the ability to self-assemble to some extent, even in the chloroform/trifluoroacetic acid 9:1 stock solutions [224].

##### Catechol-Based Foldamers at Different Interfaces

Van Esch’s and De Feyter’s groups studied, by high-resolution scanning tunneling microscopy (HR-STM), various self-assembled monolayers (SAMs) of foldamers based on catechol, mostly at interfaces between a suitable liquid and a conductive and atomically flat surface (e.g., highly oriented pyrolytic graphite, HOPG, or gold), in which catechol acted as β-turn mimic for linear alkyl chains interrupted by two rationally positioned amide groups, as shown in Figure 20c [225,226,227,228]. Compounds with different spacers lengths (n, m) and aliphatic chains with twelve carbons (x = y = 11), as shown in Figure 20c, were first submitted to systematic conformational searches in 2D conformational space [225]. As a result, the best folding propensity for the intramolecular hydrogen bonding in a completely flat structure was found to be for n = m + 1 or n = m + 3, leading to the most stable all-*trans* arrangement of both spacers. The monolayers formed at the 1-octanol/HOPG interface in all cases, but with different folding and levels of order in the SAMs themselves, and the catechol derivative with n = 6 and m = 3, exhibited the best-ordered 2D structures. These were characterized by STM as having the theoretically forecasted folding of the intramolecularly H-bonded molecules with both chains in the minimum energy conformation. The perfect alignment in intermolecularly H-bonded parallel rows evidenced head-to-head contacts among catechol moieties and tail-to-tail interactions among methyl groups.

When a catechol derivative with n = m = 3, x = 11, and an “inverted” amide group (i.e., the carbonyl carbon and not the nitrogen atom bore the 13 carbons alkyl chain, y = 12), as shown in Figure 20d, was deposited at water/Au(111) and 1-octanol/graphite interfaces, the solvent effects on both the intramolecular folding and the SAMs formation were evaluated [226]. Deposition from 1-octanol evidenced that such a solvent was not able to guarantee the achievement of thermodynamic equilibrium, because diverse monolayers of unfolded molecules were obtained within the same sample. Moreover, in some monolayers there were molecules with only one chain adsorbed onto graphite. Indeed, this also indicated that molecular modeling had a limited reliability in that case, due to both the importance of kinetics in monolayer formation and the neglected solvent effects on intra- and intermolecular interactions. Deposition on gold from water led to much more stable monolayers with both lipophilic chains adsorbed onto the surface, very likely due to hydrophobic effect, and some 2D head-to-head structures of intramolecularly folded molecules could be detected, but still observed lamellar polymorphism. Starting from a saturated 1-phenyloctane solution gave, again, lamellar polymorphism without in-plane folding for the same compound onto HOPG, also highlighting that only one alkyl chain was adsorbed onto the graphite surface [227]. The same occurred for a compound with a longer spacer (n = 5), as shown in Figure 20d, while only a foldamer with n = 1 formed stable and uniform molecular patterns at the 1-phenyloctane/HOPG interface, as shown in Figure 20e. The minimum energy conformation of foldamers was stabilized by intramolecular hydrogen bonding and extensive van der Waals interactions, and the same driving forces were at the basis of the alignment within lamellae, with head-to-head contacts among catechols and tail-to-tail interactions among chain-ending methyl groups, as shown in Figure 20e [227].

As a last step in the study of these surface-confined catechol bis-amides, the authors applied the same design (n = 1, m = 3) to extended sequences with from two to four catechol turn units and decamethylenic interunit spacers, as well as different alkyl chains at both ends, exploiting saturated 1-octanol solutions deposited on HOPG [228]. Not in all cases, the HR-STM analysis evidenced the adsorption of folded conformations and, even when folded conformations predominated, sometimes large defects contaminated the 2D patterns. However, the most complex foldamer gave rise to quite disordered self-assembled monolayers, but upon heating and addition of 1-phenyloctane, these changed to ordered lamellar structures, whose arrangement was reminiscent of those previously obtained with simpler molecules [225,227].

##### Urea Foldamers on Gold

SAMs on gold were easily obtained using 2.5-helices of short oligoureas (two, four, and six residues) based on Ala^u^ and Leu^u^, as shown in Figure 19d, derivatized at either the N- (the δ+ pole of the helix macrodipole) or the C-terminus (the δ- pole) with a thiol group, and evaluated as electron transfer mediators by means of current sensing atomic force microscopy (CS-AFM) [229]. All ureas were ascertained to assume the 2.5-helix conformation both in solution and in the solid state, according to the NMR, CD, and FT-IR characterizations, and evidenced the expected decrease in conductance as the chain length increased. Due to the reduced width of the barrier for such short oligomers, the tunneling was very likely the prevailing mechanism. In addition, the barrier height—which is related to structural features, such as the secondary structure and its stability [230]—was very similar for both sets of oligomers, regardless of the thiol attachment point (N- or C-terminus). Longer oligoureas (from 8 to 12 residues) were further investigated with CD, FT-IR, and PM IRRAS (polarization modulation infrared reflection–absorption spectroscopy) measurements, and their electronic behavior was evaluated by CS-AFM. From these experiments, the mechanistic dependence of electron transport on the SAM thickness was evident, the hopping mechanism becoming dominant as the length increased [231].

#### 4.2.2. Nanosheets

##### Peptoids in Aqueous Solutions

**Figure 21:**
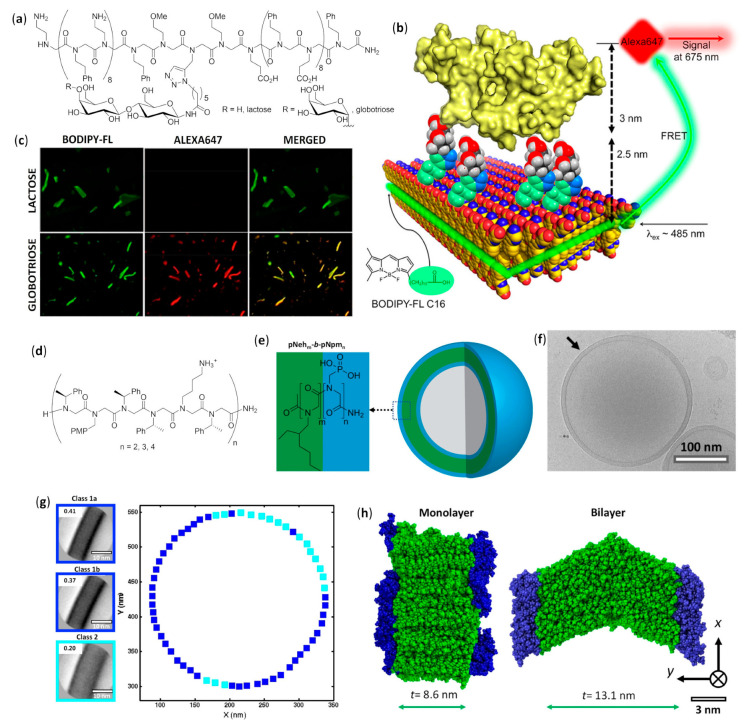
(**a**) Structures of peptoids bearing lactose and globotriose ligands (Ref. [240]); (**b**) Molecular model of lectins (yellow molecular surface) binding to peptoid nanosheets displaying carbohydrate ligands and FRET-based assay—BODIPY-FL C16 forms a layer between the two peptoids layers, which are arranged in parallel rows and display carbohydrates on the surface (unnecessary hydrogens omitted for clarity, carbons are in yellow, oxygens in red, and nitrogens in blue; lectin represented as yellow molecular surface); binding of lectins is detected by the emission of Alexa647 after FRET between BODIPY-FL C16 and Alexa647 (adapted with permission from Ref. [240]); (**c**) Fluorescence images of peptoid nanosheets functionalized with lactose and globotriose after incubation with Alexa647-conjugated Stx1B, showing the selective binding to the globotriose-functionalized peptoid nanosheet (adapted with permission from Ref. [240]); (**d**) Example of microsphere-forming peptoids (PMP = *p*-methoxyphenyl) (Refs. [241,242,243]); (**e**) General structure of amphiphilic di-block polypeptoids, with the hydrophilic (blue) and hydrophobic (green) blocks, and vesicle formed (adapted with permission from Ref. [244]); (**f**) Cryo-EM micrograph of pNeh_26_-*b*-pNpm_10_ vesicles (the arrow indicates the vesicle analyzed in panel (**g**)) (adapted with permission from Ref. [244]); (**g**) Averaged cryo-EM micrographs of boxes extracted from the vesicle in panel (**f**), formed by pNeh_26_-*b*-pNpm_10_, with the classification according to the thickness in that membrane portion (classes 1a and 1b, both grouped in class 1, are ~10 nm; class 2 is ~12 nm). The probability of finding each class in the vesicle is given in the related boxes, and the disposition of class 1 (dark blue) and class 2 (cyan) boxes in the membrane of the vesicle in panel (**f**) is also shown (adapted with permission from Ref. [244]); (**h**) Relaxed simulation of monolayer (left, interdigitated hydrophobic chains) and bilayer membranes (right, non-interdigitated hydrophobic chains), with the corresponding hydrophobic core thickness, *t* (peptoid blocks color-coded as in panel (**e**), side chains and water molecules omitted for clarity) (adapted with permission from Ref. [244]).

Zuckermann’s group demonstrated that rationally designed amphiphilic peptoids containing functionalized loops were useful as scaffolds for the recognition of relevant biological macromolecules in aqueous environments [232,233,234,235]. Their hierarchical self-assembly, consisting in the preliminary formation of monolayers at the air–water interface, followed by lateral compression, led to bilayers (nanosheets) in which the polar and charged groups were oriented toward the aqueous phase, while the hydrophobic portions became buried into the interior [236]. As ascertained by means of AFM, X-ray powder diffraction and molecular dynamics simulations, the peptoid backbone assumed the peculiar Σ-strand motif, in which the overall linear secondary structure was due to the opposed dihedral angles adopted by adjacent monomers [237], with the exception of amide bonds, which were always in the *cis* conformation [238,239]. As an example of molecular recognition, nanosheets formed in aqueous environment by differently glycosylated peptoids were first characterized by fluorescence microscopy, SEM, and PXRD, and then evaluated for their ability to selectively bind to multivalent carbohydrate-binding proteins, also called lectins, such as Concanavalin A, Wheat Germ Agglutinin, and the recognition domain (subunit B) of Shiga toxin type 1 (Stx1B) [240]. In particular, the peptoids in Figure 21a differentiated from each other only by the presence on the central peptoid unit of lactose or globotriose, the latter being the natural ligand of Stx1B. The peptoids featured two differently charged arms, made of alternating aromatic/hydrophobic (*N*-(2-phenylethyl)glycine, Npe) and negatively (*N*-(2-carboxyethyl)glycine, Nce) or positively charged (*N*-(2-aminoethyl)glycine, Nae) units. The interplay of salt bridges between residues with opposite charges, arranged in rows, hydrophobic interactions, and π-stacking caused the irreversible formation of stable nanosheets, while the loops generated by the presence of the three central polar uncharged residues (two *N*-(2-methoxyethyl)glycine residues, Nme, and the sugar-bearing monomer) led to the display of saccharide moieties on the exterior surface, as shown in Figure 21b.

The selective binding of Stx1B to only globotriose-conjugated nanosheets was evidenced by the colocalization—yellow color in Figure 21c—of fluorescence emissions of BODIPY-FL C16 (green) and Alexa647 (red) dyes, which were noncovalently incorporated into nanosheets and conjugated to Stx1B, respectively, as shown in Figure 21b. Then, the selective molecular recognition was confirmed by the efficiency of Förster resonance energy transfer (FRET), whose energy transfer from the donor to the acceptor chromophore through nonradiative dipole–dipole coupling is extremely sensitive to the interchromophoric distance, as shown in Figure 21b.

#### 4.2.3. Microspheres

##### Peptoids in Aqueous Solutions

Partially water soluble peptoid foldamers with a pronounced propensity to the formation of helices with three residues per turn were demonstrated to be useful for microsphere coatings, after dissolution in ethanol/water mixtures followed by drying [241,242,243]. In particular, the effects of helicity, ionic strength, temperature, and pH in various aqueous solutions were evaluated for peptoids made of the repeating hexameric units, reported in Figure 21d [243]. The secondary structure in solution was determined by CD, while microsphere morphology was analyzed by means of environmental scanning electron microscopy (ESEM) and additional information on stability and surface charge were obtained by differential scanning calorimetry (DSC) and ζ potential measurements by electrophoretic light scattering (ELS). The incorporation of *N*-((*S*)-1-phenylethyl)glycine (Nspe) residues on two faces of the helix was already shown to be essential to obtain the desired folding in solution and the stabilizing intermolecular π-stacking interactions in the self-assembled microspheres [241]. In addition, alternating ammonium and *p*-methoxybenzyl side chains on the third helix face gave a sufficient water solubility and endowed microspheres with a positive ζ potential, therefore favoring the attachment to the negatively charged surfaces of glass substrates. Due to inherently reduced helicity [50,243] and π-stacking ability, as well as excessive water solubility, the hexamer—n = 1 in Figure 21d—did not form microspheres. On the other hand, longer peptoids gave rise to microspheres whose average diameter decreased as the chain length increased (2.26 μm for the 12 mer, 1.91 μm for the 18 mer, and 1.24 μm for the 24 mer), which was interpreted as the result of a tighter packing, generated in turn by the increase in helicity along the series [243], even if the exact packing mode was not investigated. Phase transition temperatures, determined by DSC, were in the range 74–83 °C, and increased with the length, probably due, again, to increased helicity and π-stacking interactions. Microsphere coating were also demonstrated to be unstable in pure water and at pH < 7, but stable for prolonged periods in different non-acidic biological buffers.

#### 4.2.4. Vesicles

##### Peptoids in Aqueous Solutions

Vesicles in water could be obtained by slow evaporation of THF from THF/water 1:1 solutions of amphiphilic di-block polypeptoids poly-*N*-(2-ethyl)hexylglycine-*block*-poly-*N*-phosphonomethylglycine (pNeh-*b*-pNpm), with a fixed overall chain length (m + n = 36) and different proportions of hydrophobic and hydrophilic residues, as shown in Figure 21e [244]. All the copolymers formed vesicles, but the authors chose not to investigate them, exploiting radially averaged cryo-EM images, albeit this technique was demonstrated to be very useful to study vesicle morphology [245], due to the unavoidable loss of information about local differences in membrane thickness. Using procedures often employed in the field of protein structural investigation, vesicle micrographs were taken by low-dose cryogenic electron microscopy (cryo-EM)—the contrast being obtained by defocusing—and then sorted in segments of different classes and averaged, thus leading to high resolution images of local membrane thickness. Molecular dynamics simulations were further undertaken in order to find the different polypeptoid arrangements in local membrane sections belonging to different classes. As a result, vesicles formed by pNeh_26_-*b*-pNpm_10_, that had, at a first glance, a uniform membrane thickness, as shown in Figure 21f, after the sorting-averaging procedure, could instead be classified in six different classes. Among them, three classes accounted for only 2%, while the remaining classes 1a and 1b (78%, taken collectively as class 1) and class 2 (20%), as shown in Figure 21g, constituted almost completely the membrane and were usually grouped in membrane portions featuring only one class. Very interestingly, the striking difference in thickness between classes 1 and 2 could be reproduced by MD simulations, which confirmed the coexistence of monolayered and bilayered membrane segments, characterized by interdigitated and non-interdigitated hydrophobic chains, respectively, as shown in Figure 21h.

##### Mixed Aliphatic–Aromatic Foldamers in Methanolic Solutions

**Figure 22:**
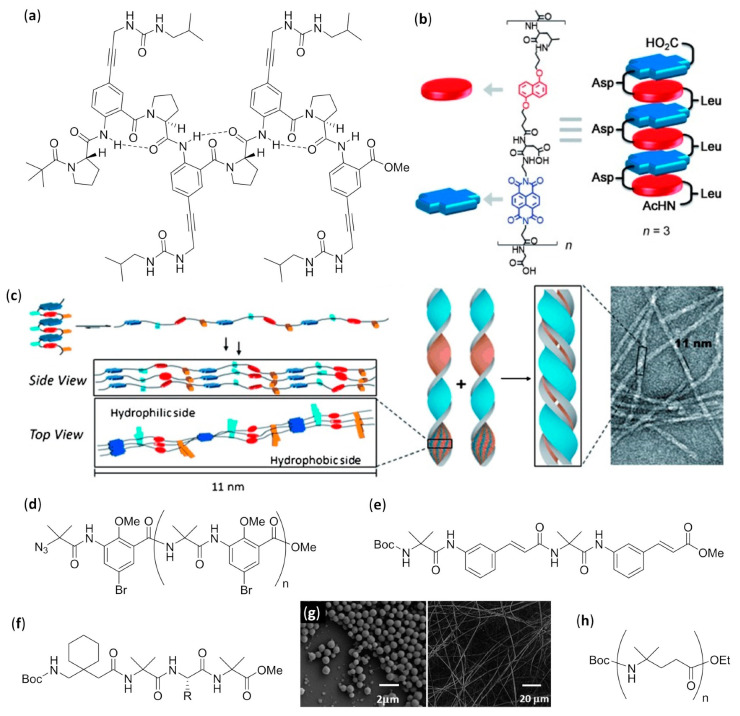
(**a**) Structure of Piv-(Pro-Ant)_4_-OMe foldamer with the hydrogen bonding pattern (Refs. [246,247]); (**b**) Folding of stacked DAN–NDI amphiphilic foldamer (DAN in red; NDI in blue) (adapted with permission from Ref. [250]); (**c**) Proposed model for the formation of fibrils from DAN–NDI amphiphilic foldamer upon heating—after unfolding of face-centered DAN–NDI stacked foldamers, the formation of amphiphilic ribbons occurs, which in turn self-assemble by burying leucine side chains in the interior, thus minimizing the lipophilic surface exposed to the aqueous environment, while the hydrophilic aspartate side chains are faced toward the solvent (DAN and NDI units are reported as in panel (**b**), while light blue rectangles are aspartate side chains and orange rectangles are leucine side chains) (adapted with permission from Ref. [250]); (**d**) General structure of Aib-Amb α/γ-foldamers (n = 0, 1, 3 and 7) (Ref. [252]); (**e**) Structure of Boc-(Aib-Aca)_2_-OMe α/ε-foldamer (Ref. [254]); (**f**) General structure of Boc-Gpn-Aib-Xaa-Aib-OMe α/γ-foldamers (Ref. [256]); (**g**) SEM images of (left) microspheres formed by the unsaturated α,ε-hybrid tetrapeptide and (right) ribbon-like entangled fibers formed in *p*-xylene by the saturated α,ε-hybrid tetrapeptide (adapted with permission from Ref. [254]); (**h**) General structure of Boc-Aic_n_-OEt γ-foldamers (Ref. [257]).

Vesicles with different morphologies were obtained by the octapeptide Piv-(Pro-Ant)_4_-OMe (Piv: pivaloyl; Ant: anthranilic acid, 2-aminobenzoic acid), in which anthranilic acid residues were derivatized as ethynylureas to favor the formation of higher-order supramolecular architectures by intermolecular hydrogen bonding, as shown in Figure 22a [246]. Due to the steric requirements of proline, the molecules did not fold forming the usual intraresidue six-membered hydrogen bonded pseudocycles of Ant residues, but assumed a helical conformation characterized by nine-membered H-bonds between NH of Ant(*i*) and CO of Pro(*i*+1) [247]. SEM, TEM and AFM analyses showed that varying the solvent system from methanol to 0.8:9.2 methanol/toluene caused the vesicles’ morphologies to gradually change from hollow spheres to pot-like structures. Moreover, the spherical assemblies were able to encapsulate curcumin and release it upon the addition of tetrabutylammonium fluoride, as imaged by fluorescence microscopy.

#### 4.2.5. Fibers

##### Mixed α–Δβ Foldamers in Different Solvents

Photoswitchable α–Δβ foldamers containing single [248] or multiple (up to four) units of Δ*^Z^*β-Ala ((*Z*)-3-aminoprop-2-enoic acid, the C^α,β^-unsaturated analog of β-Ala) [249] were studied in water and acetonitrile, respectively. The singly Δ*^Z^*β-Ala-substituted peptide Cbz-(Ala-Aib)_2_-Ala-Gly-Δ*^Z^*β-Ala-Lys-Leu-Val-Phe-Phe-OH was irradiated at 290–320 nm in water, and the stable hydrogel was submitted to TEM analysis with uranyl acetate staining. In 20 min, the initially present (mainly) nanocrystalline aggregates were gradually converted to fibers, due to the extensive *Z* to *E* isomerization (95% *E*) ascertained by reverse phase HPLC (RP-HPLC). According to the proposed model, the *E* configuration of the Δ*^E^*β-Ala unit and the β-amyloid Ala-Lys-Leu-Val-Phe-Phe sequence should allow for extensive intermolecular hydrogen bonding and give rise to antiparallel β-sheet formation without detrimental interference from the initial Aib-rich 3_10_-helical portion. On the contrary, in the *Z* isomer, the intraresidue N-H···O=C interaction of Δ*^Z^*β-Ala, experimentally demonstrated by single-crystal XRD and ^1^H NMR in CD_3_CN solution, simultaneously led to the loss of two potential intermolecular hydrogen bonds and to a bent overall structure, for which no higher aggregations of dimers should be possible. In fact, after 10 min irradiation at 254 nm of hydrogel containing 95% *E* isomer, the equilibration to a 6:4 *E*/*Z* mixture led to an almost complete fiber dissociation and hydrogel destruction [248].

In a further study, the same group synthesized foldamers Boc-Val-(Δ*^Z^*β-Ala)_n_-Leu-OMe (n = 1–4), with central sequences of Δ*^Z^*βAla, flanked by two β-sheet-inducing α-amino acids, such as valine and leucine [249]. Even in this case, X-ray diffraction on crystals and NMR spectra in deuteroacetonitrile confirmed the planar disposition of all the Δ*^Z^*β-Ala units, whose NH and CO groups were involved in intraresidue hydrogen bonds (C_6_ pseudocycles), so that intermolecular H-bonding was impossible, in spite of their sheet-like arrangements, due to the all-*Z* sequence. Foldamers (n = 1–4) where then submitted to all-*Z*−all-*E* photoisomerization by UV–B irradiation in CD_3_CN, leading to the quantitative generation of *E* isomer for n = 1 and mixtures with predominant all-*E* configuration for n = 2–4, even though the RP-HPLC-derived percentage of all-*E* isomers decreased with the length. While no fiber formation was reported for longer oligomers, the trimeric foldamer (n = 1) was able to form regular fibers and gelate CD_3_CN after only 10 min irradiation, when the *E*/*Z* ratio was 8:2. As in the previous study [248], the extended β-sheet-like backbone conformation and the additional possibilities for intermolecular hydrogen bonding caused by the *E* configuration of central Δ*^E^*β-Ala residue were on the basis of fiber formation.

##### Mixed Aliphatic–Aromatic Foldamers in Aqueous Environment

DAN–NDI (dialkxoynaphthalene–naphthalenetetracarboxylic diimide) stacked amphiphilic foldamers, as shown in Figure 22b, showed face-centered electron donor–acceptor π–π interactions in aqueous solution. However, UV, visible and CD spectroscopies, as well as dynamic light scattering, evidenced that the intramolecular aggregates disassembled and led to amphiphilic coils on increasing the temperature [250,251]. Coils aggregated to amphiphilic ribbons through NDI–NDI off-set parallel stacking, whereas the data did not allow us to determine the arrangement of DAN units. Then, extensive SEM, TEM, and AFM analyses suggested that the ribbons further dimerized in a bilayer-like fashion to twisted fibrils, in order to maximize the reduction in lipophilic portions (Leu side chains), exposed to the aqueous environment, as shown in Figure 22c, as confirmed by the agreement between the fibril widths and the extended chain lengths [251].

##### Mixed Aliphatic–Aromatic Foldamers in Organic Solvents

Oligopeptides made of two alternating conformationally constrained residues, Aib and the aromatic γ-amino acid 3-amino-5-bromo-2-methoxy benzoic acid (Amb), as shown in Figure 22d, gave length-dependent self-assembled morphologies, likely based on the fully extended conformation observed in crystals and solution by X-ray diffraction and NMR experiments, respectively [252]. The hierarchical process of self-aggregation from non-polar solvents was not the subject of an in-depth study, but a decreasing grade of molecular order was evidenced as the length of oligomers increased. A needle-shaped morphology was seen at TEM for crystals generated by the tetramer (n = 1; particle size ca. 400 nm), while more elongated but less crystalline material was obtained from the octamer (n = 3; particle length ca. 3 µm), and entangled nanofibers were given by the hexadecamer (n = 7; many micrometers in length).

##### Aib-Containing Aliphatic Oligomers in Organic Solvents

A canonical right-handed 3_10_-helix, whose presence was highlighted in the crystal by XRD, also formed in solution by a heptapeptide containing five central Aib residues and two metal-binding 4-pyridyl alanines at both termini, as demonstrated by NMR and ATR FT-IR analyses [253]. The heptapeptide was shown by HR-SEM to form different fibers when silver salts were added, depending on the solvent, and the energy-dispersive spectroscopy (SEM-EDS) allowed us to confirm the inclusion of silver. The counterion had no effect on the outcome, but acetonitrile did not allow fiber formation, due to competition with pyridine moieties for coordination with Ag^+^, and metallo-organic fibers with quite regular size and length up to 1 mm were obtained in ethanol. On the other hand, THF gave much more size dispersity, whereas methanol and DMF produced gel-like materials characterized by the presence of less defined and thinner fibers. However, it is very likely that the 3_10_-helix was conserved in all fibers, with the molecules having an antiparallel arrangement in order to experience stabilizing dipole–dipole interactions among macrodipoles.

The absence of intramolecular hydrogen bonding characterized a peculiar organogel-forming α,ε-hybrid tetrapeptide, showing alternating residues of Aib and the aromatic ε-amino acid AcaH (3-(3-aminophenyl)propionic acid, indicated as the hydrogenated form of its precursor Aca, (*E*)-3-aminocinnamic acid), as shown in Figure 22e [254]. According to UV–vis and FT-IR spectroscopies, in organic solvents (e.g., *p*-xylene, toluene and 1,2-dichlorobenzene) intermolecular hydrogen bonding, and possibly also the π–π stacking interactions observed by single-crystal XRD, drove the fast formation of highly stable organogels, also called xerogels, containing ribbon-like fibers (ca. 500 nm in diameter and several micrometers in length for the xylene gel). On the contrary, FE-SEM images showed that only polydisperse microspheres were obtained by its unsaturated precursor, as shown in Figure 22g.

##### Aib-Containing Aliphatic Oligomers in Organic Solvent–Water Mixtures

Twisted fibers could also be obtained by a particular α-octapeptide based on the repetition of the L-Ala-Aib (Aib: 2-aminoisobutyric acid) sequence with an additional ethylendiamine linker and A and T nucleobases at its termini [255]. The tendency to fold into a stable mixed 3_10_/α-helix, was verified by X-ray diffraction, NMR, and CD spectroscopies. Then, the possibility for Watson–Crick pairing led to a time- and temperature-dependent sequential formation of bent fibrils, protofibers, and twisted fibers from THF–water solutions, which were observed by AFM and TEM microscopies. In addition, the morphology could be varied by the addition of terminators (pristine T) and other species in solution.

The Aib-containing mixed α/γ-tetrapeptides, Boc-Gpn-Aib-Xaa-Aib-OMe (Figure 22f), where Gpn is gabapentin—a constrained achiral γ-amino acid whose β carbon is tethered in a cyclohexyl ring—were also able to generate fibers in different conditions [256]. According to NMR and CD studies, these hybrid tetrapeptides adopted different double turn conformations in solution, which were also found in the crystal by XRD. The double turns always displayed a 12-membered H-bonded pseudocycle between the Boc carbonyl oxygen and the NH of the α-amino acid Xaa (Leu, Phe or Tyr) and a 10-membered H-bonded pseudocycle between the carbonyl oxygen of Gpn and the NH of the fourth residue (Aib). Even if the reasons were not well rationalized, the peptides showed different self-assembling behaviors, especially regarding the spherical or nanorod/nanofiber/twisted nanofiber morphologies formed in methanol–water and THF–water mixtures, respectively, and observed by SEM and TEM microscopies.

##### γ-Foldamers in Different Solvents

Organogels were also formed by the gelating effect of fibers obtained by the dissolution of short γ-peptides based on 4-amino isocaproic acid (Boc-Aic_n_-OEt, n = 2-5), as shown in Figure 22h, in various solvents [257]. According to complete X-ray diffraction and NMR and FT-IR investigations, the oligomers adopted parallel extended polar sheet conformations both in the crystals and in CDCl_3_ solution. These secondary structures were only stabilized by intermolecular hydrogen bonds and were very likely assumed by the longest peptides (n = 4 and 5) also in the xerogel-forming entangled fibers. The fibers observed by field-emission SEM (FE-SEM) and AFM were able to gelate methanol, isopropanol, toluene, benzene, and 95% of either DMSO, DMF, or diglyme in water with a concentration as low as 2 mg mL^−1^. Moreover, rheological studies pointed out that the xerogels had thermoreversible and viscoelastic behaviors, as well as self-healing properties.

##### Peptoid Foldamers in Aqueous Solution

**Figure 23:**
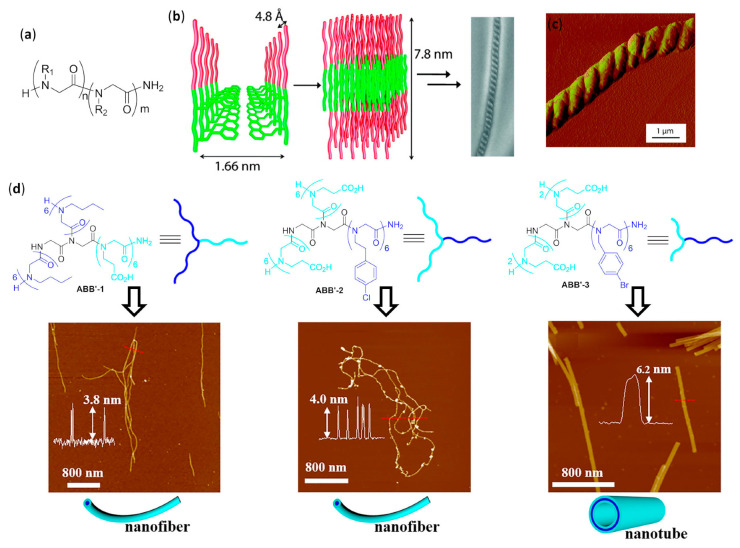
(**a**) General structure of amphiphilic diblock copolypeptoids (R_1_ = lipophilic group, R_2_ = hydrophilic group) (Refs. [58,59,60,61,62,63,64,65,66,67,68,69,70,71,72,73,258]); (**b**) Proposed scheme for the formation of fibers of Npe_15_Nce_15_ in aqueous environment (green: hydrophobic block; red: hydrophilic block): the initially formed monolayers generate bilayers by interdigitating the hydrophobic fully extended chains, which in turn form the right-handed superhelices after further (not characterized) steps (reprinted with permission from Ref. [258]); (**c**) SEM image of a superhelix of Npe_15_Nce_15_ (reprinted with permission from Ref. [258]); (**d**) Chemical structures (hydrophilic chains in cyan and hydrophobic chains in blue; common centric star pivot in black), cartoon representations and AFM images with selected height profiles of hierarchical structures from aqueous self-assembly of TASS peptoids: ABB’-1 (left); ABB’-2 (middle); ABB’-3 (right) (adapted with permission from Ref. [259]).

A study on morphologies formed by amphiphilic diblock copolypeptoids led to the observation of monodisperse homochiral left-handed superhelices from both chiral and achiral foldamers, as evidenced by SEM, TEM, and AFM images [258]. In particular, aqueous solutions at pH 6.8 of the partially charged achiral 30-mer [*N*-(2-phenylethyl)glycine]_15_-[*N*-(2-carboxyethyl)glycine]_15_ (Npe_15_Nce_15_), as shown in Figure 23a, formed superhelices that were most likely double helices, with quite constant diameters (624 ± 69 nm) and half-pitches (606 ± 105 nm), while lengths varied from 2 to 40 μm, as shown in Figure 23c. The hierarchical process, also deduced using X-ray scattering to measure the structural packing parameters in superhelices, initiated with the formation of sheets, whose uniform thickness corresponded to interdigitated bilayers of copolypeptoid fully extended chains, as shown in Figure 23b, while the following steps leading to the final double superhelices could not be fully characterized. Besides the studies on the importance of the two block-specific intermolecular interactions in the self-assembly, the authors evidenced the extremely intriguing, but unexplained, exclusive formation of homochiral left-handed giant superhelices from achiral precursors. Even more impressively, left-handed superhelices were also obtained from copolypeptoids, in which all of the achiral *N*-(2-phenylethyl)glycine (Npe_15_) residues in the lipophilic block were substituted with either *N*-((*R*)-(+)-1-phenylethyl)glycine (Nrpe) or its *S* enantiomer (Nspe). The same result was observed when sodium hydroxide, used to adjust pH, was changed for either *R* or *S N*-1-phenylethyl amine.

#### 4.2.6. Nanotubes

##### Peptoid Foldamers in Aqueous Solution

Peculiar three-armed star-shaped (TASS) peptoids were synthesized by solid-phase synthesis on a common centric star pivot, by attaching three identical chains (AA’A’’-type) or one hydrophilic (or hydrophobic) and two hydrophobic (or hydrophilic) chains (ABB’-type), as shown in Figure 23d [259]. Trifluoroacetates of amphiphilic ABB’-type TASS peptoids were then submitted to self-assembly in aqueous environment by dissolution in a 1:1 water/acetonitrile mixture followed by the slow evaporation of acetonitrile. Nanofibers or single-walled uniform nanotubes, depending on the starting TASS peptoid, were observed by AFM and TEM, as shown in Figure 23d, and more detailed analysis on packing mode was obtained by PXRD spectra. In particular, the packing in ABB’-3 nanotubes, with a diameter of 6.2 nm and a wall thickness of 3.1 nm, was found to be similar to that of related nanotube-forming amphiphilic linear peptoids made of two different blocks featuring the same hydrophilic (*N*-(2-carboxyethyl)glycine, Nce) and hydrophobic (*N*-[(4-bromophenyl)methyl]glycine, Nbpm) monomers [260]. In both cases, an efficient π-stacking and the presence of 4-bromophenyl moieties were ascertained to be crucial for stabilizing the interdigitating extended hydrophobic chains in the wall bilayer, albeit the exact reasons for the necessity of Nbpm units were not determined. However, in the case of linear peptoids, the assembly pathway of peptoid nanotubes was demonstrated to first involve a very fast formation of nanospheres after dissolution into the water–acetonitrile mixture, followed by an increase in nanosphere size and the appearance of nanoribbons, which then started rolling-up one edge and eventually slowly folded and converted to nanotubes. In addition, the extremely stiff nanotubes obtained from linear peptoids could be submitted to a pH-driven reversible variation of diameter and were shown to be amenable to useful functionalization with peptide recognition sequences, dyes, and hosts, such as β-cyclodextrin, in order to endow them with the desired activity [260].

### 4.3. Self-Assembly into Solid Tridimensional Structures

#### Aliphatic Foldamers with Heterogeneous Backbones in Aqueous Environment

##### Mixed α–β Foldamers

**Figure 24:**
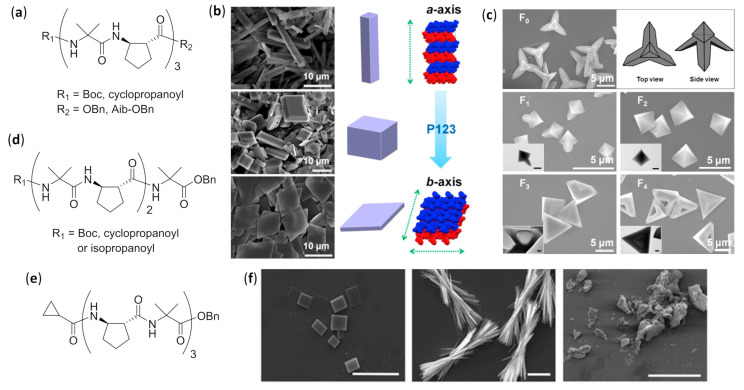
(**a**) General structure of alternating Aib-ACPC hexamers and heptamers (Refs. [261,262,263,264,265]); (**b**) SEM images of foldectures created from Boc-(Aib-ACPC)_3_-OBn added to P123 aqueous solutions at (top) 0.0 g L^−1^, (middle) 0.015 g L^−1^, and (bottom) 8 g L^−1^, and plausible mechanism for the formation of parallelogram plate-shaped foldectures. The common prismatic nucleation network is composed of alternating antiparallel layers (in blue and red) that have the highly hydrophobic faces lying in the *b-c* plane, whereas the hydrophilic faces lie in the two other planes. In pure water, the growth occurs much faster along the *a* direction, in order to avoid the increase in exposed hydrophobic surface (rod-shaped morphology) while in the presence of P123 the growth along axis *a* is suppressed and the elongation occurs in the other two directions (parallelogram plate-shaped foldectures) (adapted with permission from Ref. [261]); (**c**) SEM images of foldectures self-assembled from Boc-(Aib-ACPC)_3_-Aib-OBn in distilled water (F_0_, also with representations) and in 8 g L^−1^ (F_1_), 24 g L^−1^ (F_2_), 40 g L^−1^ (F_3_), and 48 g L^−1^ (F_4_) aqueous solutions of P123 (insets: TEM images with scale bars = 1 µm) (adapted with permission from Ref. [262]); (**d**) General structure of alternating Aib-ACPC pentamers with different caps (Ref. [265]); (**e**) Structure of cyclopropanoyl-(ACPC-Aib)_3_-OBn hexamer (Ref. [265]); (**f**) SEM images of foldectures obtained from pentamers R_1_-(Aib-ACPC)_2_-Aib-OBn in 8 g L^−1^ aqueous solutions of P123 (R_1_ = Boc (left); cyclopropanoyl (middle); isopropanoyl (right)) (adapted with permission from Ref. [265]).

Beyond the foldectures based on 12-helices of β-foldamers already reported in Section 2.3, Lee’s group also extensively studied the aqueous self-assembly in solid shapes of surfactant-containing solutions of 1:1 alternating α–β foldamers showing the repetition of the Aib-*trans*-ACPC sequence, as shown in Figure 24a [261,262,263,264,265]. In all of these studies, the exact packing mode could be ascertained by an intelligent use of high-resolution powder X-ray diffraction (PXRD), whereas the morphologies of self-assembled nanostructures were observed by SEM and additional information could be obtained by applying various physico-chemical analyses, such as differential scanning calorimetry (DSC), thermogravimetric analysis (TGA), and FT-IR.

The addition of a concentrated THF stock solution of 11-helix hexamer Boc-(Aib-ACPC)_3_-OBn, depicted by R_2_ = OBn in Figure 24a, to excess aqueous solutions of nonionic surfactant Pluronic 123 (P123) at different concentrations (0, 0.015 and 8 g L^−1^) led to foldectures varying from long rods with a parallelogram section (in distilled water) to parallelogram plate shapes (in the most concentrated P123 solution) [261]. As in the other cases previously reported for β-peptides, starting from the common prismatic nucleation network characterized by the maximization of hydrophobic packing, in pure water the growth occurred much faster on the two most apolar faces, thus obtaining the rod-shaped morphology. On the contrary, in the presence of P123, the effective solvation of the two opposite apolar faces suppressed the elongation in that direction and favored the growth on the other (polar) faces, therefore leading to the parallelogram plate shaped foldectures, as shown in Figure 24b. In all of the morphologies formed, PXRD analysis confirmed that the secondary structure was almost identical to that in the crystal.

Adding an Aib residue at the C-terminus, thus using the heptamer Boc-(Aib-ACPC)_3_-Aib-OBn, depicted by R_2_ = Aib-OBn in Figure 24a, and exploiting again the differential solvation in water or aqueous P123 solutions at increasing concentrations (8, 24, 40, and 48 g L^−1^) to drive the directional growth, it was possible to synthesize amazing self-assembled shapes with the same molecular packing motif [262]. In particular, the tripod (F_0_) morphology was obtained from distilled water, while the morphologies from F_1_ to F_4_, which were all based on a trigonal bipyramid, formed in 8 g L^−1^ (caudate trigonal bipyramid), 24 g L^−1^ (trigonal bipyramid), 40 g L^−1^ (truncated trigonal bipyramid), and 48 g L^−1^ (truncated trigonal bipyramid with caved face) solutions of P123, respectively, as shown in Figure 24c. The foldectures created, having a three-fold symmetry, were directly due to the layered growth around the molecular packing motif, characterized by three crystallographically equivalent molecules disposed at 120°—one with respect to the others and interacting in a head-to-tail fashion by H-bonding and hydrophobic interactions. The uneven solvation of P123, which even in this case was due to the anisotropic intermolecular packing mode, suppressed at a larger extent the equatorial growth, and the consequent greater axial growth rate caused the observed variations in foldecture shapes. Interestingly, the truncated trigonal bipyramid F_3_ had a large hollow interior, shown in the TEM inset in Figure 24c, whose formation was not explained, and confocal laser scanning microscopy (CLSM) showed that it was possible to encapsulate different fluorescent guests (quantum dots, Rhodamine B, and green fluorescent protein) in a guest-independent manner and without changing the morphology.

In addition, when stock solutions in THF or methanol containing the equimolar mixtures of both enantiomers of Boc-(Aib-ACPC)_3_-Aib-OBn, depicted by R_2_ = Aib-OBn in Figure 24a, were added to aqueous P123 (24 g L^−1^), foldectures different from those obtained from pure enantiomers were generated [263]. Performing experiments with non-equimolar stock solutions, the authors also demonstrated that interactions between mirror images were favored with respect to those among molecules with the same chirality, because two distinct morphologies—one for the equimolar mixture and the other for the excess of pure enantiomer—could be separately observed. An extensive PXRD analysis led to the exact determination of unit cell and packing motif in the elongated parallelogram plate morphology deriving from 1:1 racemic mixtures [264]. The packing motif in the rapidly self-assembled foldecture (nonequilibrium conditions) was identical to that evidenced by X-ray diffraction in single crystals (slow self-assembly from equilibrium conditions), which was instead quite a rare finding for enantiopure foldamers. Within the foldecture, two head-to-tail intermolecular H-bonds were present between foldamers with the same chirality, thus leading to homochiral chains, which in turn experienced close hydrophobic contacts with both parallel chains of the opposite enantiomer and antiparallel chains of the same enantiomer. Once again, the greatly different P123 solvation of faces with various polarities led to the observed morphology.

In a further work, the same authors exploited the addition of THF stock solutions to aqueous P123 surfactant (8 g L^−1^) to study the effect of the substitution of N-terminal Boc with different capping groups in the heptamer (i.e., cyclopropanoyl-(Aib-ACPC)_3_-Aib-OBn, R_1_ = cyclopropanoyl, R_2_ = Aib-OBn), shown in Figure 24a, as well as in shorter oligomers, synthesizing pentamers R_1_-(Aib-ACPC)_2_-Aib-OBn (R_1_ = Boc, cyclopropanoyl or isopropanoyl), as shown in Figure 24d, and the hexamer cyclopropanoyl-(ACPC-Aib)_3_-OBn, as shown in Figure 24e [265]. As evidenced for the three differently N-terminal-derivatized pentamers, the change in capping group had a remarkable influence on the foldectures observed at SEM, as shown in Figure 24f. A great variability of secondary structures and packing motifs was obtained among the three pentamers in both crystals and rapidly self-assembled foldectures by comparison of XRD and PXRD data. In addition, both secondary and quaternary structures for a given compound were also markedly different between equilibrium and nonequilibrium processes. However, cyclopropanoyl capping was shown to be superior in terms of self-assembly strength. Within these derivatives, the NH of the first residue and the electron-poor C^α^-H of cyclopropanoyl capping always formed the same pattern of three-centered hydrogen bonds, N-H···O=C/C^α^-H···O=C, in the head-to-tail interaction with the carbonyl group of the last peptide bond of another foldamer molecule, thus greatly enhancing the self-assembly propensities with respect to the Boc group. The classical N-H···O=C H-bond contributed for about 7 kcal mol^−1^, while the additional strength of interaction due to the C^α^-H···O=C nonclassical hydrogen bond was estimated to be about 3 kcal mol^−1^.

The effect of grafting an α-dipeptide sequence (Leu–Leu) at the C-terminus of an otherwise purely β-peptidic structure, the previously studied Boc-ACPC_6_-OBn [132] was ascertained together with the correlation between the perturbations introduced in the innate 12-helix folding of ACPC oligomers and the modifications in the morphologies observed for Boc-ACPC_6_-Leu_2_-OBn, always carrying out the experiments in aqueous P123 [266]. The folding assumed by the grafted foldamer was a 12-helix within the β region and an 11-helix within the α fragment, and this structural perturbation drastically changed the morphology with respect to the molar tooth-shaped morphologies obtained for Boc-ACPC_6_-OBn, depicted by the M_P_ foldecture in Figure 8d. HR-TEM and AFM analyses showed that the elongated hexagonal plate shapes self-assembled from Boc-ACPC_6_-Leu_2_-OBn were very homogeneous in size, perfectly flat and with a remarkably constant thickness (22 nm). The investigation of molecular packing modes by PXRD and grazing-incidence wide angle X-ray scattering (GIWAXS) evidenced that for both foldamers the self-assembly driving forces were the expected head-to-tail hydrogen bonding patterns and close hydrophobic interactions. However, while, for Boc-ACPC_6_-OBn, an all-parallel arrangement was observed, a strikingly different and highly anisotropic intermolecular arrangement was evidenced for Boc-ACPC_6_-Leu_2_-OBn. In fact, in the latter case, both parallel and antiparallel rows were aligned, and hydrophobic portions were almost exclusively exposed on two opposite facets that, due to extremely uneven solvation exerted by P123, essentially did not grow and ultimately led to the observed elongated hexagonal plates.

##### Mixed α–γ Foldamers

The hierarchical self-assembly of a number of right-handed 12-helices formed by 1:1 alternating α,γ^4^-hybrid hexa- and heptapeptides was studied, exploiting only γ residues obtained from bis-homologated hydrophobic proteinogenic α-amino acids (e.g., γ^4^-Phe, γ^4^-Leu, and γ^4^-Val) and Aib or Ala as the α-AAs [267]. All the hybrid peptides had an acetylated N-terminus and a carboxamide as the C-terminus, and their THF stock solutions were added to distilled water. Even if an in-depth investigation of foldamer disposition in the self-assembled foldectures was not carried out, some general observations could be made. In all cases, the head-to-tail H-bonding was a driving force for the self-assembly, as deduced by XRD in single-crystals. Moreover, regardless of the presence of Ala or Aib as α-amino acids, the heptapeptides featuring three γ^4^-Phe residues only formed nanotubular morphologies, according to high-resolution imaging with field-emission SEM and TEM. The peptide nanostructures showed either a hollow interior or superstructures in which a larger nanotube contained a smaller one, and both closed and open ends could be found. In addition to the head-to-tail H-bonding and some weak π-stackings, this tendency to form nanotubes was mainly ascribed to the stabilizing interhelical CH–π interactions. These unconventional hydrogen bonds occurred among phenyl rings and protons belonging to either backbone C^α^, aromatic CH of γ^4^-Phe or benzylic protons, and were favored by the peculiar and fixed positions of γ^4^-Phe side chains along the 12-helices. On the other hand, when γ^4^-Val was used for all the γ residues, its involvement in the hydrophobic packing led to nanosheets (hexapeptide) or coiled nanofibers (heptapeptide), while for γ^4^-Leu, a random formation of different 3D morphologies occurred.

As a continuation of this study, the authors evaluated the morphologies created by 1:1 alternating α,γ^4,4^-hybrid heptapeptides with the same N- and C-termini previously used, but with achiral 4-aminoisocaproic acid (Aic, which is basically a bis-homologated Aib) as the γ-AA, and Phe as the phenyl-bearing α-AA [268]. In particular, superstructures formed by 60:40 methanol/water solutions of peptides Ac-(Aic-Phe)_3_-Aic-NH_2_ and Ac-(Phe-Aic)_3_-Phe-NH_2_ were compared with those obtained for their constitutional isomer Ac-(Aib-γ^4^-Phe)_3_-Aib-NH_2_, which previously only formed nanotubes when its THF stock solution was diluted in water [267]. According to NMR and CD analyses, both Aic-Phe hybrid peptides showed extended strands as the preferred secondary structures in solution, and they self-assembled by extensive intermolecular hydrogen bonding and either CH–π or π–π interactions, forming thermally stable capsules (up to 150 °C). Energy dispersive X-ray spectroscopy (EDX) confirmed that the capsules were composed only by peptides, while SEM, TEM and AFM imaging, as well as DLS measurements, all furnished a size in the range 200-600 nm, and an amorphous nature was instead suggested by PXRD analysis. On the contrary, under identical conditions, the 12-helices formed by the previously studied Ac-(Aib-γ^4^-Phe)_3_-Aib-NH_2_ peptide led to various three-dimensional crystalline polyhedrons. The capsules were shown to be stimuli-responsive to different salts and resistant to proteinase K, while their shape could be pH-driven. In addition, confocal microscopy evidenced their ability to encapsulate carboxyfluorescein, and the capsules could also be subjected to stimuli-controlled release by using a cationic dipeptide.

### 4.4. Challenges in Design and Structural Characterization of Other Self-Assembled Foldamers

Given the inclusion within this chapter of extremely different foldamer classes, the remaining challenges must be referred to two different subgroups (extensively and non-extensively studied foldamers).

For extensively studied foldamers that have also been the subject of in-depth investigations about their folding propensities, such as peptoids, α/β-peptide, and urea foldamers, the rational design of desired hierarchical superstructures has now reached a high level of reliability. As an illustrative example, a large variety of structures has been obtained for peptoids, some of them being unprecedented, and by using peptoid nanosheets it has been possible to achieve very complex biological functions (e.g., recognition in biological environment of different biomolecules, toxins, and antibodies), thus tackling a very difficult challenge. Albeit much less developed, few cases of biostructure-mimicking self-assembled foldamers have been reported for α–β and urea foldamers in subsections dealing with supramolecular structures of different shape and size. For other foldamers that have only been studied by few research groups, the possibility for a successful rational design seems to be more limited, obviously due to the lack of fundamental information, and the field of biomimicking is yet almost unexplored. However, in general, designing self-assembling foldamers for practical applications still remains a major challenge.

Curiously, also from the point of view of an accurate structural determination, the difference in the level of results reported for different foldamers seems to parallel quite closely that in their basic knowledge. In fact, the in-depth structural investigation of peptides, α/β-peptide and urea foldamers by large groups with cutting-edge instrumentation and procedures often leads to molecularly precise structural models, even for difficult-to-treat cases (e.g., fibers, vesicles, bundles in solution).

Simple dimeric arrangements in solution of more rarely studied foldamers are an exception, in that they are guided by the positioning of hydrogen bonding groups and are thus easy to analyze, exploiting mainly NMR data. In all the other cases, it is evident that the major challenge is still represented by the insufficient use of powerful experimental procedures and following data elaboration/fitting/refining, often coupled to well conducted molecular modelling (e.g., low-dose cryo-TEM with sorting/averaging procedures for vesicles, powder X-ray diffraction for nanosheets, fibers, and solids, grazing incidence X-ray diffraction, and various AFM microscopies for SAMs, etc.).

## 5. Conclusions

This review has discussed selected examples of the extremely wide range of self-assemblies reported so far in literature for different kinds of foldamers, the synthetic oligomers that possess enhanced folding abilities in comparison with their naturally occurring counterparts (e.g., peptides, proteins, nucleic acids) without their intrinsic flaws (e.g., proteolytic susceptibility, conformational disorder for short sequences). Foldamers can globally compete with natural macromolecules and other synthetic frameworks in terms of ease of synthesis and functionalization, and they can be rationally designed and finely tailored to adopt the desired conformation and associate in a higher-order supramolecular structure through weak intermolecular interactions.

Even if self-assembly is, by definition, an intermolecular process, the conditio sine qua non to direct the intermolecular behavior of foldamers is the capability to first govern their intramolecular forces. The formation of the reported multitude of self-assembled structures mainly relies on the ability to wisely exploit the innate, but still easily tunable, tendency of a given class of foldamers to assume a precise secondary structure. The secondary structure can also be endowed with the desired flexibility and ability to fold/unfold, together with a positioning of side chains aimed at properly interacting with other foldamer molecules. Thus, as demonstrated throughout this review, foldamer self-assembly is intimately based on the rational deployment of the basic features of constituent unit(s), such as monomer lengths and substitutions, types of interconnecting bonds, strength of intramolecular non-covalent interactions (with a special regard for H-bonding), preferred dihedral angles, the presence of additional conformational constrictions, the orientation of functional groups and side chains, and overall backbone flexibility.

The following condition to build the desired higher-order self-assembled construct is the knowledge of the intermolecular weak interactions of foldamers, with both themselves and the environment. As exemplified throughout this review, the entire ensemble of noncovalent interactions must be considered and used rationally when designing foldamer self-assembly, even though usually hydrogen bonds, van der Waals interactions, π–π stacking and hydrophobic effects are predominating.

In addition, the experimental variables (e.g., solvent, concentration, temperature, presence of surfactants or additives) must be carefully chosen in order to make the assembly occur either in equilibrium or nonequilibrium conditions, whose outcomes can be very different. The formation in solution of various duplexes, β-strands, double/multiple helices and oligomeric bundles is an equilibrium process, and the same usually also applies to some higher-order superstructures, such as vesicles. Thus, many particular applications have been reported that are possible on the basis of different equilibria (e.g., association–disassociation, folding–unfolding, threading–unthreading, etc.), and also the permeability of fluid membranes for vesicles. On the other hand, self-assembled monolayers, depending on the particular system and methodology of deposition, can be the result of either an equilibration or an irreversible formation, whereas fibers and solid 3D foldectures are created in nonequilibrium conditions, taking advantage of the uneven solvation of foldamer spatial regions with different polarity.

Eventually, within this review the experimental techniques necessary for the in-depth investigation of such different self-assembled structures have been highlighted. When applicable, single-crystal X-ray diffraction data were always used as a starting point for the elucidation of secondary and higher-order structures. However, the necessity to study soluble supramolecular aggregates with a relatively small (i.e., up to bundles) or large (i.e., vesicles) size, as well as self-assembled monolayers and even insoluble solids generated through nonequilibrium processes (i.e., fibers and 3D morphologies), led to the use of an extremely wide variety of experimental tools, sometimes in all their shades (e.g., NMR experiments in solution, X-ray diffractions for non-crystalline structures and soft matter, variegated AFM measurements).

Considered all together, the studies reported in this review demonstrate that, albeit very recent, foldamer self-assembly is a promising and fast-growing field. Many possible practical implementations and biomedical applications range from nanomaterials to molecular recognition, catalysis, drug delivery, biosensing, biocompatible materials, artificial ion channels, and receptors, and many others could rapidly develop in the near future.
